# A novel pyrrolidine-chalcone derivative exhibits synergistic anti-cervical cancer activity by dual-targeting MDM2-p53 axis and ferroptosis pathway

**DOI:** 10.3389/fphar.2026.1715712

**Published:** 2026-02-06

**Authors:** Hainimu Xiamuxi, XinYi Deng, Mourboul Ablise, Tong Yan, Madina Imam, Yu Wang, MingHui Zu, Wutiekuer Wumaier

**Affiliations:** The Xinjiang Key Laboratory of Natural Medicine Active Components and Drug Release Technology, College of Pharmacy, Xinjiang Medical University, Urumqi, China

**Keywords:** cervical cancer, chalcone, ferroptosis, MDM2-p53, transcriptomics

## Abstract

**Introduction:**

Cervical cancer remains a serious threat to women’s health, driving the need for effective and low-toxicity therapeutics. This study designed novel pyrrolidine-chalcone derivatives targeting the MDM2-p53 interaction.

**Methods:**

A series of compounds were synthesized and evaluated for cytotoxicity against cervical cancer (HeLa, SiHa, C33A) and normal (H8) cells via CCK-8. The lead compound B1 was further analyzed for p53 pathway activation, apoptosis, and ferroptosis markers (ROS, GSH, MDA, Fe^2+^) using western blot, flow cytometry, and assay kits.

**Results:**

Compound B1 showed potent, nanomolar cytotoxicity (IC_50_ = 0.22, 0.24, and 0.95 μM for HeLa, SiHa, and C33A, respectively) with low toxicity to H8 cells. B1 activated p53 by downregulating MDM2, inducing cell cycle arrest and apoptosis. Simultaneously, it triggered ferroptosis via ROS accumulation, GSH depletion, elevated MDA and Fe^2+^, and suppression of SLC7A11/GPX4.

**Discussion:**

B1 is a promising dual-mechanism lead compound against cervical cancer, concurrently activating p53 and ferroptosis, warranting further investigation.

## 1 Introduction

Cervical cancer ranks as the fourth most common malignancy among women worldwide (Sung et al., 2021), and its pathogenesis is closely associated with persistent human papillomavirus (HPV) infection (de Martel et al., 2012). Although vaccination and early screening have significantly reduced incidence, the treatment of advanced or recurrent metastatic cervical cancer remains a major challenge (Tewari et al., 2022; Fokom Domgue and Schmeler, 2019). Common therapies, including surgery, radiotherapy, and platinum-based chemotherapy, are often limited by high tumor drug resistance and their own toxicity (Marabelle et al., 2020), leading to tumor recurrence in approximately 40% of patients with advanced cervical cancer within 5 years (Liontos et al., 2019; Zhao et al., 2021; Levine, 2020). These limitations underscore the pressing need for more effective and less toxic therapeutic strategies.

**FIGURE 1:**
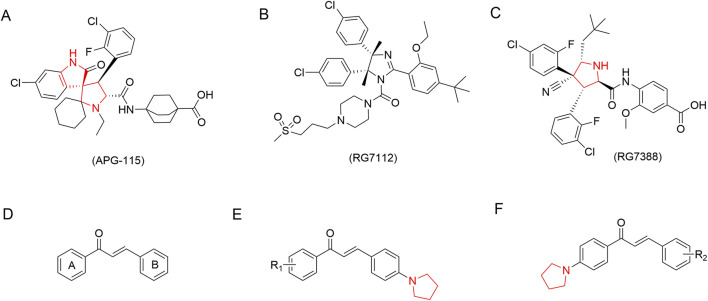
**(A)** APG115; **(B)** RG7112; **(C)** RG7388; **(D)** Chalcone; **(E, F)** are synthesized by our group.

A key molecular driver in cervical cancer is the inactivation of the p53 tumor suppressor pathway (Lane, 1992; Bano et al., 2025; Levine, 2020). In over 90% of cases, the HPV E6 oncoprotein drives the ubiquitin-mediated degradation of p53 (Hashemi et al., 2025; Alimujiang et al., 2024). This process is exacerbated by the frequent overexpression of Murine Double Minute 2 (MDM2), p53’s primary negative regulator, creating a vicious cycle within the “MDM2-p53 negative feedback loop.” Consequently, targeting this pathway to reactivate p53 is a crucial therapeutic strategy (Böttger et al., 1999; Popowicz et al., 2008; Vassilev et al., 2004; Vu et al., 2013; Fang et al., 2021). MDM2 small-molecule inhibitors such as RG7112, RG7388, and APG-115 (Figures 1A-C) have shown preclinical promise, yet their clinical translation is hindered by challenges including dose-limiting toxicity, acquired resistance, and suboptimal pharmacokinetics. For instance, while Nutlin-3a exhibits potent MDM2 inhibition, its specificity is not absolute, as it may influence off-target proteins and signaling pathways, resulting in non-specific effects that compromise therapeutic precision. Furthermore, prolonged Nutlin-3a treatment can induce tumor cell resistance, leading to diminished efficacy over time, and its pharmacokinetic profile often limits effective systemic distribution and sustained action *in vivo* (Blagosklonny, 2023). Similarly, RG7112 requires high doses to achieve therapeutic effect, which frequently leads to adverse events including gastrointestinal intolerance and thrombocytopenia (Hassin and Oren, 2023). Given these limitations, structural innovation remains critical.

Beyond apoptosis, ferroptosis has emerged as a potent mechanism to kill cancer cells. This iron-dependent cell death is driven by lipid peroxide accumulation (Stockwell, 2022; Shi et al., 2021; Ye et al., 2020; Xu et al., 2022; Chen et al., 2022) Cervical cancer cells often develop resistance to it through upregulated GPX4 activity and aberrant glutathione metabolism, contributing to chemoresistance (Xu et al., 2023; Xiong et al., 2022). Disrupting the SLC7A11-GSH-GPX4 axis to induce ferroptosis can selectively target tumor cells and exhibits crosstalk with apoptotic pathways (Alakkal et al., 2022; Wu et al., 2021; Zhao et al., 2022). Intriguingly, p53 can negatively regulate SLC7A11, suggesting a potential synergy between MDM2-p53 pathway activation and ferroptosis induction—a combination that may overcome the compensatory resistance seen with single-target therapies (Bieging et al., 2014; Jiang et al., 2015; Xie et al., 2017; Chen et al., 2022; Zhai et al., 2021; Mao et al., 2016).

Chalcones, Figure 1D a class of natural α,β-unsaturated ketones, represent a promising antitumor scaffold capable of modulating multiple pathways, partly due to their Michael acceptor properties (Kubiak et al., 2025; Nivedya et al., 2025; Quan et al., 2025; Neves et al., 2012; Dong et al., 2022; Bunsick et al., 2025). However, their development has been limited by modest potency and selectivity. Structural hybridization with privileged pharmacophores is a proven strategy to overcome these limitations (Yang et al., 2023; Liu et al., 2024a; Liu et al., 2024b). Now the structural optimization of traditional chalcone analogs against cervical cancer has largely focused on peripheral aromatic ring substitutions (e.g., hydroxylation, methoxylation, or prenylation), while the strategic hybridization of the core chalcone scaffold with saturated nitrogen heterocycles has remained underexplored (Nikolic et al., 2025; Alimujiang et al., 2024). Moreover, a significant limitation persists: most reported chalcone derivatives exhibit only moderate anti-cervical cancer potency, with IC_50_ values typically in the micromolar range, which restricts their translational potential. Notably, the pyrrolidine moiety is a versatile scaffold present in many clinical agents and several reported MDM2 inhibitors, known to enhance target affinity, solubility, and metabolic stability (Ding et al., 2013).

Based on this scientific background, this study proposes a design concept centered on “pyrrolidine-chalcone hybrid molecules” Figures 1E,F. The incorporation of the pyrrolidine moiety is designed to enhance binding to MDM2 through complementary steric and electronic effects. Sterically, its puckered conformation mimics key p53 peptide residues (Jeelan, et al., 2022) (e.g., Phe19, Trp23, Leu26), enabling effective occupation of the hydrophobic cleft on MDM2 and improving van der Waals contacts. Electronically, the secondary amine can act as a hydrogen-bond donor to MDM2 (e.g., with Leu54) and modulate electron density of the adjacent chalcone, fine-tuning non-covalent interactions with surrounding residues. This targeted design strengthens MDM2 binding while retaining the chalcone’s Michael acceptor property, enabling multi-target intervention. Through systematic SAR studies, we synthesized a series of novel derivatives that simultaneously activate p53-dependent apoptosis and induce ferroptosis, achieving synergistic anti-cervical cancer effects. This study reports the novel dual-mechanism antitumor action of compound **B1**, proposing a potential strategy to address challenges in cervical cancer targeted therapy.

## 2 Results and discussion

### 2.1 Synthesis of 24 novel pyrrolidine-chalcone derivatives

Targeting the MDM2-p53 interaction pathway represents a crucial strategy for developing anti-cervical cancer drugs, particularly for p53 wild-type cases. Based on this rationale, we designed a series of chalcone derivatives featuring a Michael acceptor scaffold (α,β-unsaturated ketone structure). The initial compound library (**A1**–**A9**) incorporated a pyrrolidine moiety at the four-position of ring B and introduced substituents such as methoxy, halogens, and trifluoromethyl on ring A to investigate electronic and steric effects. **A1** exhibited significant activity against cervical cancer cells (HeLa, SiHa, C-33A) and low toxicity towards normal cervical epithelial cells (H8), highlighting the critical role of the 2-methoxy group. The favorable activity of the halogen-substituted compound **A5** (2-CF_3_) inspired us to introduce halogens at the five-position while retaining the 2-methoxy group, leading to the design of the B series (**B1**–**B7**). Among them, **B1** (5-F-2-OCH_3_) demonstrated exceptional potency (HeLa/SiHa IC_50_ ≈ 0.22/0.24 μM), surpassing all previous compounds. Subsequent optimization replaced the 2-methoxy group with trifluoromethoxy (OCF_3_) at different positions (**B5**–**B7**), revealing that 2-OCF_3_ (**B5**) still maintained significant activity. To evaluate the impact of disubstitution patterns, we synthesized 2,5-difluoro (**C1**) and 2,5-dimethoxy (**C2**) analogues, but both showed lower activity than **B1**. Finally, to probe the effect of substituent symmetry, we interchanged the substituents on rings A and B, designing the D series (**D1**–**D6**). Although **D1**–**D3** exhibited moderate activity, none surpassed the activity of compound **B1**.

**SCHEME 1:**
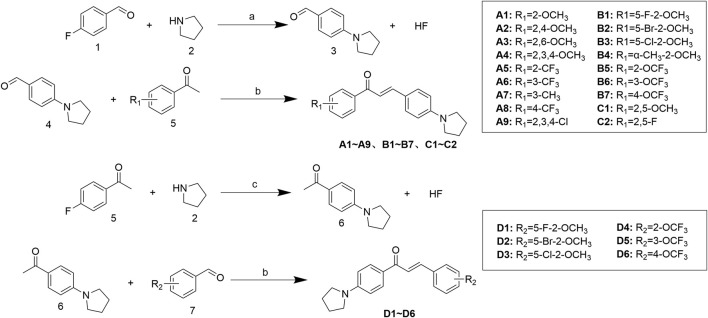
**(a)** DMF, 60 °C, TEA, 81.5%; **(b)** EtOH, r.t, KOH, 41.6%–78.7%; **(c)** DMF, 60 °C, TEA, 76.4%.

The structures of all synthesized target derivatives (Scheme 1) were characterized by ^1^H nuclear magnetic resonance (NMR), ^13^C NMR, and HR-MS, with spectral data consistent with the predicted structures. Notably, an improvement was made to the traditional synthesis procedure: instead of adding basic solutions like KOH or NaOH, solid base was directly added with ultrasonic treatment, resulting in shortened reaction times. All 24 compounds were synthesized within 4 h.

### 2.2 Biological evaluation

#### 2.2.1 Analysis of the *in vitro* antiproliferative activity and structure activity relationship (SAR) of the 24 pyrrolidine-chalcone derivatives

The antitumor activity of all synthesized compounds was evaluated *in vitro* against the HeLa, SiHa, C-33A, and H8 cell lines using the Cell Counting Kit-8 (CCK-8) assay, with cisplatin and Nutlin-3a as positive controls. The IC_50_ values are summarized in Table 1.

**TABLE 1:** Inhibition rates of compounds on proliferation of different cells.

Compd.	IC_50_/(μmol·L^−1^)			
	HeLa	SiHa	C-33A	H8
**A1**	3.04 ± 0.03	2.93 ± 0.21	4.79 ± 0.19	61.31 ± 1.34
**A2**	16.66 ± 0.65	18.32 ± 0.73	17.89 ± 0.55	74.01 ± 1.05
**A3**	31.50 ± 1.46	64.44 ± 0.57	>100	>100
**A4**	15.70 ± 0.38	7.78 ± 0.24	21.26 ± 1.02	81.02 ± 1.03
**A5**	13.56 ± 0.26	37.95 ± 0.74	36.69 ± 0.22	78.73 ± 1.41
**A6**	31.76 ± 1.45	34.48 ± 0.59	35.12 ± 0.37	86.39 ± 3.17
**A7**	27.58 ± 2.23	16.41 ± 0.13	37.20 ± 0.19	91.57 ± 3.28
**A8**	>100	>100	>100	>100
**A9**	>100	90.46 ± 3.38	>100	>100
**B1**	0.22 ± 0.01	0.24 ± 0.03	0.95 ± 0.02	45.77 ± 1.61
**B2**	1.09 ± 0.05	1.35 ± 0.13	4.40 ± 0.11	56.83 ± 0.75
**B3**	1.59 ± 0.08	2.17 ± 0.02	4.29 ± 0.15	57.03 ± 2.47
**B4**	84.76 ± 2.69	44.67 ± 0.78	>100	>100
**B5**	0.90 ± 0.02	0.91 ± 0.03	5.93 ± 0.07	76.91 ± 3.87
**B6**	>100	49.47 ± 1.40	>100	>100
**B7**	>100	>100	>100	>100
**C1**	9.30 ± 0.57	9.92 ± 0.67	13.82 ± 0.45	27.19 ± 2.33
**C2**	27.09 ± 0.59	26.44 ± 1.11	44.10 ± 1.99	35.09 ± 0.95
**D1**	5.25 ± 0.10	5.44 ± 0.23	20.89 ± 1.03	19.22 ± 1.08
**D2**	10.75 ± 0.37	11.55 ± 0.06	49.76 ± 2.93	39.97 ± 0.55
**D3**	8.17 ± 0.46	10.93 ± 0.38	34.20 ± 1.30	22.63 ± 0.32
**D4**	4.09 ± 0.24	9.28 ± 0.33	33.02 ± 2.38	>100
**D5**	31.99 ± 1.28	44.37 ± 1.64	>100	>100
**D6**	>100	77.28 ± 2.13	>100	>100
Cisplatin	18.23 ± 0.11	36.97 ± 1.54	12.71 ± 1.09	25.63 ± 1.32
Chalcone	63.87 ± 2.85	69.67 ± 1.67	58.70 ± 1.05	38.59 ± 1.64
Nutlin-3a	61.30 ± 1.42	56.64 ± 2.20	57.03 ± 2.81	45.46 ± 2.11

Based on the above results, the following SARs were established: (1) Critical Role of Ring A Substituents: Indispensable 2-Substitution: Compounds with electron-donating groups at the two-position of ring A (e.g., **A1**: 2-OCH_3_, IC_50_ = 3.04 μM; **B1**: 2-OCH_3_-5-F, IC_50_ = 0.22 μM) showed significantly superior activity compared with 3/4-position substituted analogues (**A6**, **A8**, **B6**–**B7**; IC_50_ > 100 μM).5-Halogenation Enhances Activity: Introducing F/Cl/Br at the five-position (**B1**–**B3**) dramatically increased activity compared with the mono-substituted A1 with the potency order F > Cl > Br. (2) 2-Position Accommodates Trifluoromethoxy: **B5** (2-OCF_3_) retained nanomolar-level activity (IC_50_ = 0.90 μM), while 3/4-OCF_3_ (**B6**–**B7**) were completely inactive. (3) Steric Hindrance Effect is Negative: Introduction of an α-methyl group (**B4**) significantly reduced activity. (4) 4-Pyrrolidine on Ring B is Optimal: All highly active compounds retained the 4-pyrrolidinyl ring B. Modification of this structure (e.g., substituent interchange in D series) led to decreased activity, suggesting its potential involvement in target binding. (5) Impact of Disubstitution Patterns: 2,5-Disubstitution: Both 2,5-dimethoxy (**C2**, IC_50_ = 9.30 μM) and 2,5-difluoro (**C1**, IC_50_ = 27.09 μM) were weaker than the mono-substituted **B1**, indicating a synergistic effect between the 5-halogen and 2-methoxy substituents. (6) Advantage of Asymmetric Structure: Interchanging substituents on rings A and B (**D1**–**D6**) resulted in ≥10-fold reduced activity, demonstrating that the spatial orientation of the 2-methoxy/halogen groups is crucial for target binding. (7) Toxicity Profile: **B1** possessed the highest therapeutic index (TI = IC_50_, H_8_/IC_50_, HeLa ≈ 203), significantly superior to **A1** (TI ≈ 20) and other analogues. Although 3/4-position substituted compounds (e.g., **A8**, **B6**–**B7**) showed low cytotoxicity, they had poor selectivity, implying nonspecific effects.

In summary, compound **B1** emerged as the exemplar of this pharmacophore, demonstrating exceptional potential with its nanomolar cytotoxicity against cancer cells (HeLa IC_50_ = 0.22 μM) and >200-fold selectivity over normal H8 cells. Notably, compound **B1** exhibited potent anti-proliferative activity against HeLa cells with an IC_50_ of 0.22 μM, markedly outperforming cisplatin (18.23 μM), its chalcone precursor (63.87 μM), and a known MDM2 inhibitor (61.30 μM). Therefore, **B1** was selected as the lead candidate for further in-depth investigation of its anti-cervical cancer mechanisms and molecular targets.

#### 2.2.2 Compound B1 induces cervical cancer cell apoptosis in a dose dependent manner

**FIGURE 2:**
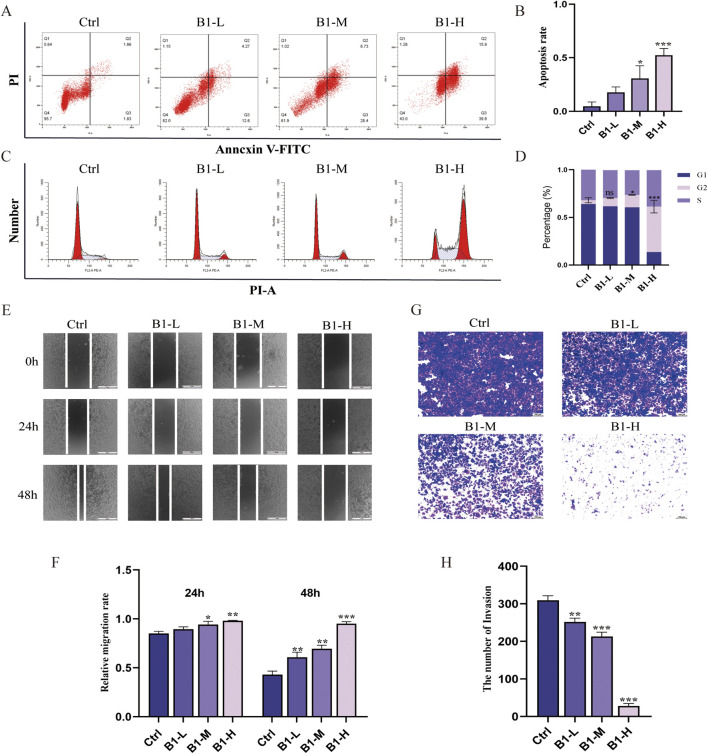
Compound **B1** induced apoptosis **(A, B)**, arrested the cell cycle and **(C, D)** and prevented the migration **(E, F)** and invasion **(G, H)**. n = 3, *p < 0.05, **p < 0.01, ***p < 0.001 vs. “Ctrl” group.

Cell apoptosis plays a vital role in the progress of tumorigenesis and formation. To evaluate the effect of compound **B1** on HeLa cell apoptosis, cells were treated with **B1**-L (0.11 μM), **B1**-M (0.22 μM), and **B1**-H (0.44 μM) for 48 h, followed by flow cytometry analysis (Figures 2A,B). Results showed that compared with the blank group, the apoptosis rate of HeLa cells increased from 4.70% ± 3.56% to 52.24% ± 5.31%, demonstrating that **B1** significantly induces apoptosis in HeLa cells.

#### 2.2.3 Compound B1 induces G2/M cell cycle arrest of the cervical cancer cells

To assess the effect of compound **B1** on the HeLa cell cycle, we performed PI/RNaseA staining assays (Figures 2C,D). After treating HeLa cells with **B1**-L (0.11 μM), **B1**-M (0.22 μM), and **B1**-H (0.44 μM) for 24 h, the percentage of cells in the G_2_/M phase increased from 3.71% ± 2.36% in the control (Ctrl) group to 47.85% ± 5.37%, indicating that **B1** significantly affects the cell cycle, arresting the majority of cells in the G_2_/M phase, thereby exerting its antitumor effect.

#### 2.2.4 Compound B1 induces concentration dependent suppression of cervical cancer cell migration

Cell migration is an important biological feature of tumor metastasis that contributes to malignancy and recurrence. A wound-healing assay revealed that treatment with **B1**-L (0.11 μM), **B1**-M (0.22 μM), and **B1**-H (0.44 μM) for 24 and 48 h significantly reduced the wound closure ability of HeLa cells compared with the control (Ctrl) group, exhibiting a clear concentration-dependent effect (Figures 2E,F).

#### 2.2.5 Compound B1 induces concentration dependent suppression of cervical cancer cell invasion

As the invasive spread of tumor cells is a key pathological process in cancer progression, we sought to determine whether **B1** could interfere with HeLa cell invasion. Using a Matrigel-based Transwell assay, we found that **B1** at concentrations of 0.11, 0.22, and 0.44 μM caused a significant and dose-dependent reduction in invasive capability (Figures 2G,H). This clearly demonstrates that **B1** is a potent inhibitor of HeLa cell invasion *in vitro*.

#### 2.2.6 Molecular docking and dynamics simulation studies

**FIGURE 3:**
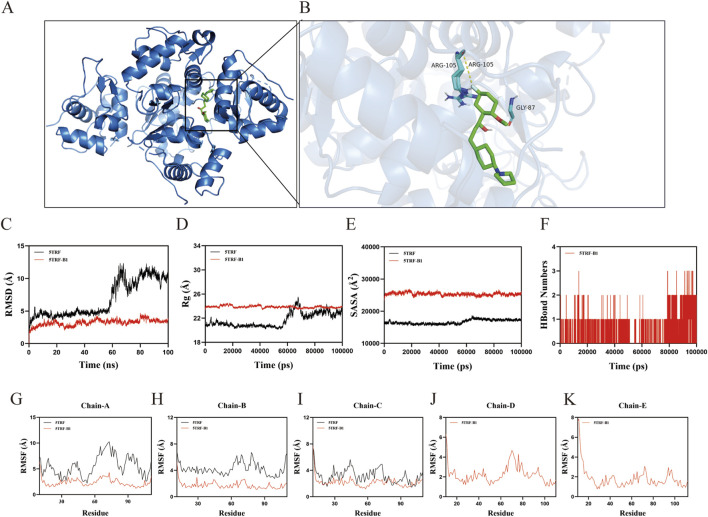
Molecular docking expressions of **B1 (A, B)**, Molecular dynamics simulation experiments of **B1 (C–K)**.

Molecular docking (Table 2) revealed that compound **B1** binds to the MDM2 protein (PDB ID: 5TRF) with a significant affinity (binding energy: −8.2 kcal/mol), occupying the p53-binding pocket through multiple non-covalent interactions. Its hydrophobic groups engaged with residues LEU85, VAL108, LEU107, and VAL109, while key hydrogen bonds were formed with the backbone of GLY87 and the side chain of ARG105. Notably, a fluorine atom on **B1** formed a specific halogen bond with ARG105, enhancing binding specificity. Additional stability was provided by van der Waals contacts with TYR104, LYS98, ASP84, LYS31, THR101, and THR26 (Figures 3A,B).

**TABLE 2:** Molecular docking results and key interactions.

Compound	PDB ID	Grid center (Å)	Grid dimensions (Å)	Binding affinity (kcal/mol)	Hydrogen bonds
**B1**	5TRF	x = −1.29 y = 30.738 z = 32.737	x = 47.25 y = 47.25 z = 47.25	−8.4	GLY87 ARG105

To evaluate the binding stability, molecular dynamics simulations were conducted over 100 ns. The **B1**-bound system achieved a stable, lower RMSD (∼4.0 Å) compared to the more dynamic apo-MDM2 (up to 8.1 Å), indicating enhanced global rigidity upon ligand binding (Figure 3C). This stabilization stems from specific pocket interactions and modulates flexibility. RMSF analysis revealed that high apo-protein fluctuations originate from flexible loops, which are restrained by **B1** (Figures 3G-K). Concurrently, the binding pocket itself forms a rigid, low-fluctuation core. The complex maintained compactness (Rg, Figure 3D) and a shielded interface (SASA, Figure 3E), with one to three persistent hydrogen bonds and a stable halogen bond underpinning the pose (Figure 3F).

In conclusion, simulations demonstrate that **B1** stabilizes MDM2 by locking flexible loops and forming a well-anchored complex via multiple interactions, explaining its potent inhibitory activity.

#### 2.2.7 In silico ADMET profiling of compound B1

The drug-likeness and ADMET properties of compound **B1** were predicted using ADMETlab 2.0. **B1** (MW = 325.15 g/mol) complies with Lipinski’s Rule of Five, exhibits a low topological polar surface area (29.54 Å^2^), and shows good predicted synthetic accessibility (SAscore = 1.969). However, its logP of 4.35 indicates high lipophilicity. **B1** is predicted to have high gastrointestinal absorption and low blood-brain barrier penetration (BBB = 0.158), which is advantageous for reducing CNS side effects. It is a non-substrate but a potential inhibitor of P-glycoprotein (Pgp-inhibitor probability = 0.993). Plasma protein binding is high (99.54%). In terms of toxicity, **B1** shows a low probability of rat oral acute toxicity (0.066), suggesting a favorable acute safety profile. However, significant alerts were identified for Ames mutagenicity (0.927), drug-induced liver injury (0.912), carcinogenicity (0.83), and skin sensitization (0.927). **B1** is predicted to be a substrate for CYP2C9, CYP2D6, and CYP1A2.

In conclusion, while **B1** demonstrates promising drug-like properties in absorption, BBB penetration, and acute toxicity, its high lipophilicity, potential for drug-drug interactions, and specific toxicity risks require further experimental investigation.

#### 2.2.8 Compound B1 reduced the fluorescence intensity of MDM2

**FIGURE 4:**
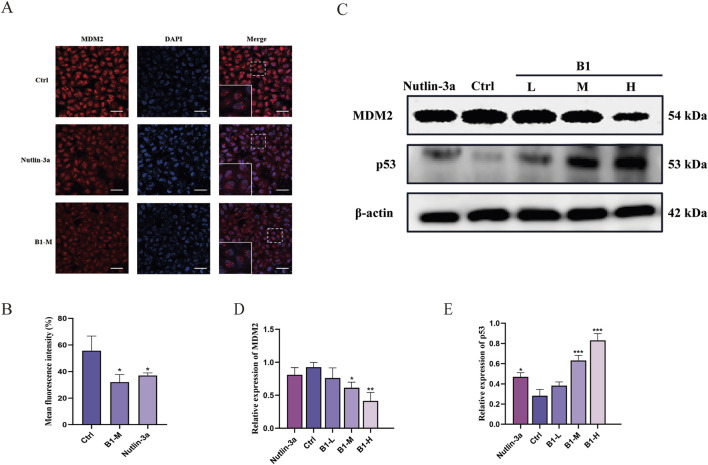
**B1** influenced the fluorescence intensity of MDM2 **(A, B)**, scale bar 50 μm. **B1** influenced the expression of MDM2 and p53 in Hela Cells **(C–E)**, n = 3, *p < 0.05, **p < 0.01, ***p < 0.001 vs. “Ctrl” group.

Immunofluorescence assays assessed the effect of compound **B1** on MDM2 protein fluorescence intensity (Figures 4A,B). Treatment with 0.22 μmol/L **B1** significantly reduced MDM2 fluorescence intensity compared with the blank group, comparable to the effect of the positive control at the same concentration, confirming **B1**’s impact on MDM2 protein expression.

#### 2.2.9 Compound B1 regulated MDM2 and p53 protein expression

Western blot analysis was used to detect the effect of **B1** on MDM2 and p53 protein expression levels. Compared with the blank group, increasing concentrations of **B1** led to decreased MDM2 (Figures 4C,D) and increased p53 protein expression (Figures 4C,E), indicating a significant regulatory effect of **B1** on both proteins.

#### 2.2.10 Transcriptome sequencing analysis

**FIGURE 5:**
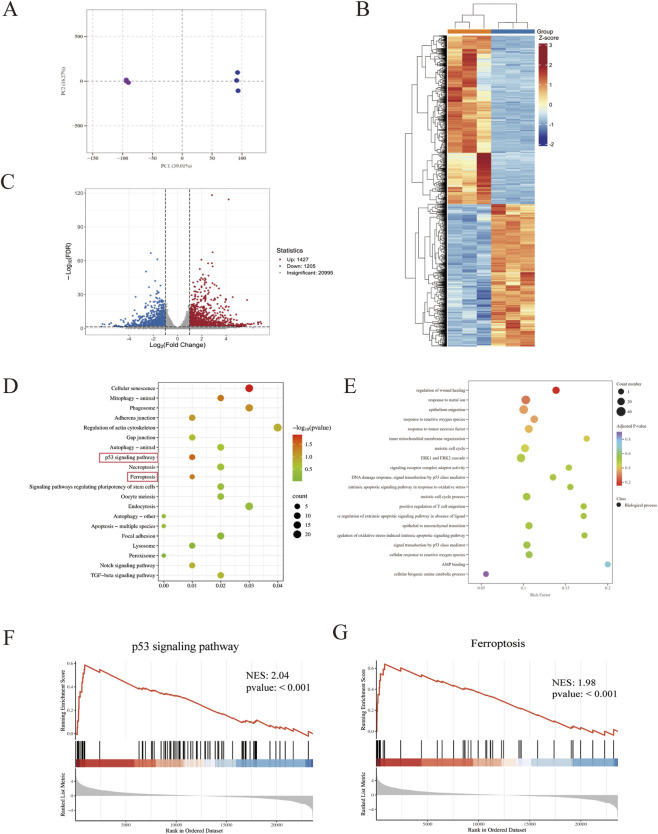
Transcriptome sequencing analysis: principal component analysis **(A)**, differential gene heatmap **(B)**, differential gene volcano plot **(C)**, GO enrichment analysis **(D)**, KEGG enrichment analysis **(E)**, log_2_FC > 1, FDR < 0.05, **(F)**. GSEA enrichment of the p53 signaling pathway gene set **(F)**. GSEA enrichment of the ferroptosis gene set **(G)**.

Principal component analysis (PCA) revealed a clear separation in the transcriptome profiles between compound **B1**-treated cervical cancer cells (right cluster) and the control group (left cluster; Figure 5A). PC1 (contributing 39.01% variance) was the primary driver of inter-group differences, indicating that **B1** treatment triggered global transcriptional reprogramming. High intragroup sample clustering confirmed good experimental reproducibility, while distinct intergroup separation demonstrated consistent **B1**-induced gene expression regulation. The differential gene heatmap (Figure 5B) showed complete separation between **B1**-treated cells (left) and controls (right), confirming drug-induced reshaping of the transcriptional profile. The volcano plot of differentially expressed genes (DEGs; Figure 5C) revealed a large number of significantly altered genes, including 1,427 upregulated and 1,205 downregulated genes. Gene Ontology (GO; Figure 5D) and Kyoto Encyclopedia of Genes and Genomes (KEGG; Figure 5E) enrichment analyses indicated that **B1** influences gene expression through ferroptosis-related pathways, providing a theoretical basis for subsequent ferroptosis targeting studies. Gene Set Enrichment Analysis (GSEA; Figures 5F,G) revealed significant enrichment of gene signatures associated with two key pathways. The p53 signaling pathway was markedly upregulated [Normalized Enrichment Score (NES) = 2.04, FDR q-value <0.001]. Concurrently, a gene set representative of ferroptosis also showed significant enrichment (NES = 1.98, FDR q-value <0.001). These complementary findings provide genomic evidence for the p53-mediated signaling and the ferroptosis process in our experimental model.

#### 2.2.11 Influence of B1 on ferroptosis markers

**FIGURE 6:**
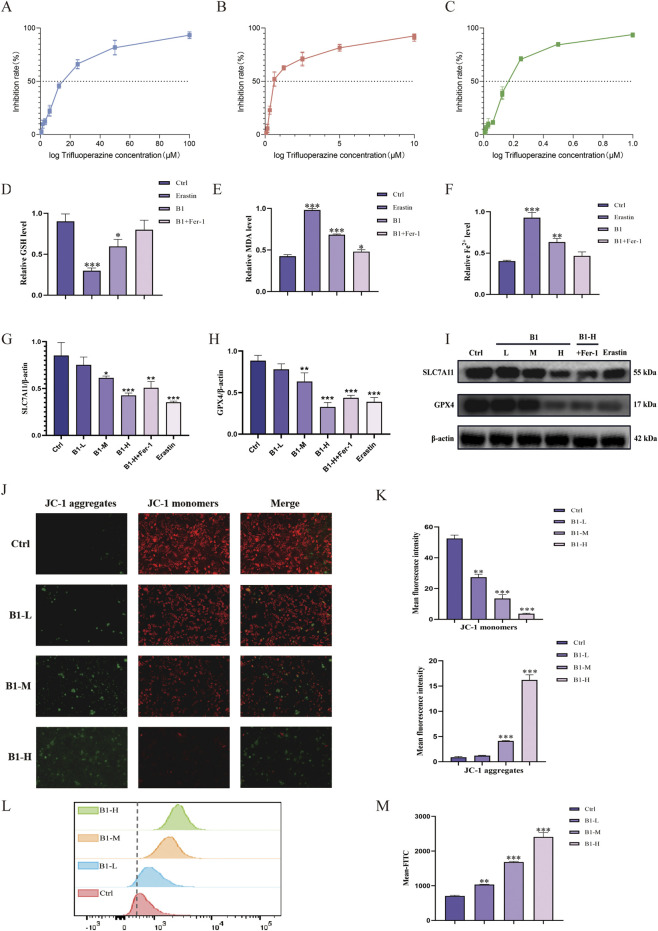
The influence of **B1** on ferropotosis markers: the CCK-8 results of **B1 (A)**, the combination of **B1** and Fer-1 **(B)** and Erastin **(C)**; the effect of **B1** on GSH **(D)**, MDA **(E)** and Fe^2+^ **(F)**, the effect of **B1** on the expression of SLC7A11 and GPX4 **(G–I)**, the effect of **B1** on MMP **(J, K)**, the effect of **B1** on ROS **(L, M)**, n = 3, *p < 0.05, **p < 0.01, ***p < 0.001 vs. “Ctrl” group.

CCK-8 assay measuring HeLa cell viability showed that compound **B1** exhibited stronger inhibitory activity than the ferroptosis inducer erastin (Table 3). Notably, co-treatment with the ferroptosis inhibitor ferrostatin-1 (Fer-1) antagonized **B1**’s cytotoxic effect, leading to reduced potency (Figures 6A–C), suggesting that **B1**’s inhibitory effect is associated with the ferroptosis pathway.

**TABLE 3:** Inhibition rates of compounds on proliferation of HeLa cells.

Compd.	IC_50_/(μmol·L^−1^)
	HeLa
Erastin	17.24 ± 0.53
**B1**	0.22 ± 0.01
Ferrostatin-1 (5 μM) + **B1**	0.60 ± 0.04

The occurrence of ferroptosis in cells can be caused by the decrease of Glutathione (GSH) consumption or synthesis, leading to the accumulation of ROS and lipid peroxidation in cells. Intracellular GSH levels were measured using a GSH assay kit. Compared with the blank group, treatment with 0.22 μmol/L **B1** for 48 h significantly reduced GSH content in HeLa cells (Figure 6D). Cotreatment with Fer-1 (5 μmol/L) increased intracellular GSH levels. These results indicate that **B1** can induce ferroptosis in HeLa cells by depleting GSH.

Malondialdehyde (MDA), as a peroxide product of polyunsaturated fatty acids, can represent the degree of lipid peroxidation damage and is positively correlated with ferroptosis. MDA levels in HeLa cells were detected using an MDA assay kit (Tsikas et al., 2017). Compared with the blank group, treatment with 0.22 μmol/L **B1** for 48 h significantly increased MDA content (Figure 6E). Cotreatment with Fer-1 (5 μmol/L) reduced MDA levels. These results indicate that **B1** can induce ferroptosis in HeLa cells by promoting lipid peroxidation.

Ferroptosis is a form of cell death catalyzed by iron. When the iron metabolism is unbalanced in cells, the Fenton reaction will occur in cells, leading to the occurrence of ferroptosis (Latunde-Data, 2017). Intracellular ferrous iron (Fe^2+^) levels in HeLa cells were measured using an Fe^2+^ assay kit. Resultsshowed that treatment with 0.22 μmol/L **B1** for 48 h significantly increased Fe^2+^ levels (Figure 6F). Cotreatment with Fer-1 (5 μmol/L) reduced Fe^2+^ levels. This indicates that **B1** can induce ferroptosis in HeLa cells by elevating intracellular iron levels.

When redox balance is disrupted within cells, it leads to the accumulation of reactive oxygen species (ROS), which triggers lipid peroxidation in polyunsaturated fatty acids (PUFAs) on organelles and cell membranes, ultimately inducing ferroptosis. To verify whether Compound **B1** induces ferroptosis by elevating intracellular ROS levels, we employed the DCFH-DA probe to quantify intracellular ROS concentrations. Intracellular ROS levels increased gradually in a concentration-dependent manner with increasing **B1** concentration, demonstrating that **B1** induces ROS accumulation and ferroptosis in HeLa cells (Figures 6L,M).

Mitochondria are called the powerhouse of the cell. When oxidative stress occurs, mitochondrial membrane permeability increases and membrane potential decreases, leading to mitochondrial dysfunction and apoptosis (Neitemeier et al., 2017). Mitochondrial membrane potential (MMP) in HeLa cells was assessed using the JC-1 probe and fluorescence microscopy. Compared with the blank group, increasing concentrations of **B1** resulted in a gradual decrease in red fluorescence and an increase in green fluorescence, indicating a progressive loss of MMP in HeLa cells (Figures 6J,K).

SLC7A11 and GPX4 are key proteins in the ferroptosis process. SLC7A11 regulates cysteine transport to influence the synthesis of the antioxidant glutathione, while GPX4 catalyzes the reduction of lipid peroxides through glutathione, protecting cells from oxidative damage. Consequently, inhibition of SLC7A11/GPX4 activity or their expression deficiency promotes ferroptosis. The effect of compound **B1** on SLC7A11 and GPX4 protein expression levels in HeLa cells was determined using Western blot. As shown in, increasing concentrations of **B1** gradually decreased the expression levels of both proteins. Cotreatment with the ferroptosis inhibitor Fer-1 (5 μmol/L) increased the expression levels of both proteins. These results demonstrate that **B1** can induce ferroptosis in HeLa cells by inhibiting the protein expression of SLC7A11 and GPX4, preliminarily suggesting that SLC7A11 and GPX4 are potential targets of **B1** (Figures 6G–I).

The convergence of our transcriptomic and protein-level data elucidates the mechanism of ferroptosis induction via the MDM2-p53 axis. Compound **B1**-mediated p53 stabilization (evidenced by computational and immunoblotting data) transcriptionally represses SLC7A11, as directly observed in our RNA-Seq analysis. This suppression was consistently confirmed at the protein level. The downregulation of SLC7A11 disrupts glutathione synthesis, sensitizing cells to lipid peroxidation and leading to the subsequent inactivation of GPX4, thereby driving the ferroptotic cell death cascade.

## 3 Conclusion

This study has identified **B1**, a novel pyrrolidine-chalcone derivative, as a highly potent and selective anti-cervical cancer agent. Its primary novelty lies in the discovery of a dual mechanism of action that concurrently activates the p53 pathway and induces ferroptosis, a combination rarely reported for chalcone-based compounds. However, a key limitation of this work is that the precise molecular target by which **B1** initiates ferroptosis remains to be elucidated. Future studies will therefore prioritize target identification, validate the *in vivo* efficacy and pharmacokinetic profile of **B1**, and explore the potential synergistic interplay between its pro-apoptotic and ferroptosis-inducing activities. Successful outcomes from these efforts would support the further development of this dual-mechanism strategy.

## 4 Experimental section

### 4.1 Chemical synthesis

#### 4.1.1 Materials

All chemicals used in this study were purchased from commercial suppliers. The starting materials for chemical synthesis were purchased from Bide Pharmatech Ltd. (Shanghai, China; purity >98%). Additional chemical reagents were purchased from Tianjin Xinbote Chemical Co., Ltd. (Tianjin, China), all of analytical grade.

#### 4.1.2 Synthesis methods

Synthesis of compound 3 and 6: Fluorobenzaldehyde or fluoroacetophenone (4 mmol) and pyrrolidine (10 mmol) were added to a 100-mL three-necked flask and dissolved in N,N-Dinethylformamide (DMF), and an appropriate amount of triethylamine (TEA) was added. The mixture was heated with stirring under reflux, and the reaction was monitored by TLC throughout. Upon completion, the mixture was poured into a beaker. The pH was adjusted to one to three with dilute hydrochloric acid, extracted using a separatory funnel, then adjusted to pH 9–10 with potassium carbonate, and extracted three times with ethyl acetate. The combined organic extracts were concentrated under reduced pressure to obtain compound 3 or 6.

Synthesis of Target Chalcone Derivatives (e.g., **A1**): A derivative of acetophenone (10 mmol) and 0.5 g of solid potassium hydroxide were added to a 100 mL three-necked flask. Anhydrous ethanol (10 mL) was added, and the mixture was dissolved with ultrasonication assistance. Compound 3 (8 mmol) was then added, and the reaction mixture was stirred at room temperature for 0.5–2 h, monitored by TLC throughout the reaction. After completion, the mixture was filtered under vacuum to collect the solid, which was washed three times with cold anhydrous ethanol to yield the crude product. The compound was purified by recrystallization from hot anhydrous ethanol, followed by further purification via silica gel column chromatography to yield compound **A1**. The synthesis of other target chalcone derivatives followed a similar procedure.

#### 4.1.3 Structural characterization

Melting points were determined using a micro melting point apparatus. NMR spectra were recorded at the Xinjiang Technical Institute of Physics and Chemistry, Chinese Academy of Sciences (Bruker AVANCE NEO-600). ^1^H NMR spectra were recorded at 400 MHz and ^13^C NMR spectra at 101 MHz, using CDCl_3_ as the solvent. Mass spectra were recorded at Xinjiang University using a mass spectrometer (Thermo Scientific LTQ Orbitrap XL). Characterization data of key intermediates and all final compounds, as well as the synthetic route, are provided in the Supplementary Material S1.

### 4.2 Biological evaluation

#### 4.2.1 Cell culture

HeLa, SiHa, C-33A, and H8 cell lines were cultured in basal medium at 37 °C in a humidified incubator with 5% CO_2_. All cell lines were purchased from Procell Life Science and Technology Co., Ltd. (Wuhan, China).

#### 4.2.2 Antiproliferative activity

Cells were seeded into 96-well plates at a density of 2.5–6 × 10^3^ cells per well and incubated for 24 h. They were then treated with a series of eight concentrations of the test compounds (e.g., from 100 to 0.78125 μM or from 2 to 0.015625 μM, prepared by twofold serial dilution) for 48 h. After treatment, the medium was removed and replaced with 100 μL of 10% CCK-8 solution (diluted in fresh medium). Following incubation in the dark for 1–2 h, absorbance at 450 nm was measured using a microplate reader. The half-maximal inhibitory concentration (IC_50_) was calculated using SPSS software. Each concentration was tested in six replicate wells, and the entire experiment was performed in three independent biological replicates.

#### 4.2.3 Cell apoptosis

HeLa cells were cultured in six-well plates until 80% confluency and then divided into five treatment groups: a negative control and three groups treated with compound **B1** at concentrations of 0.11, 0.22, and 0.44 μM (labeled in figures as **B1**-L, **B1**-M, and **B1**-H, respectively). After 48 h of treatment, cells were collected, washed twice with PBS, and resuspended in 100 μL of binding buffer. According to the kit instructions (BD Biosciences Pharmingen, San Diego, CA, United States of America), 5 μL of Annexin V/FITC and 5 μL of PI were added to the suspension. Cells were incubated in the dark for 15 min, followed by the addition of 400 μL of binding buffer. Cell apoptosis was analyzed by flow cytometry (BD Biosciences Pharmingen, San Diego, CA, United States of America). The apoptosis assay was performed in three independent experiments. The same group designations (**B1**-L, **B1**-M, **B1**-H) apply throughout the study where applicable.

#### 4.2.4 Cell cycle arresting effect

HeLa cells were cultured in six-well plates until 80% confluency and treated with different concentrations of **B1** for 24 h at 37 °C and 5% CO_2_. Cells were then collected, washed twice with PBS, and fixed in 3 mL of 75% ethanol at −20 °C for 24 h. After ethanol removal, cells were stained with 400 μL of PI/RNase staining solution (BD Biosciences Pharmingen) for 20 min. Cell cycle distribution was analyzed by flow cytometry (BD Biosciences Pharmingen, San Diego, CA, United States of America). The cell cycle assay was performed in three independent experiments.

#### 4.2.5 Migration effect

Lines were drawn on the back of six-well plates for marking. HeLa cells were cultured in the plates until 80% confluency was reached. A scratch wound was created with a sterile pipette tip, and cells were then treated with different concentrations of **B1**. The plates were incubated at 37 °C and 5% CO_2_. The width of the scratch wounds was observed and photographed at 0, 24, and 48 h. The migration assay was performed in three independent experiments.

#### 4.2.6 Invasion effect

Transwell inserts were placed in a 24-well plate. Matrigel® (50 μL; diluted 1:8 in serum-free DMEM) was added to the upper chamber of each insert and allowed to solidify at 37 °C for 1 h. Cell suspensions (6 × 10^4^ cells in serum-free medium) were added to the upper chamber. The lower chamber was filled with 600 μL of medium containing 15% FBS and **B1**. The plates were incubated at 37 °C for 48 h. Subsequently, the medium was removed. Cells on the upper surface of the membrane were gently wiped off. Invaded cells on the lower surface were fixed with 4% paraformaldehyde for 20 min, washed, stained with 0.1% crystal violet for 20 min, washed again, air-dried, and observed under an inverted microscope, and the images recorded. The invasion assay was performed in three independent experiments.

#### 4.2.7 Immunofluorescence

Glass coverslips (0.8-cm diameter) were placed flat and nonoverlapping in six-well plates. A suspension of digested HeLa cells was added, gently mixed, and placed in a constant temperature incubator. Cells were cultured until 70% confluency and treated with different concentrations of **B1** for 24 h. When cell density reached 90%–95% under microscopic observation, the medium was removed. Cells were fixed with 4% paraformaldehyde at room temperature for 15 min and washed three times with PBS. Permeabilization was performed with 0.1% saponin for 10 min. Nonspecific binding was blocked using 10% goat serum in a humidified chamber at room temperature for 30 min. Primary antibodies, diluted according to the manufacturer’s instructions, were added to the coverslips and incubated in the dark for 1 h in a humidified chamber. After three PBS washes, appropriately diluted secondary antibodies were added to the coverslips and incubated in the dark at room temperature for 1 h. Cells were counterstained with DAPI for 10 min and washed three times with PBS. Coverslips were mounted onto glass slides using an anti-fade mounting medium and allowed to set in a dark, flat place for 30 min. Images were acquired using a Nikon AX/AXR confocal microscope. The immunofluorescence assay was performed in three independent experiments.

#### 4.2.8 Western blotting assay

HeLa cells were cultured in six-well plates to 80% confluency and then divided into five groups for treatment: a negative control group, a positive control group (0.22 μM Nutlin-3a), and three groups treated with compound B1 at concentrations of 0.11, 0.22 and 0.44 μM, which are labeled in the figures as **B1**-L, **B1**-M, and **B1**-H, respectively. Cells were treated for 24 h before subsequent analysis. Cells were collected using RIPA lysis buffer containing protease and phosphatase inhibitors and lysed on ice for 30 min. Lysates were centrifuged at 12,000 rpm for 20 min at 4 °C. The protein concentration in the supernatant was determined using a BCA Protein Assay Kit (Thermo Fisher Scientific, Rockford, IL, United States of America). Total protein was separated by SDS-PAGE and transferred to PVDF membranes. Membranes were blocked with 5% nonfat milk in TBST at room temperature for 2 h. Subsequently, membranes were incubated with primary antibodies overnight at 4 °C. The primary antibodies used were: anti-MDM2 (1:1,000, bsm, Cat# 62661R), anti-p53 (1:800, Servicebio, Cat# GB15627), anti-GPX4 (1:1,000, Abmart, Cat# T56959), and anti-SLC7A11 (1:1,000, Abmart, Cat# T57046). After primary antibody incubation, membranes were washed three times with TBST, incubated with secondary antibody (1:10,000, Proteintech, Cat# SA00001-2) for 1 h at room temperature, followed by three washes with TBST. Protein bands were visualized using chemiluminescence with an imaging system (Image Quant LAS 500) and an ECL Prime Western blotting Detection Kit. The Western blotting assay was performed in three independent experiments.

#### 4.2.9 Transcriptome sequencing analysis

Transcriptome sequencing was performed on HeLa cells treated with 0.25 μM of compound **B1** for 24 h. Following collection, cells were flash-frozen and submitted to MetWare Biotechnology Co., Ltd. (Wuhan, China) for analysis. Strand specific mRNA-seq libraries were constructed via poly(A) selection, RNA fragmentation, cDNA synthesis, and PCR amplification. Sequencing was carried out on either an Illumina NovaSeq 6000 or a DNBSEQ-T7 platform (PE150). The data analysis pipeline included the following steps: quality control and adapter trimming of raw reads using fastp (v0.23.2); alignment to the reference genome with HISAT2 (v2.2.1); transcript assembly and expression quantification using StringTie (v2.2.1); and identification of differentially expressed genes with DESeq2 (v1.40.0), applying a cut-off of |log_2_ (fold change)| ≥1 and an adjusted p-value (padj) <0.05.

#### 4.2.10 MDA, Fe^2+^, and GSH assays

HeLa cells were cultured in 6-cm cell culture dishes until 80% confluency and then divided into four treatment groups: a negative control (Ctrl), 0.22 μM **B1** alone, 0.22 μM **B1** co-treated with 5 μM Ferrostatin-1 (Fer-1), and 0.22 μM erastin (a ferroptosis inducer) as a positive control. After 24 h of treatment, cells were collected and washed twice with PBS for subsequent analysis. This group designation (Ctrl, **B1**, **B1**+Fer-1, Erastin) is consistently applied in all relevant experiments. Subsequent procedures were performed strictly according to the manufacturer’s instructions of the respective kits: MDA assay kit (Servicebio, Cat# GM1134), Fe^2+^ colorimetric assay kit (Servicebio, Cat# GM3052), and reduced GSH assay kit (Servicebio, Cat# GM1139).

#### 4.2.11 Molecular docking study

The 3D structures of the small molecules were sketched using molecular docking software and saved in .mol2 file format. High-resolution crystal structures of the protein targets were retrieved from the RCSB PDB database (http://www.rcsb.org/) and used as molecular docking receptors. Using AutoDock Vina 1.5.6 software, both the protein and small molecule ligand structures were prepared: hydrogen atoms were added to the proteins and water molecules were removed. For the ligands, hydrogen atoms were added to the small molecule ligand and its torsional degrees of freedom were assigned. The coordinates of the docking box were subsequently defined. By comparing the docking scores (affinity values) of the results, the optimal conformation for the molecular simulation was ultimately identified. Visualization of the docking results was performed using PyMOL.

#### 4.2.12 MD simulation

This study employed Gromacs 2022 for MD simulations. Force field parameters for the system were generated using GROMACS’ pdb2gmx tool and the AutoFF web server. During the simulations, the CHARMM36 force field was applied to the receptor protein (Jo et al., 2008), while ligand parameters were assigned using the CGenFF force field (https://cgenff.com/) (Vanommeslaeghe et al., 2012). The system was solvated in a cubic TIP3P water box extending 1 nm beyond the solute, filled with TIP3P water molecules (Mark and Nilsson, 2001). Ions were added using the gmx genion tool to achieve overall system charge neutrality. Long-range electrostatic interactions were calculated using the particle mesh Ewald (PME) method, with a cutoff distance of 1.0 nm. All bond constraints were implemented using the SHAKE algorithm. The leap-frog Verlet algorithm was employed for integration, with a time step of 1 fs. Prior to MD production runs, the system underwent energy minimization, which consisted of 3,000 steps using the steepest descent algorithm, followed by 2,000 steps using the conjugate gradient algorithm. Production simulations were performed under NPT ensemble conditions at a temperature of 310 K and constant pressure, with a total simulation time of 100 ns.

#### 4.2.13 In silico ADMET prediction

The prediction of absorption, distribution, metabolism, excretion, and toxicity (ADMET) properties for compound **B1** was performed *in silico* using the ADMETlab 2.0 online platform (https://admetmesh.scbdd.com/). The canonical SMILES notation of **B1** was used as the input. The platform employs pre-trained models based on molecular descriptors and machine learning algorithms, which have been developed and validated against large-scale experimental datasets. All parameters were calculated using the platform’s default settings.

#### 4.2.14 Statistical analysis

IC_50_ values were derived by nonlinear regression (SPSS 26). All data are presented as mean ± SD from at least three independent biological replicates (n ≥ 3). Differences were assessed by Student’s t-test (two groups) or one-way ANOVA with Tukey’s *post hoc* test (multiple groups). The specific n value for each experiment is stated in its figure legend. Statistical significance was set at p < 0.05.

## Data Availability

The raw sequencing data have been deposited in the NCBI GEO database under the accession number GSE317187, available at: https://www.ncbi.nlm.nih.gov/search/all/?term=GSE317187.
