# Supplementary material for: A novel pyrrolidine-chalcone derivative exhibits synergistic anti-cervical cancer activity by dual-targeting MDM2-p53 axis and ferroptosis pathway
Source: Front Pharmacol. 2026 Feb 6;17:1715712. doi: 10.3389/fphar.2026.1715712 (PMC12921483; doi:10.3389/fphar.2026.1715712)
Supplement: Supplementary file 1 [file DataSheet1.docx]

4-(pyrrolidin-1-yl)benzaldehyde(**Compound 3**). Grey powder, Yiled 81.5 %, m.p.: 80.8～81.6 ℃. ^1^H NMR (400 MHz, CDCl_3_) *δ* 9.70 (s, 1H, Ar-H), 7.76～7.67 (m, 2H, Ar-H), 6.55 (d, *J* = 8.7 Hz, 1H, Ar-H), 6.53～6.46 (m, 1H, O=C-H), 3.37～3.32 (m, 4H, -CH_2_), 2.10～1.97 (m, 4H, -CH_2_). ^13^C NMR (101 MHz, CDCl_3_) *δ* 190.19, 101.94, 130.66, 124.82, 111.18, 110.64, 47.63, 47.55, 25.93, 25.40.

4-4-(pyrrolidin-1-yl)acetophenone(**Compound 6**). Yellow powder, Yiled 82.3 %, m.p.: 129.3～129.8 ℃. ^1^H NMR (400 MHz, CDCl_3_) *δ* 8.14～7.61 (m, 2H, Ar-H), 6.64～6.42 (m, 2H, Ar-H), 3.52～3.19 (m, 4H, -CH_2_), 2.51(s, 3H, -CH_3_), 2.22～1.83 (m, 4H, -CH_2_). ^13^C NMR (101 MHz, CDCl_3_) *δ* 196.29, 150.84, 130.68, 125.14, 110.85, 47.72, 25.94, 25.41.

(*E*)-1-(2-methoxyphenyl)-3-(4-(pyrrolidin-1-yl)phenyl)prop-2-en-1-one (**A1**). Yellow crystals, Yiled 68.4 %. m.p.: 126.8～127.2 ℃. HRMS (ESI) *m/z* calcd for C_20_H_21_NO_2_^+^ (M + H^+^) 308.1651, found 308.1640. ^1^H NMR (400 MHz, CDCl_3_) *δ* 7.56～7.48 (m, 2H), 7.48 (d, *J* = 15.7 Hz, 1H, H*_β_*), 7.40～7.37 (m, 2H, Ar-H), 7.08 (d, *J* = 15.7 Hz, 1H, H*_α_*), 7.04～6.93 (m, 2H, Ar-H), 6.56～6.48 (m, 2H, Ar-H), 3.86 (s, 3H, -CH_3_), 3.37～3.28 (m, 4H, -CH_2_), 2.07～1.96 (m, 4H, -CH_2_). ^13^C NMR (101 MHz, CDCl_3_) *δ* 193.56, 145.55, 131.81, 130.49, 130.21, 129.90, 122.08, 121.68, 120.52, 111.65, 111.54, 55.76, 55.73, 47.53, 25.43.

(*E*)-1-(2,4-dimethoxyphenyl)-3-(4-(pyrrolidin-1-yl)phenyl)prop-2-en-1-one (**A2**). Golden yellow crystals, Yield 67.3 %, m.p.: 125.3～126.0 ℃, HRMS (ESI) *m/z* calcd for C_21_H_23_NO_3_^+^ (M + H^+^) 338.1757, found 338.1747. ^1^H NMR (400 MHz, CDCl_3_) *δ* 7.68 (d, *J* = 8.5 Hz, 1H, Ar-H), 7.62 (d, *J* = 15.7 Hz, 1H, H*_β_*), 7.50～7.46 (m, 2H, Ar-H), 7.28 (s, 1H, Ar-H), 6.60 (d, *J* = 8.4 Hz, 2H, Ar-H), 6.54 (d, *J* =15.7 Hz, 1H, H*_α_*), 6.48 (d, *J* = 2.3 Hz, 1H, Ar-H), 3.86 (d, *J* = 8.8 Hz, 6H, -CH_3_), 3.44～3.29 (m, 4H, -CH_2_), 2.06～1.95 (m, 4H, -CH_2_). ^13^C NMR (101 MHz, CDCl_3_) *δ* 191.02, 172.24, 163.45, 159.92, 143.77, 132.40, 130.30, 123.03, 122.13, 112.17, 104.84, 98.73, 86.56, 55.72, 55.49, 48.11, 25.39.

(*E*)-1-(2,6-dimethoxyphenyl)-3-(4-(pyrrolidin-1-yl)phenyl)prop-2-en-1-one (**A3**). Golden yellow crystals, Yield 71.8 %, m.p.: 124.3～124.8 ℃, HRMS (ESI) m/z calcd for C_21_H_23_NO_3_^+^ (M + H^+^) 338.1757, found 338.1750. ^1^H NMR (400 MHz, CDCl_3_) *δ* 7.85～7.72 (m, 2H, Ar-H), 7.68 (d, *J* = 15.1 Hz, 1H, H*_β_*), 7.56～7.47 (m, 2H, Ar-H), 7.36～7.34 (m, 1H, Ar-H), 7.26 (d, *J* = 15.1 Hz, 1H, H*_α_*), 6.59～6.49 (m, 2H, Ar-H), 3.41～3.26 (m, 4H, -CH_2_), 2.42 (s, 3H, -CH_3_), 2.08～1.90 (m, 4H, -CH_2_), 1.64(s, 3H, -CH_3_). ^13^C NMR (101 MHz, CDCl_3_) *δ* 190.56, 149.58, 146.03, 139.22, 138.17, 132.80, 130.59, 128.83, 128.24, 125.45, 122.08, 116.44, 111.69, 58.45, 47.55, 25.43, 21.40.

(*E*)-3-(4-(pyrrolidin-1-yl)phenyl)-1-(2,3,4-trimethoxyphenyl)prop-2-en-1-one (**A4**). Yellow crystals, Yield 69.6 %, m.p.: 127.1～127.5 ℃, HRMS (ESI) *m/z* calcd for C_22_H_25_NO_4_^+^ (M + H^+^) 368.1863, found 368.1951. ^1^H NMR (400 MHz, CDCl_3_) *δ* 7.62 (d, *J* = 15.6 Hz, 1H, H*_β_*), 7.53～7.44 (m, 2H, Ar-H), 7.40 (d, *J* = 8.7 Hz, 1H, Ar-H), 7.22 (d, *J* = 15.6 Hz, 1H, H*_α_*), 6.72 (d, *J* = 8.8 Hz, 1H, Ar-H), 6.59～6.41 (m, 2H, Ar-H), 3.93～3.85 (m, 9H, -CH_3_), 3.38～3.29 (m, 4H, -CH_2_), 2.07～1.94 (m, 4H, -CH_2_). ^13^C NMR (101 MHz, CDCl_3_) *δ* 191.31, 156.22, 149.44, 145.08, 130.48, 127.71, 125.36, 122.17, 120.98, 111.68, 107.13, 62.06, 61.05, 56.07, 47.53, 25.43.

(*E*)-3-(4-(pyrrolidin-1-yl)phenyl)-1-(2-(trifluoromethyl)phenyl)prop-2-en-1-one (**A5**) . Yellow crystals, Yield 79.7 %, m.p.: 120.7～121.5 ℃, HRMS (ESI) *m/z* calcd for C_20_H_18_F_3_NO^+^ (M + H^+^) 346.1419, found 346.1409. ^1^H NMR (400 MHz, CDCl_3_) *δ* 7.72 (d, *J* = 7.5 Hz, 1H, Ar-H), 7.63～7.49 (m, 2H, Ar-H), 7.43 (d, *J* = 7.4 Hz, 1H, Ar-H), 7.41～7.35 (m, 2H, Ar-H), 7.19 (d, *J* = 16.0 Hz, 1H, H*_β_*), 6.81 (d, *J* = 15.9 Hz, 1H, H*_α_*), 6.52 (d, *J* = 8.7 Hz, 2H, Ar-H), 3.37～3.30 (m, 4H, -CH_2_), 2.07～1.96 (m, 4H, -CH_2_). ^13^C NMR (101 MHz, CDCl_3_) *δ* 194.87, 149.91, 149.30, 131.41, 130.78, 129.21, 128.23, 126.53, 121.15, 111.73, 47.55, 25.40.

(*E*)-3-(4-(pyrrolidin-1-yl)phenyl)-1-(3-(trifluoromethyl)phenyl)prop-2-en-1-one (**A6**). Golden Yellow crystals, Yield 71.3 %, m.p.: 116.7~117.3 ℃, HRMS (ESI) *m/z* calcd for C_20_H_18_F_3_NO^+^ (M + H^+^) 346.1419, found 346.1412. ^1^H NMR (400 MHz, CDCl_3_) *δ* 8.23 (s, 1H), 8.16 (d, *J* = 15.8 Hz, 1H, H*_β_*), 7.87～7.75 (m, 2H, Ar-H), 7.60 (d, *J* = 15.8 Hz, 1H, H*_α_*), 7.58～7.51 (m, 2H, Ar-H), 7.31～7.22 (m, 1H, Ar-H), 6.58 (dd, *J* = 8.9, 2.2 Hz, 2H, Ar-H), 3.41～3.33 (m, 4H, -CH_2_), 2.10～1.99 (m, 4H, -CH_2_). ^13^C NMR (101 MHz, CDCl_3_) *δ* 189.23, 150.15, 147.39, 139.89, 139.82, 131.38, 130.93, 129.00, 128.36, 125.06, 125.03, 121.57, 115.15, 111.76, 47.57, 25.42.

(*E*)-3-(4-(pyrrolidin-1-yl)phenyl)-1-(m-tolyl)prop-2-en-1-one (**A7**). Golden Yellow crystals, Yield 73.6 %, m.p.: 128.8～129.7 ℃, HRMS (ESI) *m/z* calcd for C_20_H_21_NO^+^ (M + H^+^) 291.1702, found 292.1694. ^1^H NMR (400 MHz, CDCl_3_) *δ* 8.06 (d, *J* = 8.1 Hz, 2H), 7.79 (d, *J* = 15.4 Hz, 1H, H*_β_*), 7.72 (d, *J* = 8.1 Hz, 2H, Ar-H), 7.57～7.49 (m, 2H, Ar-H), 7.24 (d, *J* = 15.4 Hz, 1H, H*_α_*), 6.59～6.51 (m, 2H, Ar-H), 3.40～3.31 (m, 4H, -CH_2_), 2.09～1.97 (m, 4H, -CH_2_), 1.24 (s, 3H, -CH_3_). ^13^C NMR (101 MHz, CDCl_3_) *δ* 189.63, 149.91, 147.47, 142.21, 133.47, 130.91, 128.50, 125.44, 125.40, 122.12, 121.67, 115.61, 111.78, 47.58, 25.43.

(*E*)-3-(4-(pyrrolidin-1-yl)phenyl)-1-(4-(trifluoromethyl)phenyl)prop-2-en-1-one (**A8**). Golden Yellow powder, Yield 70.2 %, m.p.: 171.0～171.6 ℃, HRMS (ESI) *m/z* calcd for C_20_H_18_F_3_NO^+^ (M + H^+^) 346.1419, found 346.1412. ^1^H NMR (400 MHz, CDCl_3_) *δ* 8.06 (d, *J* = 8.1 Hz, 2H, Ar-H), 7.79 (d, *J* = 15.4 Hz, 1H, H*_β_*), 7.72 (d, *J* = 8.1 Hz, 2H, Ar-H), 7.57～7.49 (m, 2H, Ar-H), 7.24 (d, *J* = 15.4 Hz, 1H, H*_α_*), 6.59～6.51 (m, 2H, Ar-H), 3.40～3.31 (m, 4H, -CH_2_), 2.09～1.97 (m, 4H, -CH_2_). ^13^C NMR (101 MHz, CDCl_3_) *δ* 190.88, 149.58, 146.03, 139.22, 138.17, 132.80, 130.59, 128.83, 125.45, 122.08, 116.44, 111.69, 58.45, 47.55, 25.43, 21.40.

(*E*)-3-(4-(pyrrolidin-1-yl)phenyl)-1-(2,3,4-trichlorophenyl)prop-2-en-1-one (**A9**). Golden Yellow crystals, Yield 73.4 %, m.p.: 150.5～101.2 ℃, HRMS (ESI) *m/z* calcd for C_19_H_16_Cl_3_NO^+^ (M + H^+^) 382.0347, found 382.0332. ^1^H NMR (400 MHz, CDCl_3_) *δ* 7.48～7.39 (m, 3H, Ar-H), 7.30 (d, *J* = 15.9 Hz, 1H, H*_β_*),7.22(d, *J* = 8.1 Hz, 1H, Ar-H), 6.79 (d, *J* = 15.8 Hz, 1H, H*_α_*), 6.55 (t, *J* = 8.9 Hz, 2H, Ar-H), 3.36～3.31 (m, 4H, -CH_2_), 2.85～2.79 (m, 4H, -CH_2_). ^13^C NMR (101 MHz, CDCl_3_) *δ* 192.26, 150.10, 149.14, 135.06, 131.03, 128.37, 126.81, 121.10, 119.96, 111.82, 47.58, 25.41.

(*E*)-1-(5-fluoro-2-methoxyphenyl)-3-(4-(pyrrolidin-1-yl)phenyl)prop-2-en-1-one (**B1**). Orange crystals, Yield 75.3 %, m.p.: 122.3～122.5 ℃, HRMS (ESI) *m/z* calcd for C_20_H_20_FNO_2_^+^ (M + H^+^) 326.1557, found 326.1547. ^1^H NMR (400 MHz, CDCl_3_) *δ* 7.54 (d, *J* = 15.7 Hz, 1H, H*_β_*), 7.49～7.42 (m, 2H, Ar-H), 7.27 (d, *J* = 15.7 Hz, 1H, H*_α_*), 7.15～7.03 (m, 2H, Ar-H), 6.91 (dd, *J* = 9.0, 4.1 Hz, 1H, Ar-H), 6.55～6.49 (m, 2H, Ar-H), 3.84 (s, 3H, -CH_3_), 3.34～3.28 (m, 4H, -CH_2_), 2.07～1.96 (m, 4H, -CH_2_). ^13^C NMR (101 MHz, CDCl_3_) *δ* 191.84, 149.65, 146.27, 146.20, 130.65, 130.61, 121.86, 120.90, 118.04, 117.81, 116.57, 116.33, 112.99, 112.91, 111.77, 56.50, 47.60, 47.50, 25.47, 25.43.

(*E*)-1-(5-bromo-2-methoxyphenyl)-3-(4-(pyrrolidin-1-yl)phenyl)prop-2-en-1-one (**B2**). Orange crystals, Yield 77.6 %, m.p.: 122.5～122.8 ℃, HRMS (ESI) *m/z* calcd for C_20_H_20_BrNO_2_^+^ (M + H^+^) 386.0756, found 386.0748. ^1^H NMR (400 MHz, CDCl_3_) *δ* 7.52 (d, *J* = 15.7 Hz, 1H, H*_β_*), 7.52～7.42 (m, 3H, Ar-H), 7.35 (d, *J* = 8.8 Hz, 1H, Ar-H), 7.03 (d, *J* = 15.7 Hz, 1H, H*_α_*), 6.90 (d, *J* = 8.8 Hz, 1H, Ar-H), 6.52 (d, *J* = 8.7 Hz, 2H, Ar-H), 3.84 (s, 3H, -CH_3_), 3.30～3.27 (m, 4H, -CH_2_), 2.06～2.01 (m, 4H, -CH_2_). ^13^C NMR (101 MHz, CDCl_3_) *δ* 191.86, 156.17, 149.67, 146.40, 131.29, 130.69, 129.59, 125.72, 121.80, 120.91, 112.98, 56.06, 47.61, 47.50, 25.43.

(*E*)-1-(5-chloro-2-methoxyphenyl)-3-(4-(pyrrolidin-1-yl)phenyl)prop-2-en-1-one (**B3**). Orange crystals, Yield 78.5 %, m.p.: 127.1～127.5 ℃, HRMS (ESI) *m/z* calcd for C_20_H_20_ClNO_2_^+^ (M + H^+^) 342.1262, found 341.1174. ^1^H NMR (400 MHz, CDCl_3_) *δ* 7.63 (d, *J* = 15.7 Hz, 1H, H*_β_*), 7.60～7.41 (m, 4H, Ar-H), 7.02 (d, *J* = 15.7 Hz, 1H, H*_α_*), 6.85 (d, *J* = 8.8 Hz, 1H, Ar-H), 6.57～6.49 (m, 2H, Ar-H), 3.84 (s, 3H, -CH_3_), 3.39～3.30 (m, 4H, -CH_2_), 2.08～1.97 (m, 4H, -CH_2_). ^13^C NMR (101 MHz, CDCl_3_) *δ* 191.81, 156.64, 149.67, 146.39, 134.30, 132.44, 130.64, 121.81, 120.90, 113.41, 111.67, 55.98, 47.56, 25.46, 25.44.

(*E*)-1-(2-methoxyphenyl)-2-methyl-3-(4-(pyrrolidin-1-yl)phenyl)prop-2-en-1-one (**B4**). Yellow crystals, Yield 67.1 %, m.p.: 125.7～125.9 ℃, HRMS (ESI) *m/z* calcd for C_21_H_23_NO_2_^+^ (M + H^+^) 322.1729, found 322.1798. ^1^H NMR (400 MHz, CDCl_3_) *δ* 7.61 (d, *J* = 15.9 Hz, 1H, H*_β_*), 7.45～7.36 (m, 2H, Ar-H), 7.28～7.17 (m, 2H, Ar-H), 6.97～6.84 (m, 2H, Ar-H), 6.58 (d, *J* = 15.9 Hz, 1H, H*_α_*), 6.54～6.47 (m, 2H, Ar-H), 3.90 (s, 3H, -CH_3_), 3.82 (s, 3H, -CH_3_), 3.37～3.29 (m, 4H, -CH_2_), 2.02～1.97 (m, 4H, -CH_2_). ^13^C NMR (101 MHz, CDCl_3_) *δ* 197.92, 149.41, 144.09, 131.05, 130.36, 128.20, 128.10, 121.67, 120.64, 119.94, 111.73, 110.52, 55.33, 47.60, 42.19, 25.43.

(*E*)-3-(4-(pyrrolidin-1-yl)phenyl)-1-(2-(trifluoromethoxy)phenyl)prop-2-en-1-one (**B5**). Yellow crystals, Yield 61.6 %, m.p.: 101.2～101.9 ℃, HRMS (ESI) *m/z* calcd for C_20_H_18_F_3_NO_2_^+^ (M + H^+^) 362.1369, found 362.1356. ^1^H NMR (400 MHz, CDCl_3_) *δ* 7.63 (dd, *J* = 15.7 Hz, 1H, H*_β_*), 7.55～7.47 (m, 1H, Ar-H), 7.52-7.42 (m, 3H, Ar-H), 7.42～7.29 (m, 2H, Ar-H), 6.97 (d, *J* = 15.7 Hz, 1H, H*_α_*), 6.59～6.51 (m, 2H, Ar-H), 3.40～3.30 (m, 4H, -CH_2_), 2.09～1.97 (m, 4H, -CH_2_). ^13^C NMR (101 MHz, CDCl_3_) *δ* 149.80, 147.43, 131.55, 130.79, 130.21, 126.90, 121.62, 121.26, 121.24, 120.27, 111.80, 47.60, 25.42.

(*E*)-3-(4-(pyrrolidin-1-yl)phenyl)-1-(3-(trifluoromethoxy)phenyl)prop-2-en-1-one (**B6**). Orange crystals, Yield 78.7 %, m.p.: 113.7～114.6 ℃, HRMS (ESI) *m/z* calcd for C_20_H_18_F_3_NO_2_^+^ (M + H^+^) 362.1369, found 362.1360. ^1^H NMR (400 MHz, CDCl_3_) *δ* 7.92 (d, *J* = 7.7 Hz, 1H, Ar-H), 7.85～7.79 (m, 2H, Ar-H), 7.55 (s, 1H, Ar-H), 7.55～7.48 (m, 2H, Ar-H), 7.39 (dd, *J* = 15.3 Hz, 1H, H*_β_*), 7.24 (d, *J* = 15.3 Hz, 1H, H*_α_*), 6.57 (d, *J* = 8.5 Hz, 2H, Ar-H), 3.37～3.31(m, 4H, -CH_2_), 2.08～2.00 (m, 4H, -CH_2_). ^13^C NMR (101 MHz, CDCl_3_) *δ* 188.85, 149.81, 149.40, 147.19, 141.19, 130.85, 129.87, 126.49, 124.23, 121.91, 121.81, 120.75, 115.44, 111.89, 47.68, 25.42.

(*E*)-3-(4-(pyrrolidin-1-yl)phenyl)-1-(4-(trifluoromethoxy)phenyl)prop-2-en-1-one (**B7**). Orange crystals, Yield 55.8 %, m.p.: 167.1～167.8 ℃, HRMS (ESI) *m/z* calcd for C_20_H_18_F_3_NO_2_^+^ (M + H^+^) 362.1369, found 362.1360. ^1^H NMR (400 MHz, CDCl_3_) *δ* 7.92 (d, *J* = 7.7 Hz, 1H, Ar-H), 7.85～7.79 (m, 2H, Ar-H), 7.55 (s, 1H, Ar-H), 7.55～7.48 (m, 2H, Ar-H), 7.39 (dd, *J* = 15.3 Hz, 1H, H*_β_*), 7.24 (d, *J* = 15.3 Hz, 1H, H*_α_*), 6.57 (d, *J* = 8.5 Hz, 2H, Ar-H), 3.37～3.32 (m, 4H, -CH_2_), 2.08～2.00 (m, 4H, -CH_2_). ^13^C NMR (101 MHz, CDCl_3_) *δ* 188.85, 149.81, 147.19, 141.19, 130.85, 129.87, 126.49, 124.23, 121.91, 120.75, 115.44, 111.89, 47.68, 25.42.

(*E*)-1-(2,5-dimethoxyphenyl)-3-(4-(pyrrolidin-1-yl)phenyl)prop-2-en-1-one (**C1**). Yellow crystals, Yield 56.6 %, m.p.: 142.6～143.2 ℃, HRMS (ESI) *m/z* calcd for C_21_H_23_NO_3_^+^ (M + H^+^) 338.1757, found 338.1748. ^1^H NMR (400 MHz, CDCl_3_) *δ* 7.56 (d, *J* = 15.7 Hz, 1H, H*_β_*), 7.46 (d, *J* = 8.5 Hz, 2H, Ar-H), 7.20～7.08 (m, 2H, Ar-H), 7.01～6.87 (m, 3H, Ar-H), 6.53 (dd, *J* = 15.7 Hz, 1H, H*_α_*), 3.81 (dd, *J* = 12.8, 0.9 Hz, 6H, -CH_3_), 3.33～3.38 (m, 4H, -CH_2_), 2.09～1.95 (m, 4H, -CH_2_). ^13^C NMR (101 MHz, CDCl_3_) *δ* 192.92, 153.53, 152.05, 149.50, 145.59, 130.78, 122.11, 121.41, 117.91, 114.33, 113.39, 111.68, 56.61, 55.84, 47.54, 25.43.

(*E*)-1-(2,5-difluorophenyl)-3-(4-(pyrrolidin-1-yl)phenyl)prop-2-en-1-one (**C2**). Yellow crystals, Yield 46.9 %, m.p.: 144.0～144.8 ℃, HRMS (ESI) *m/z* calcd for C_19_H_17_F_2_NO^+^ (M + H^+^) 314.1357, found 314.1363. ^1^H NMR (400 MHz, CDCl_3_) *δ* 7.71 (dd, *J* = 15.5 Hz, 1H, H*_β_*), 7.51 (s, 1H, Ar-H), 7.49 (s, 1H, Ar-H), 7.51～7.42 (m, 1H, Ar-H), 7.20(dd, *J* = 15.5 Hz, 1H, H*_α_*), 7.16～7.05 (m, 3H, Ar-H), 6.57～6.49 (m, 2H, Ar-H), 3.35～3.30 (m, 4H, -CH_2_), 2.09～1.97 (m, 4H, -CH_2_). ^13^C NMR (101 MHz, CDCl_3_) *δ* 187.35, 149.93, 147.31, 131.01, 121.62, 119.52, 119.17, 117.87, 117.61, 117.02, 116.98, 116.77, 111.77, 47.57, 25.42.

(*E*)-3-(5-fluoro-2-methoxyphenyl)-1-(4-(pyrrolidin-1-yl)phenyl)prop-2-en-1-one (**D1**). Orange crystals, Yield 55.9 %, m.p.: 137.5～138.2 ℃, HRMS (ESI) *m/z* calcd for C_20_H_20_FNO_2_^+^ (M + H^+^) 326.1557, found 326.1547. ^1^H NMR (400 MHz, CDCl_3_) *δ* 8.07～7.94 (m, 2H, Ar-H), 7.89～7.81 (m, 1H, Ar-H), 7.60 (d, *J* = 15.8 Hz, 1H, H*_β_*), 7.33 (dd, *J* = 15.8 Hz, 1H, H*_α_*), 7.07～6.97 (m, 1H, Ar-H), 6.85 (dd, *J* = 9.0, 4.4 Hz, 1H), 6.56 (d, *J* = 9.0 Hz, 1H, Ar-H), 6.50 (d, *J* = 8.9 Hz, 1H, Ar-H), 3.87 (s, 3H, -CH_3_), 3.47～3.25 (m, 4H, -CH_2_), 2.14～1.94 (m, 4H, -CH_2_). ^13^C NMR (101 MHz, CDCl_3_) *δ* 187.67, 150.99, 136.29, 131.01, 130.66, 125.57, 123.95, 117.05, 116.82, 114.51, 112.22, 110.62, 56.12, 47.53, 25.42.

(*E*)-3-(5-bromo-2-methoxyphenyl)-1-(4-(pyrrolidin-1-yl)phenyl)prop-2-en-1-one (**D2**). Orange crystals, Yield 72.1 %, m.p.: 130.5～130.9 ℃, HRMS (ESI) *m/z* calcd for C_20_H_20_BrNO_2_^+^ (M + H^+^) 386.0756, found 386.0745. ^1^H NMR (400 MHz, CDCl_3_) *δ* 8.16～7.86 (m, 3H, Ar-H), 7.65～7.55 (m, 2H, Ar-H), 7.27 (d, *J* = 15.8 Hz, 1H, H*_β_*), 6.85 (d, *J* = 15.8 Hz, 1H, H*_α_*), 6.65～6.50 (m, 2H, Ar-H), 3.88 (s, 3H, -CH_3_), 3.49～3.30 (m, 4H, -CH_2_), 2.16～1.97 (m, 4H, -CH_2_). ^13^C NMR (101 MHz, CDCl_3_) *δ* 187.60, 157.00, 101.00, 136.01, 131.04, 130.29, 127.92, 126.26, 125.67, 125.57, 123.98, 112.44, 110.90, 55.85, 47.58, 25.43.

(*E*)-3-(5-chloro-2-methoxyphenyl)-1-(4-(pyrrolidin-1-yl)phenyl)prop-2-en-1-one (**D3**). Yellow crystals, Yield 77.8 %, m.p.: 118.5～119.3 ℃, HRMS (ESI) *m/z* calcd for C_20_H_20_ClNO_2_^+^ (M + H^+^) 342.1262, found 342.1253. ^1^H NMR (400 MHz, CDCl_3_) *δ* 8.04～7.93 (m, 3H, Ar-H), 7.73 (d, *J* = 2.5 Hz, 1H, Ar-H), 7.61 (d, *J* = 15.7 Hz, 1H, H*_β_*), 7.41 (dd, *J* = 15.7 Hz, 1H, H*_α_*), 6.80 (d, *J* = 8.8 Hz, 1H, Ar-H), 6.61～6.53 (m, 2H, Ar-H), 3.88 (s, 3H, -CH_3_), 3.47～3.24 (m, 4H, -CH_2_), 2.19～1.85 (m, 4H, -CH_2_). ^13^C NMR (101 MHz, CDCl_3_) *δ* 187.58, 157.47, 101.01, 135.89, 133.22, 131.05, 130.80, 126.78, 125.56, 123.99, 112.95, 112.90, 110.90, 55.80, 47.58, 25.46, 25.44.

(*E*)-1-(4-(pyrrolidin-1-yl)phenyl)-3-(2-(trifluoromethoxy)phenyl)prop-2-en-1-one (**D4**). Orange crystals, Yield 41.6 %, m.p.: 121.9～122.4 ℃, HRMS (ESI) *m/z* calcd for C_20_H_18_F_3_NO_2_^+^ (M + H^+^) 362.1369, found 362.1359. ^1^H NMR (400 MHz, CDCl_3_) *δ* 8.03～7.90 (m, 3H, Ar-H), 7.76 (dd, *J* = 15.8 Hz, 1H, H*_β_*), 7.61 (d, *J* = 15.8 Hz, 1H, H*_α_*), 7.40 (d, *J* = 7.7Hz, 1H, Ar-H), 7.36～7.26 (m, 2H, Ar-H), 6.61～6.53 (m, 2H, Ar-H), 3.43～3.35 (m, 4H, -CH_2_), 2.10～1.99 (m, 4H, -CH_2_). ^13^C NMR (101 MHz, CDCl_3_) *δ* 191.11, 149.80, 147.43, 131.55, 130.79, 130.21, 126.90, 121.62, 121.26, 121.24, 120.27, 111.80, 47.60, 25.42.

(*E*)-1-(4-(pyrrolidin-1-yl)phenyl)-3-(3-(trifluoromethoxy)phenyl)prop-2-en-1-one (**D5**). Orange crystals, Yield 67.0 %, m.p.: 127.1～128.1 ℃, HRMS (ESI) *m/z* calcd for C_20_H_18_F_3_NO_2_^+^ (M + H^+^) 362.1369, found 362.1359. ^1^H NMR (400 MHz, CDCl_3_) *δ* 8.03～7.95 (m, 2H, Ar-H), 7.72 (d, *J* = 15.6 Hz, 1H, H*_β_*), 7.58 (d, *J* = 15.6 Hz, 1H, H*_α_*), 7.53 (d, *J* = 7.7Hz, 1H, Ar-H), 7.47 (s, 1H, Ar-H), 7.42 (t, *J* = 8.0 Hz, 1H, Ar-H), 7.22 (dd, *J* = 8.2, 2.4Hz, 1H, Ar-H), 6.61～6.48 (m, 2H, Ar-H), 3.43～3.32 (m, 4H, -CH_2_), 2.10～1.98 (m, 4H, -CH_2_). ^13^C NMR (101 MHz, CDCl_3_) *δ* 187.31, 101.12, 134.99, 131.11, 130.61, 129.08, 128.48, 126.99, 125.51, 125.25, 121.35, 121.34, 110.95, 47.58, 25.43.

(*E*)-1-(4-(pyrrolidin-1-yl)phenyl)-3-(4-(trifluoromethoxy)phenyl)prop-2-en-1-one (**D6**). Orange crystals, Yield 63.9 %, m.p.: 131.8～132.4 ℃, HRMS (ESI) *m/z* calcd for C_20_H_18_F_3_NO_2_^+^ (M + H^+^) 362.1369, found 362.1360. ^1^H NMR (400 MHz, CDCl_3_) *δ* 8.03～7.95 (m, 2H, Ar-H), 7.73 (d, *J* = 15.6 Hz, 1H, H*_β_*), 7.69～7.61 (m, 2H, Ar-H), 7.55 (d, *J* = 15.6 Hz, 1H, H*_α_*), 7.23 (d, *J* = 7.5 Hz, 2H, Ar-H), 6.60～6.52 (m, 2H, Ar-H), 3.42～3.34 (m, 4H, -CH_2_), 2.10～1.99 (m, 4H, -CH_2_). ^13^C NMR (101 MHz, CDCl_3_) *δ* 187.00, 101.16, 140.32, 137.77, 131.08, 130.18, 126.68, 125.20, 123.86, 121.86, 120.00, 110.98, 47.60, 25.42.

**Intermediate 2**

**
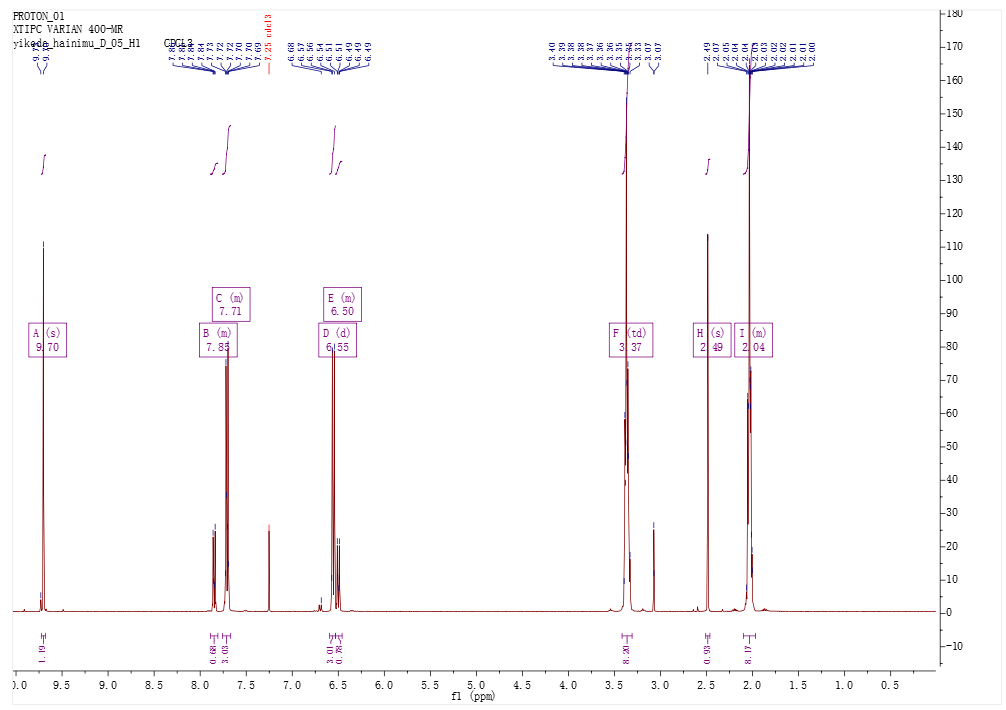
**

**Intermediate 5**

**
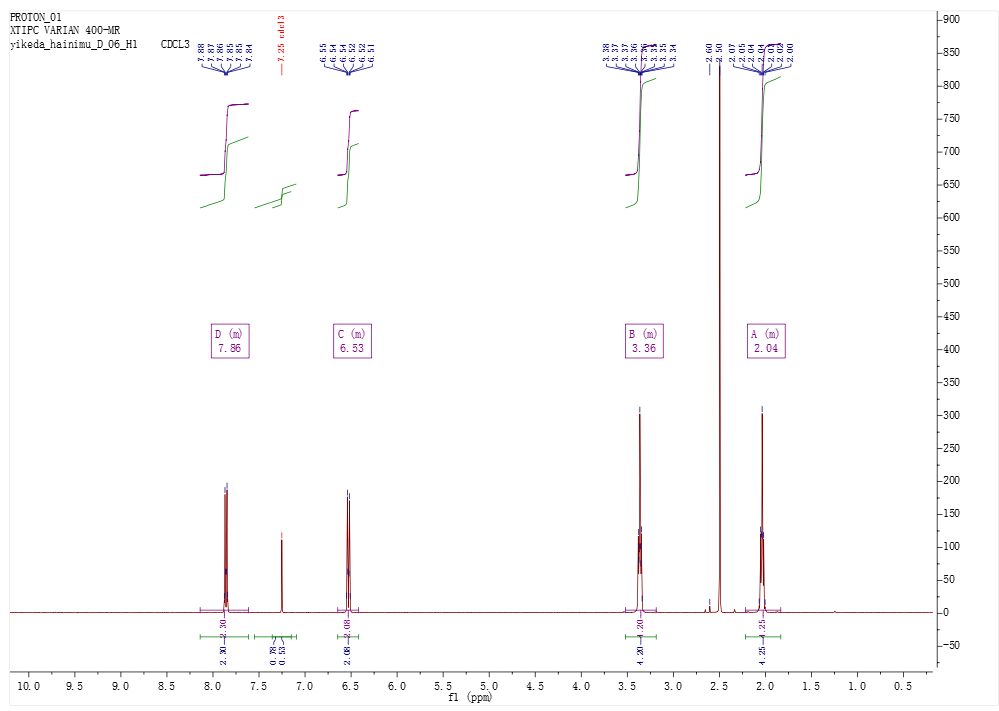
**

**Compound A1**


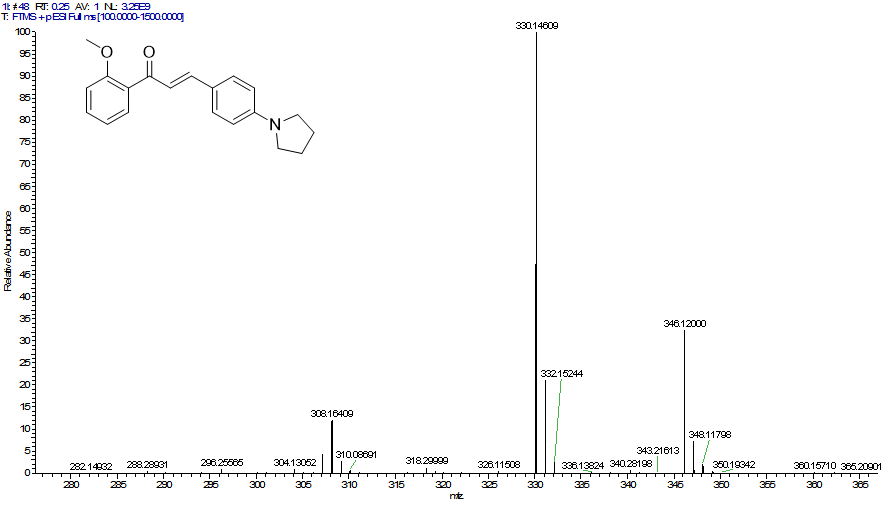


**
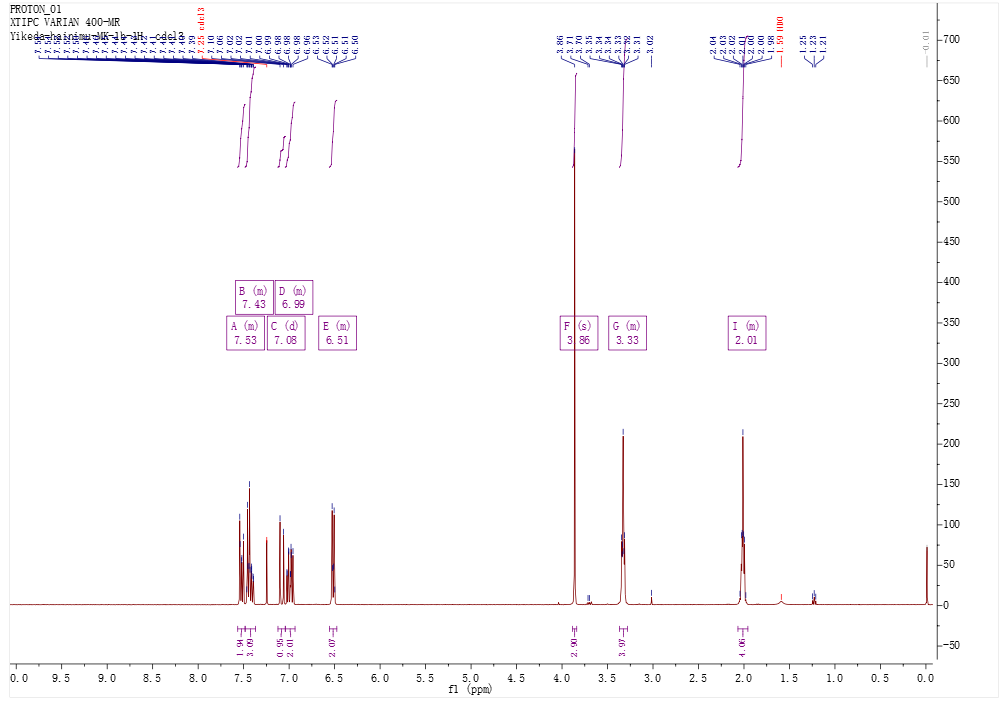
**

**
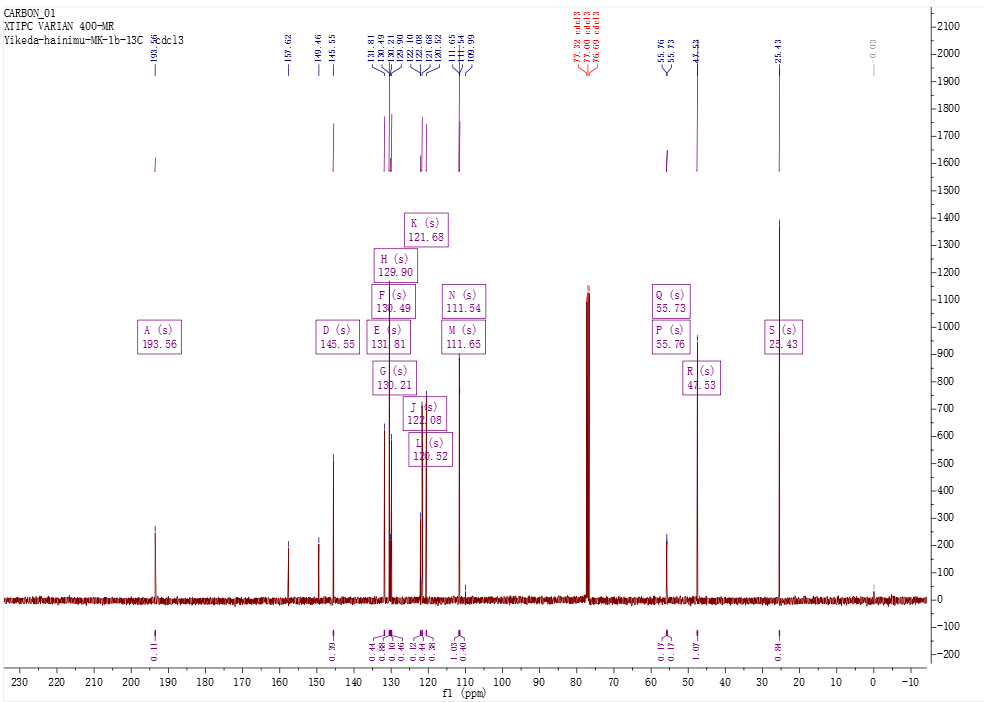
**

**Compound A2**


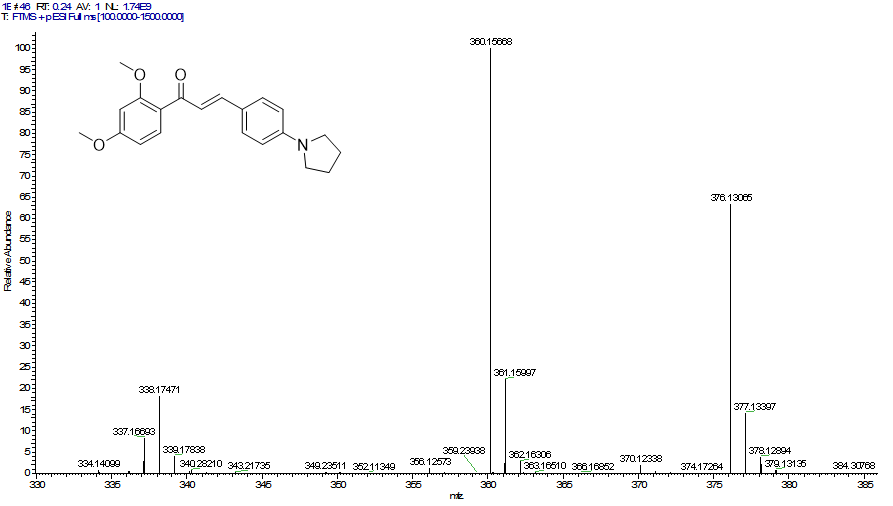


**
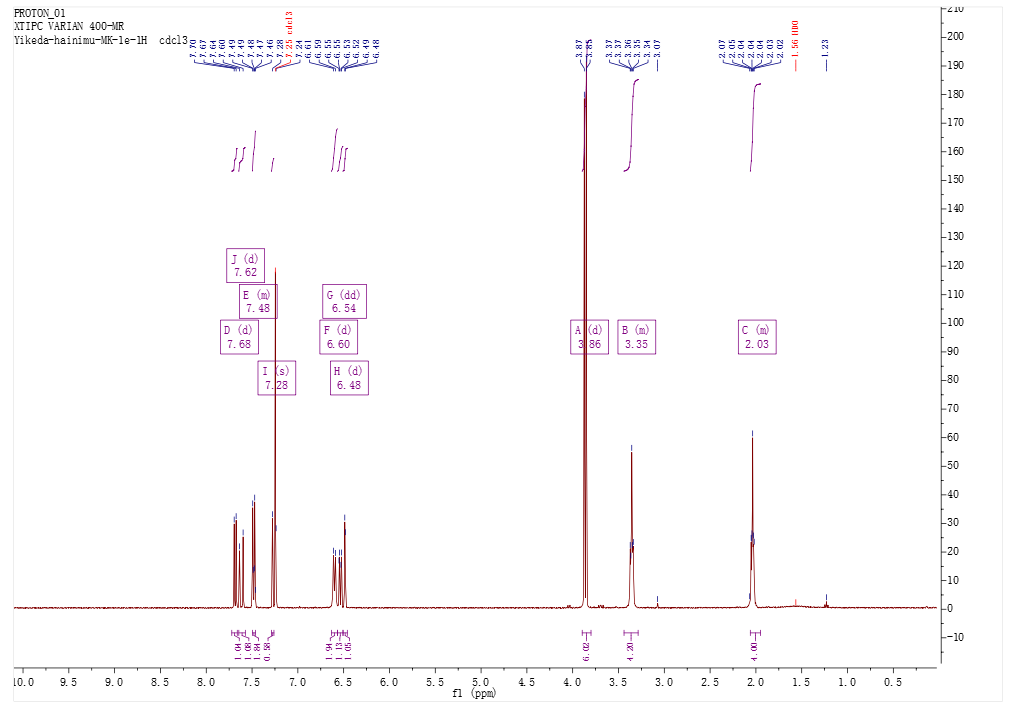
**

**
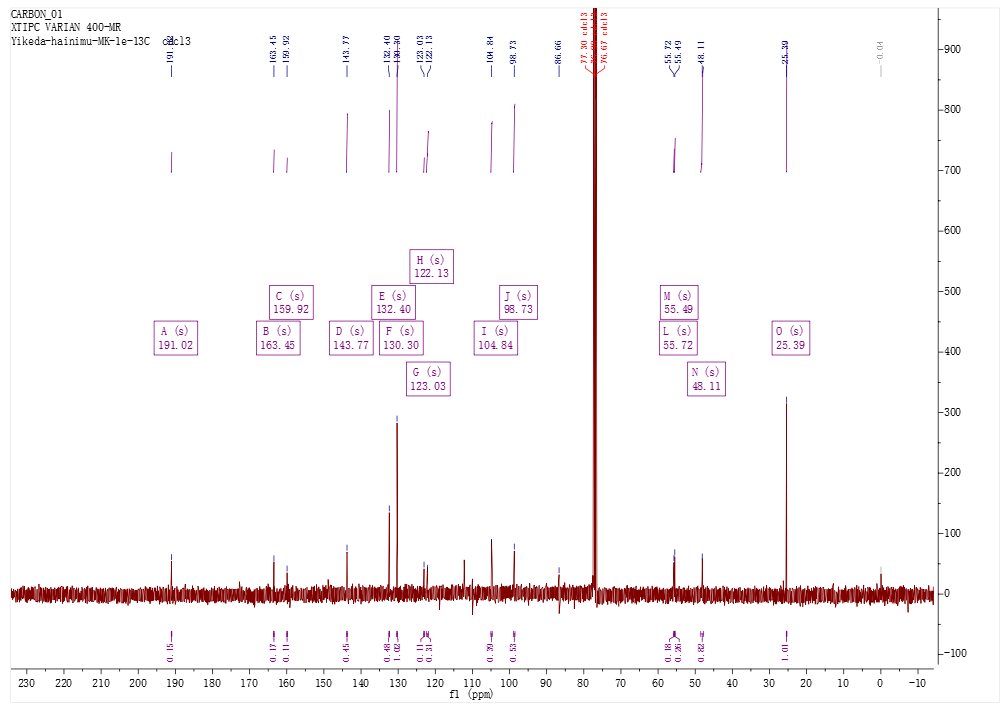
**

**Compound A3**


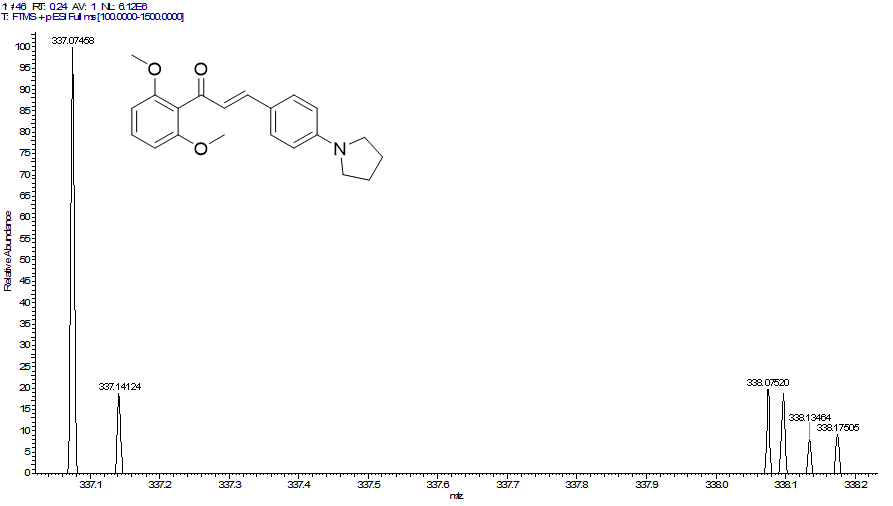


**
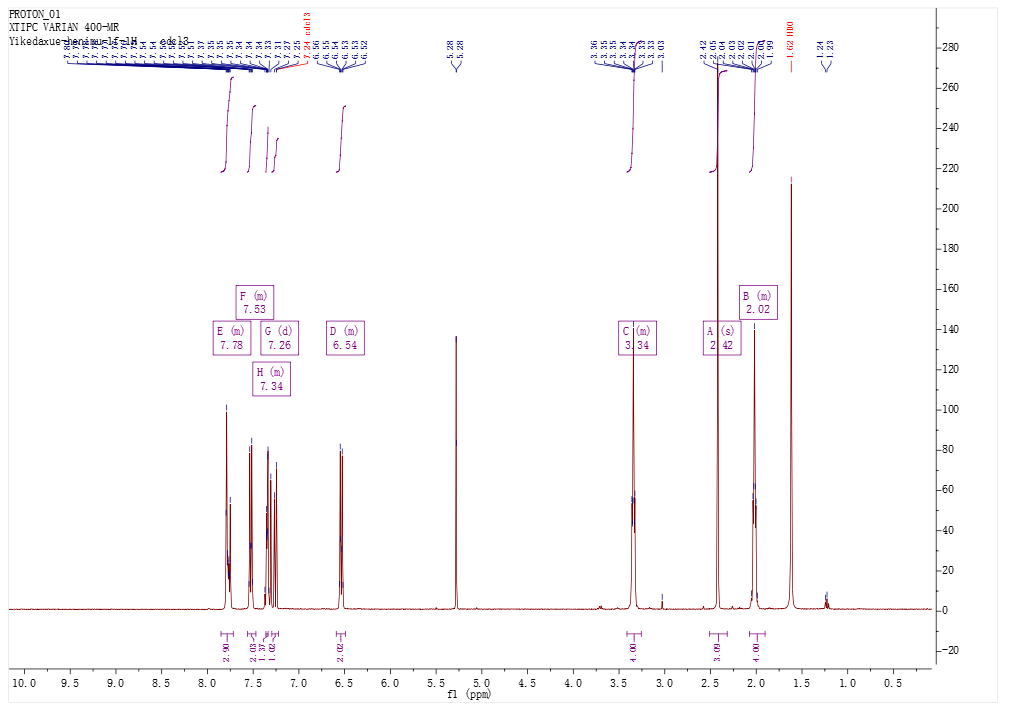

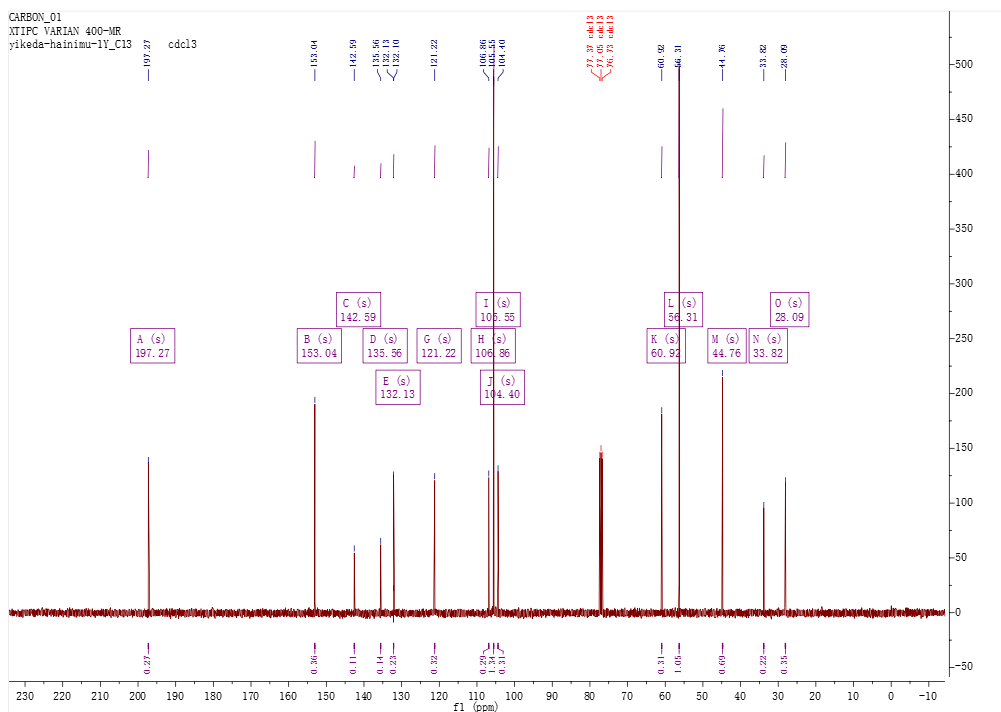
**

**Compound A4**


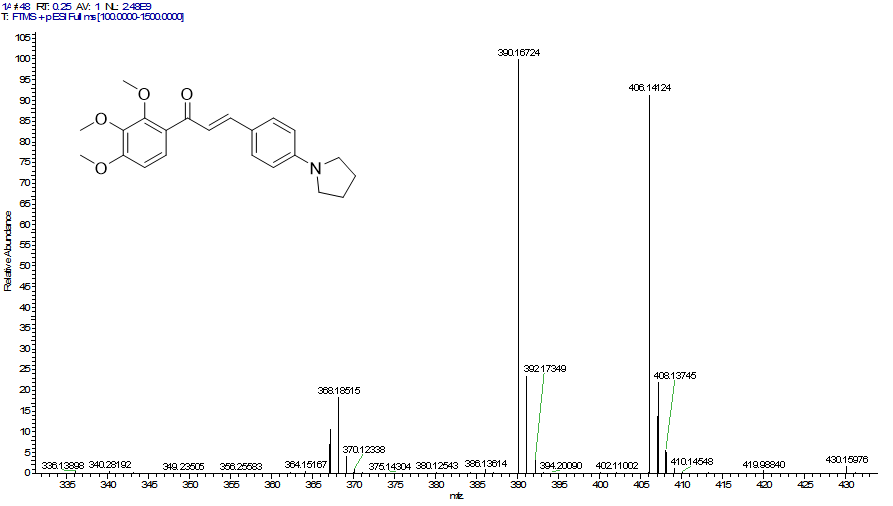


**
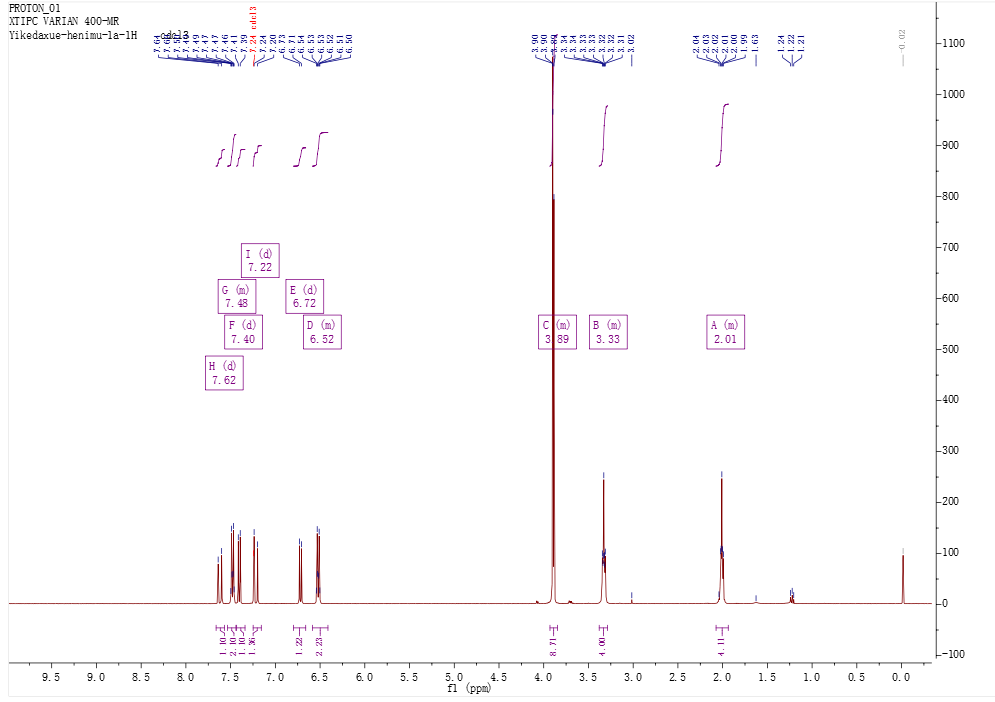
**

**
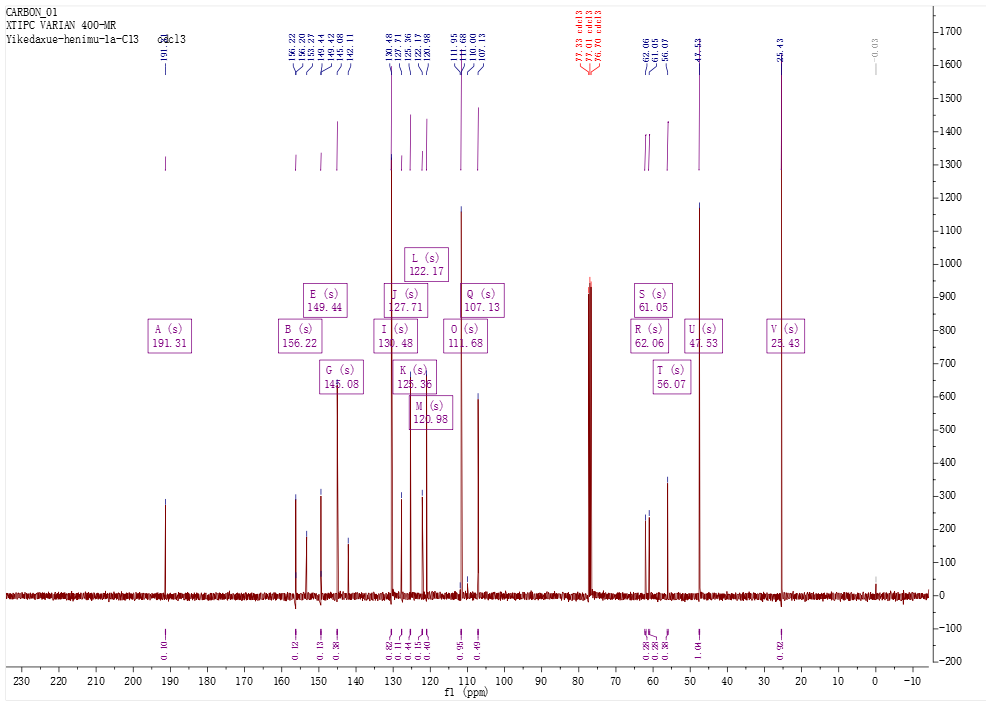
**

**Compound A5**


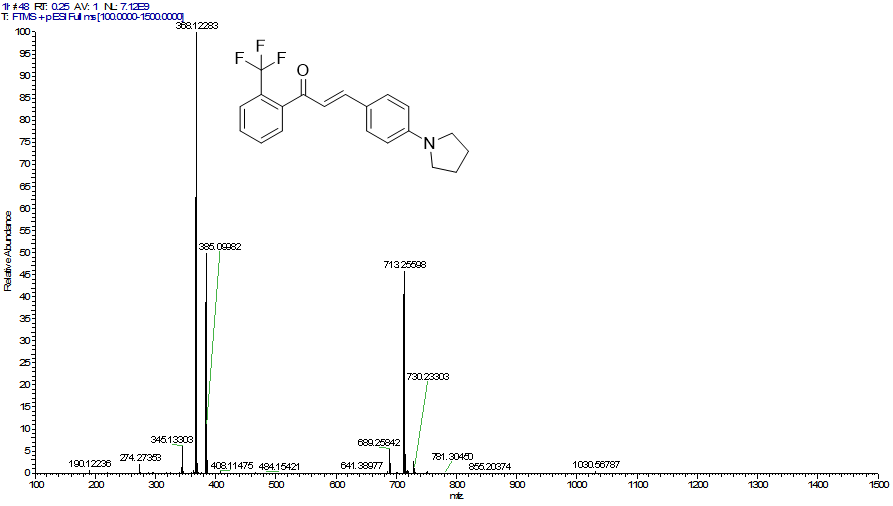


**
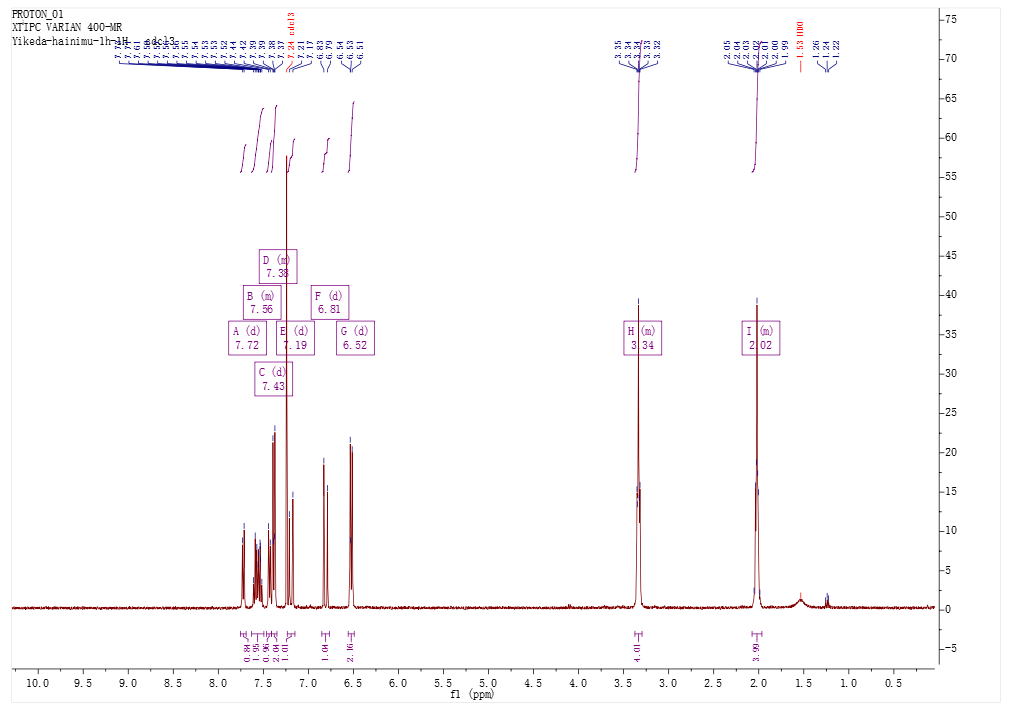
**

**
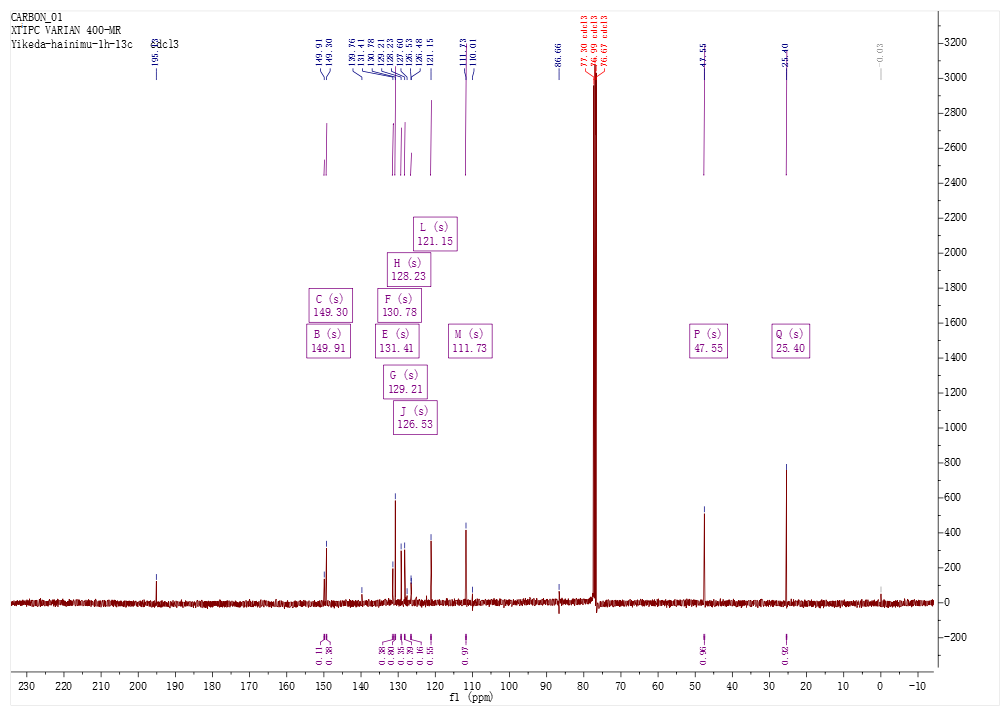
**

**Compound A6**


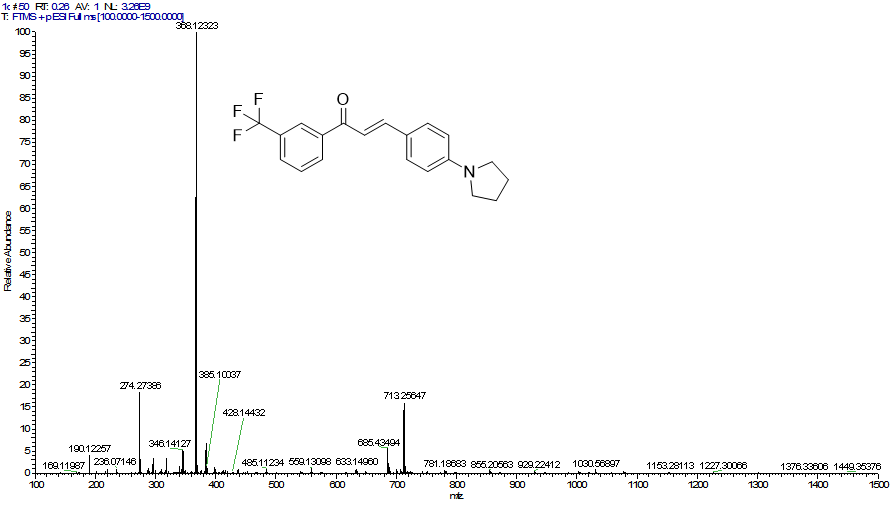


**
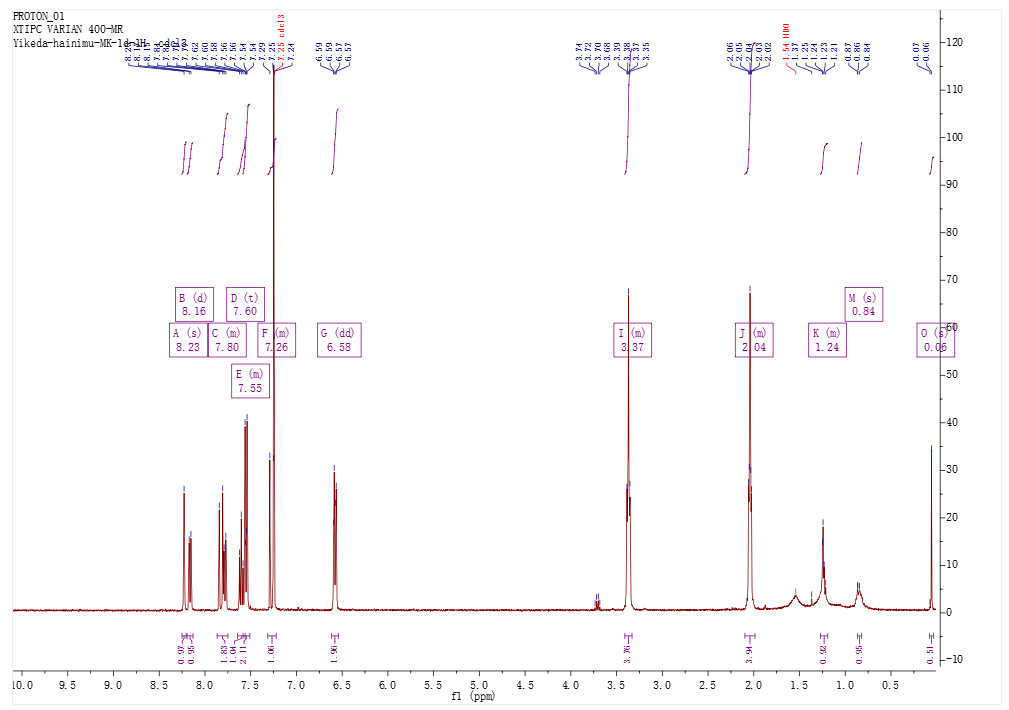
**

**
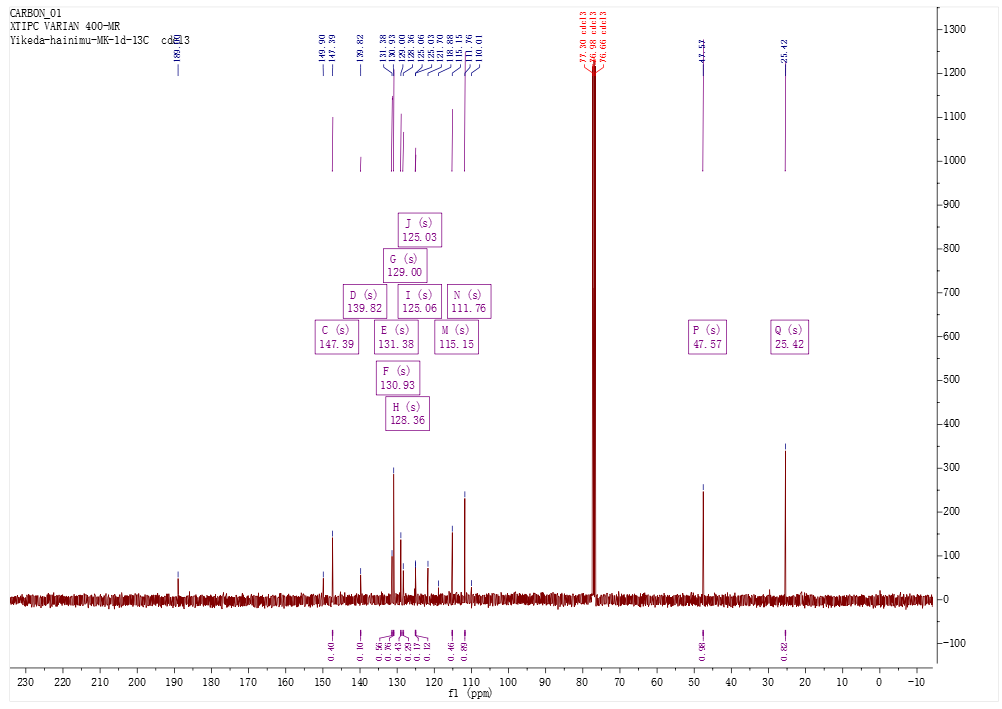
**

**Compound A7**


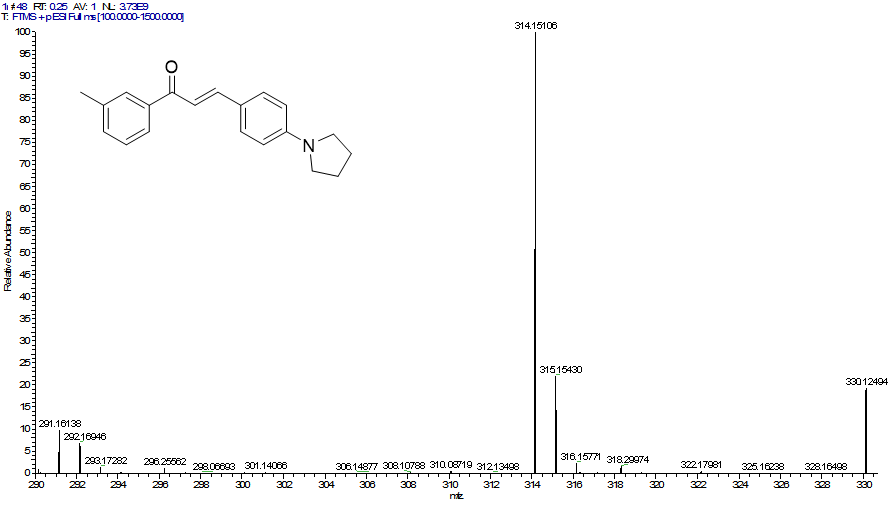


**
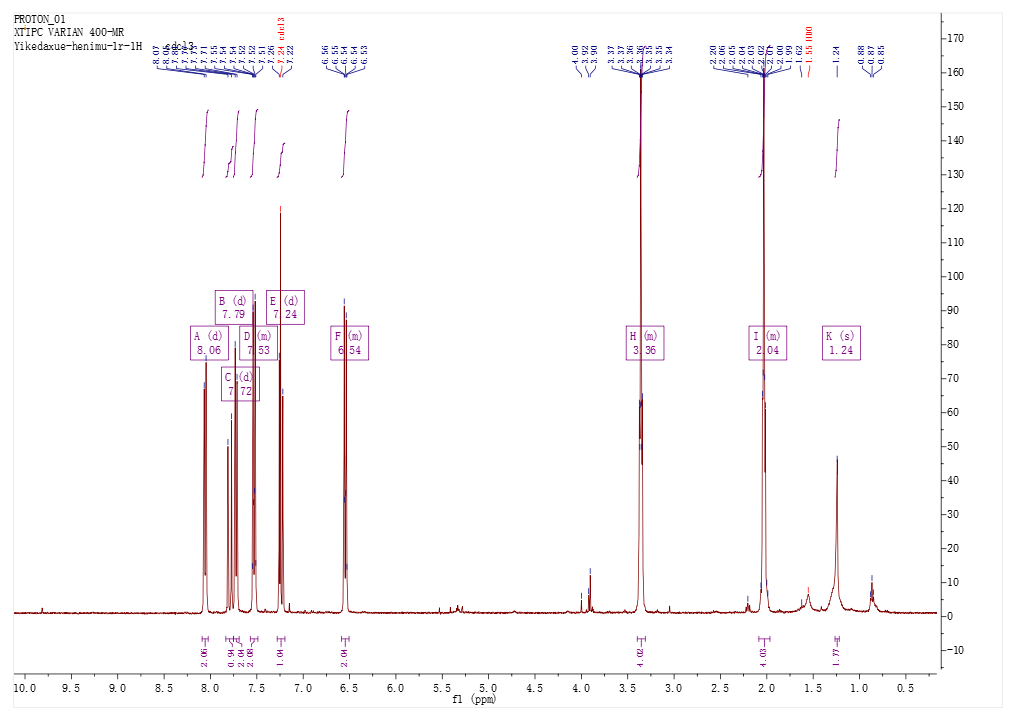
**

**
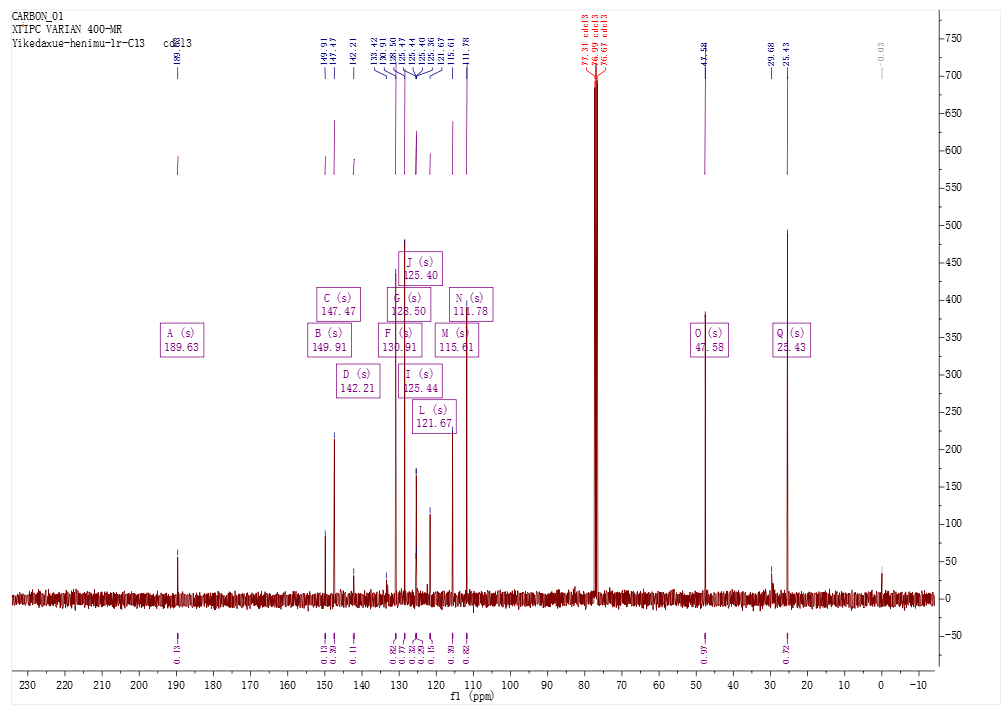
**

**Compound A8**


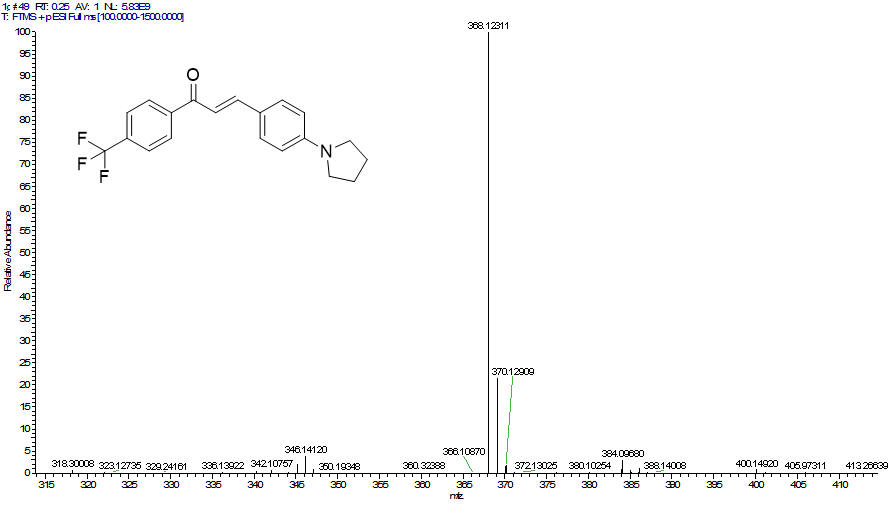


**
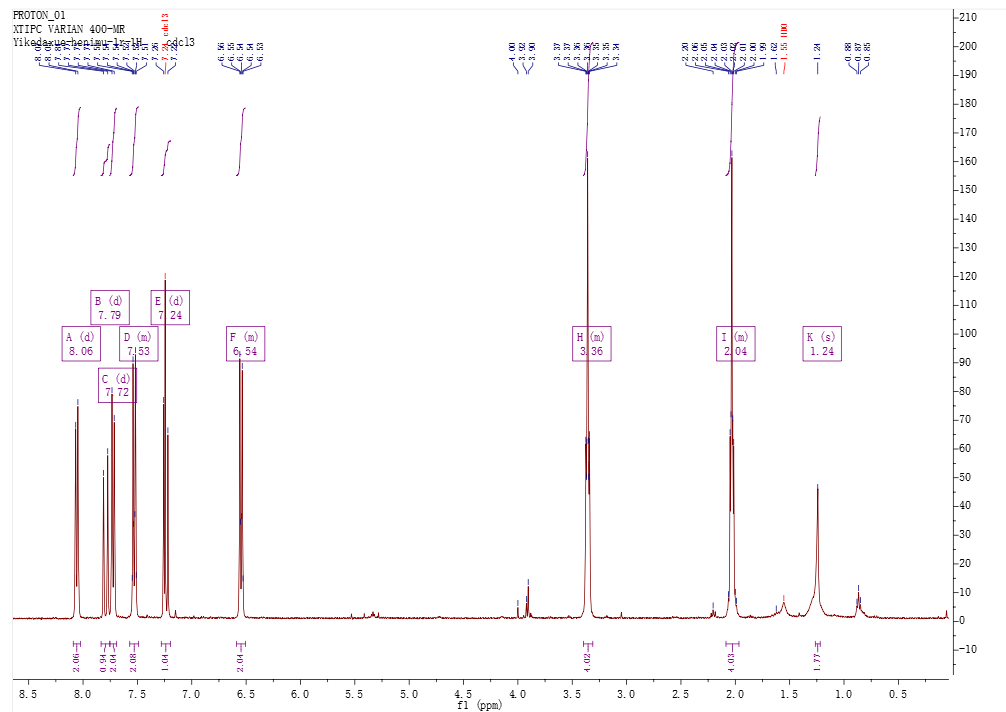
**

**
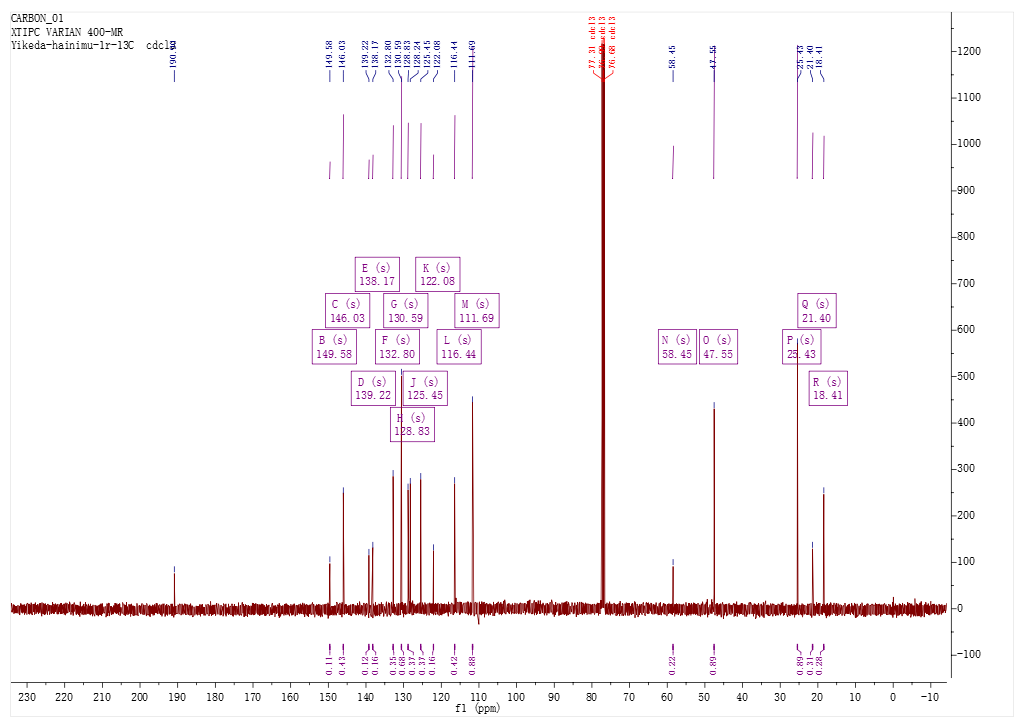
**

**Compound A9**


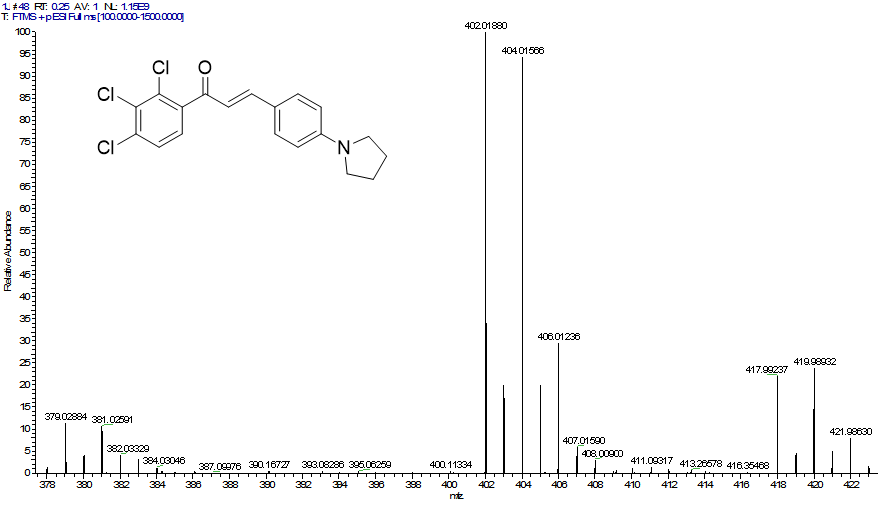


**
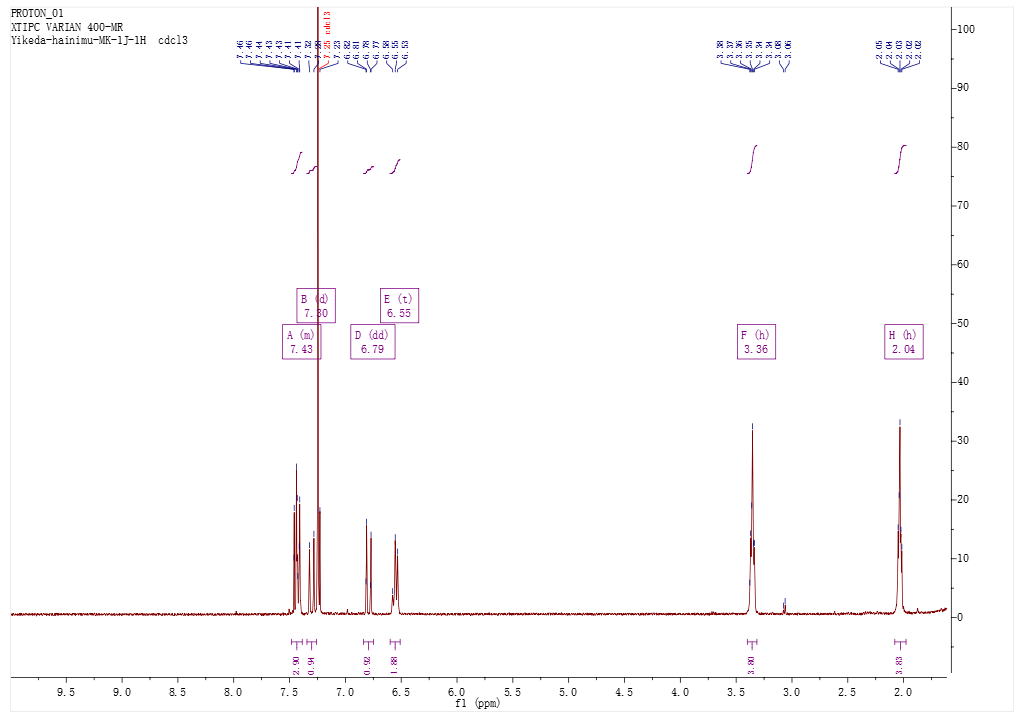
**

**
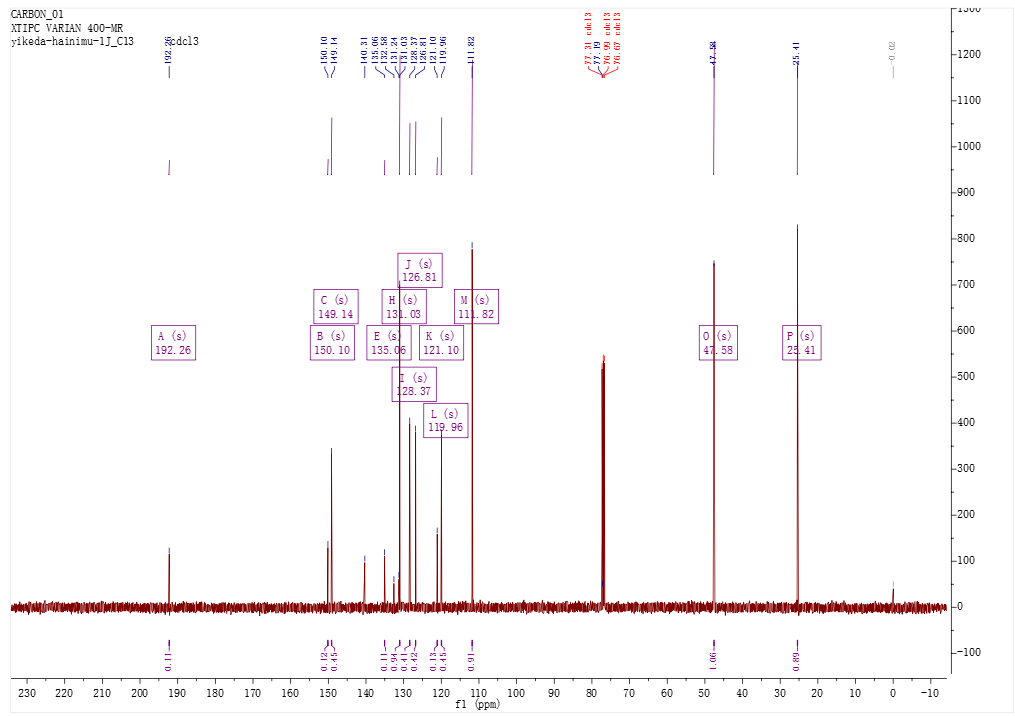
**

**Compound B1**


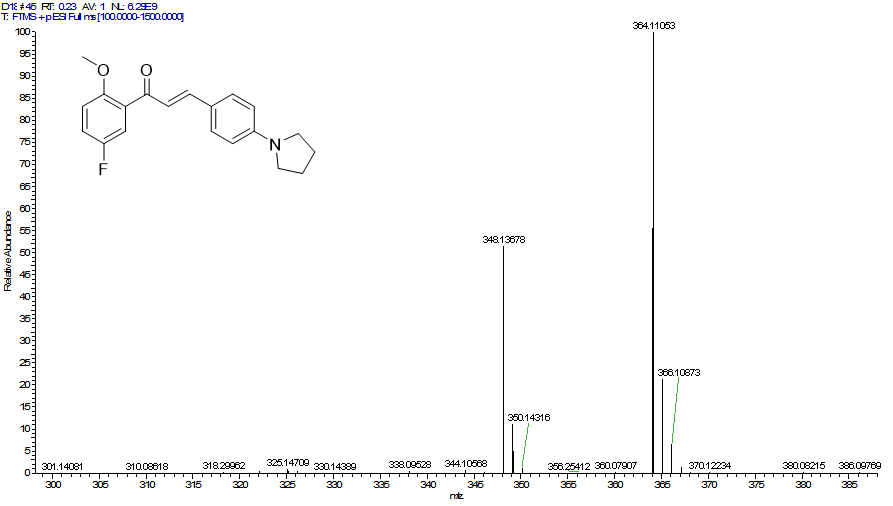


**
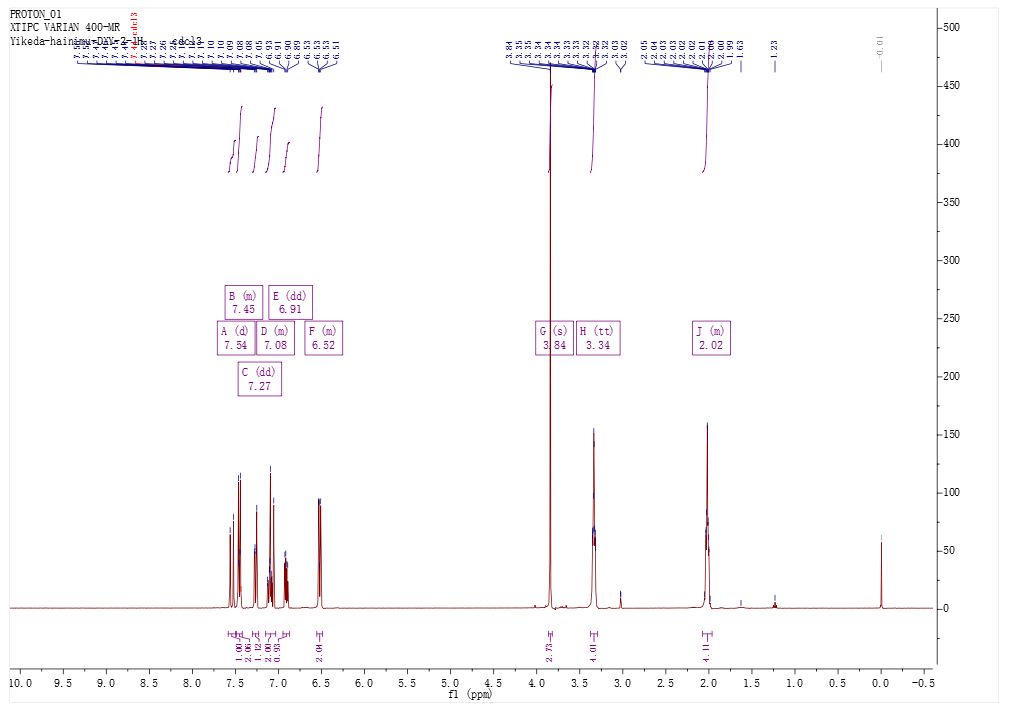
**

**
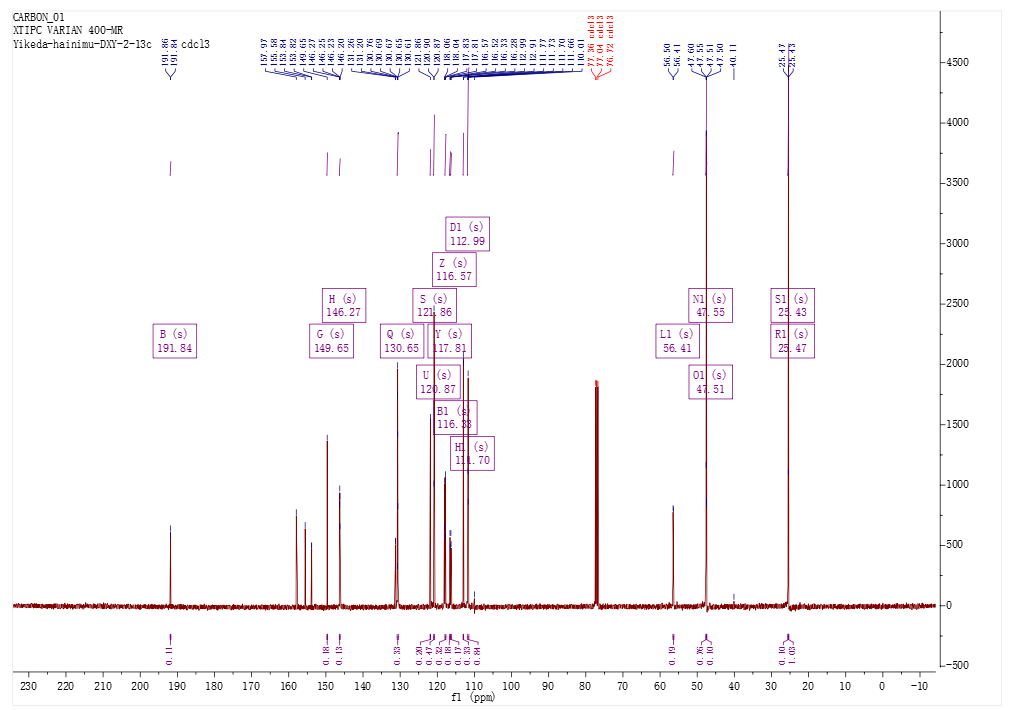
**

**Compound B2**


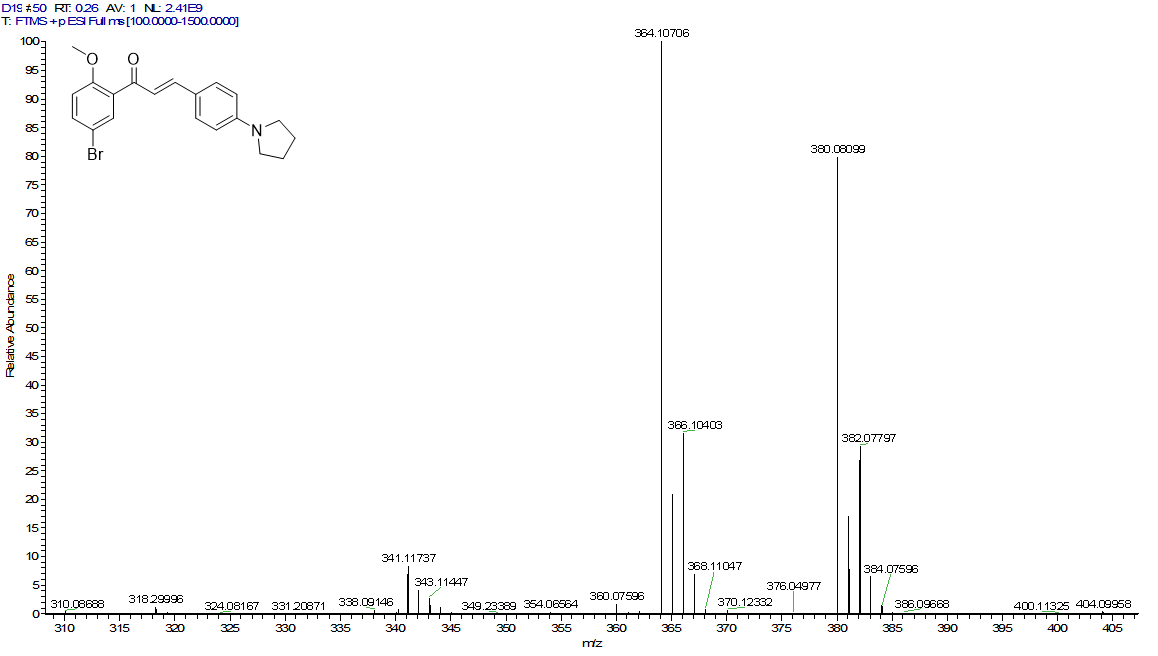


**
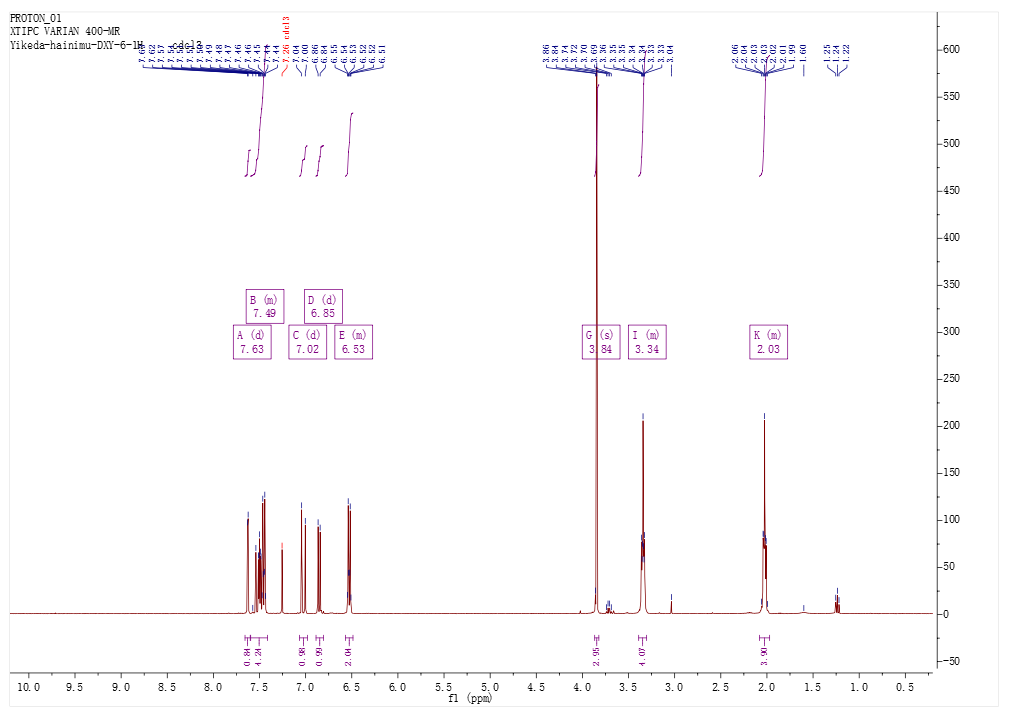
**

**
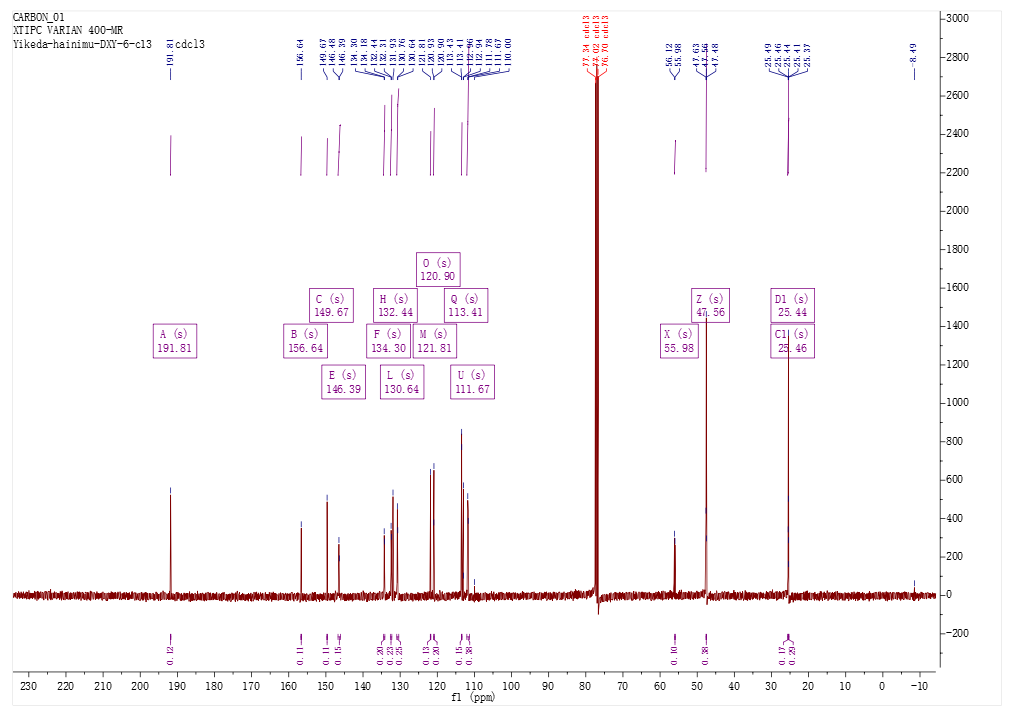
**

**Compound B3**


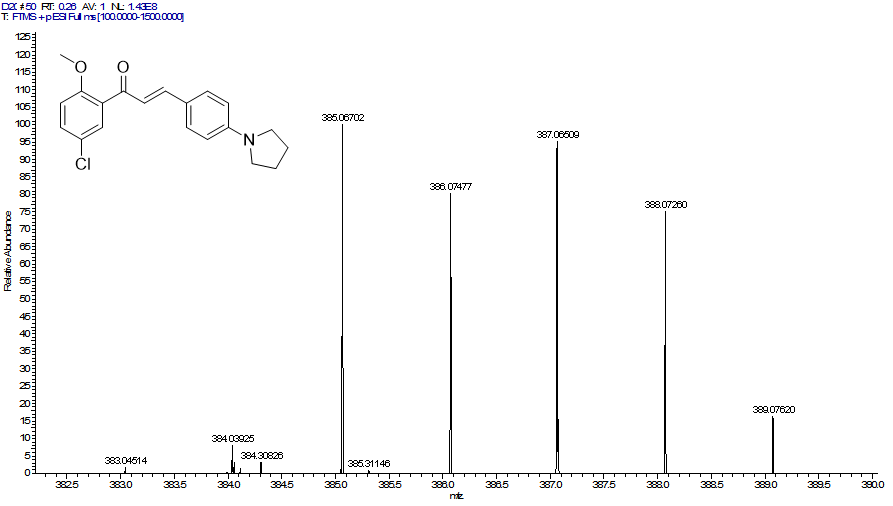


**
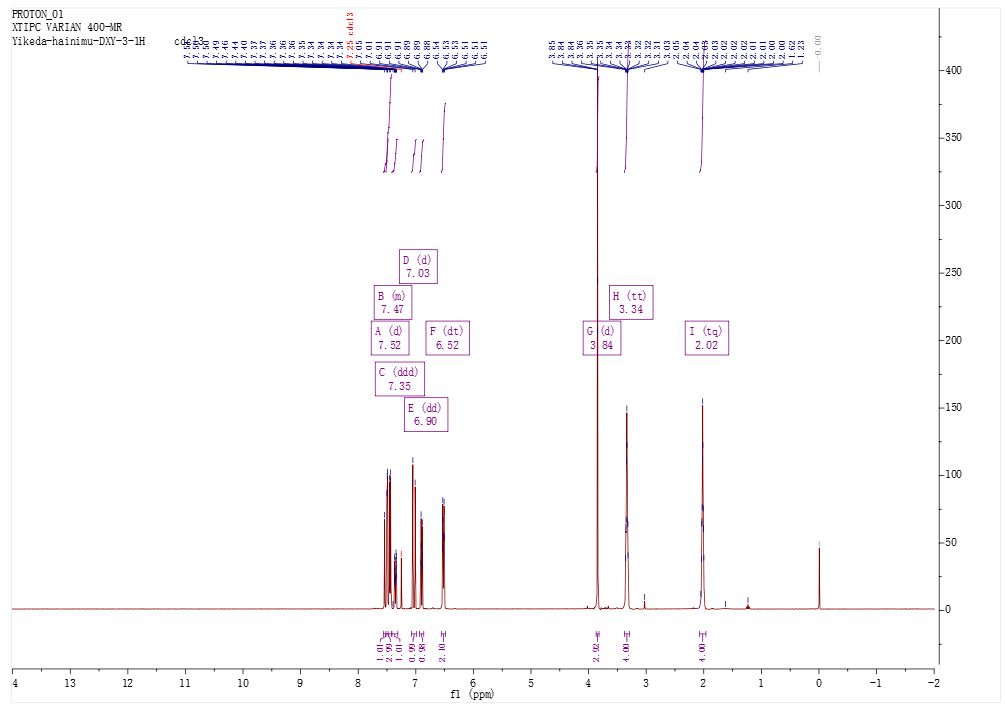
**

**
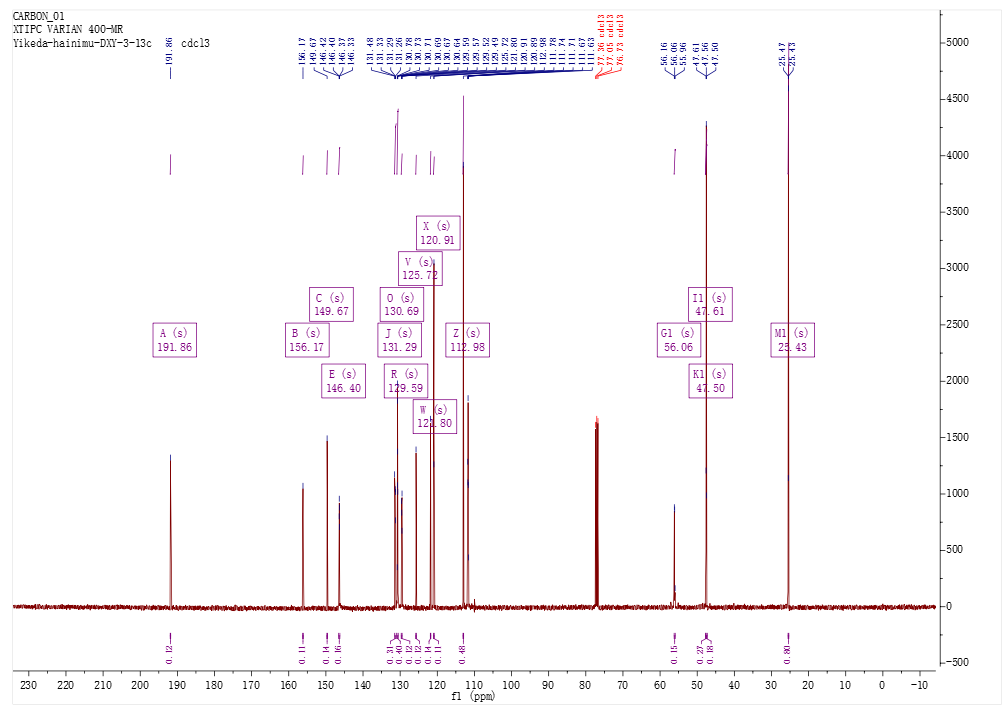
**

**Compound B4**


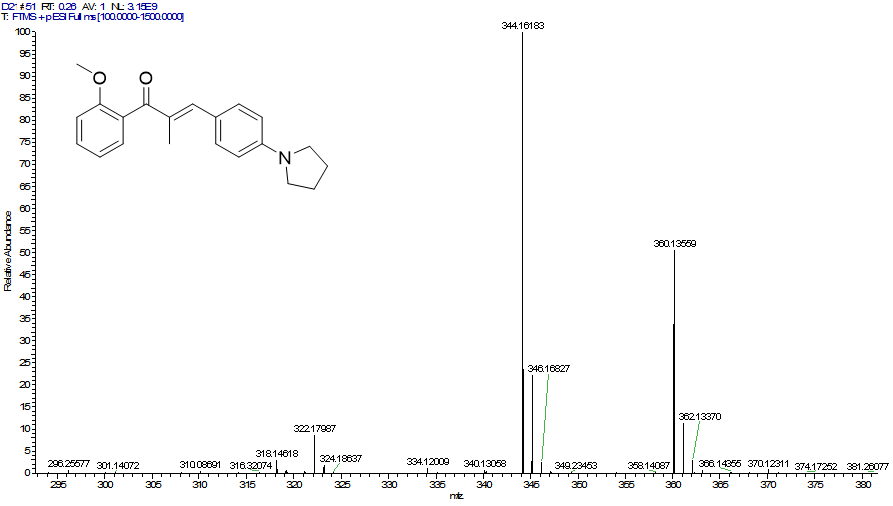


**
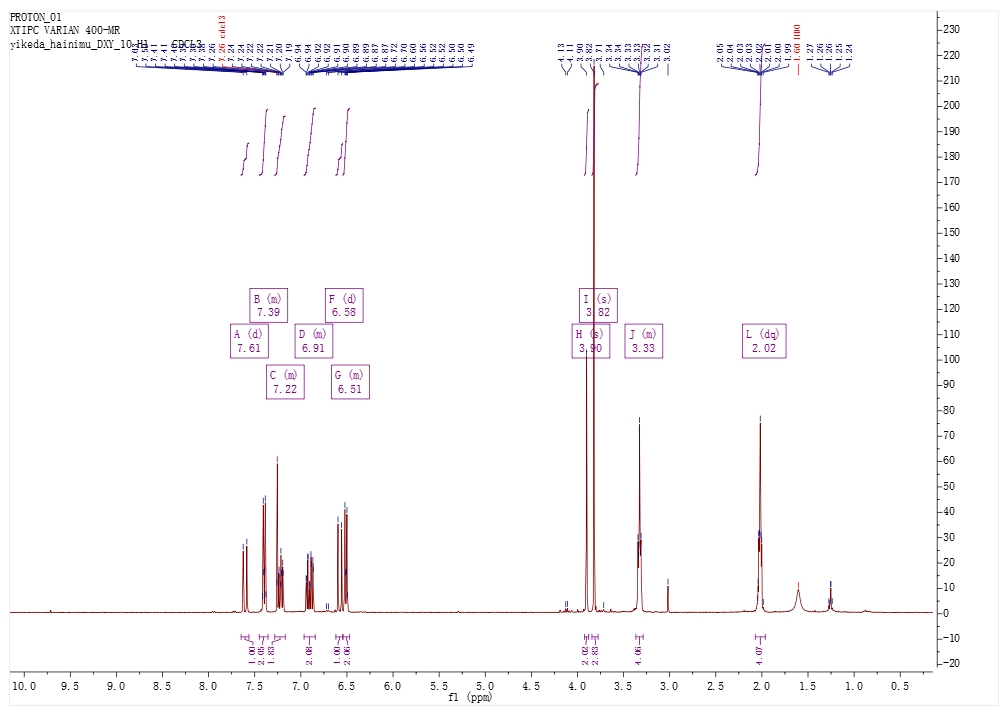
**

**
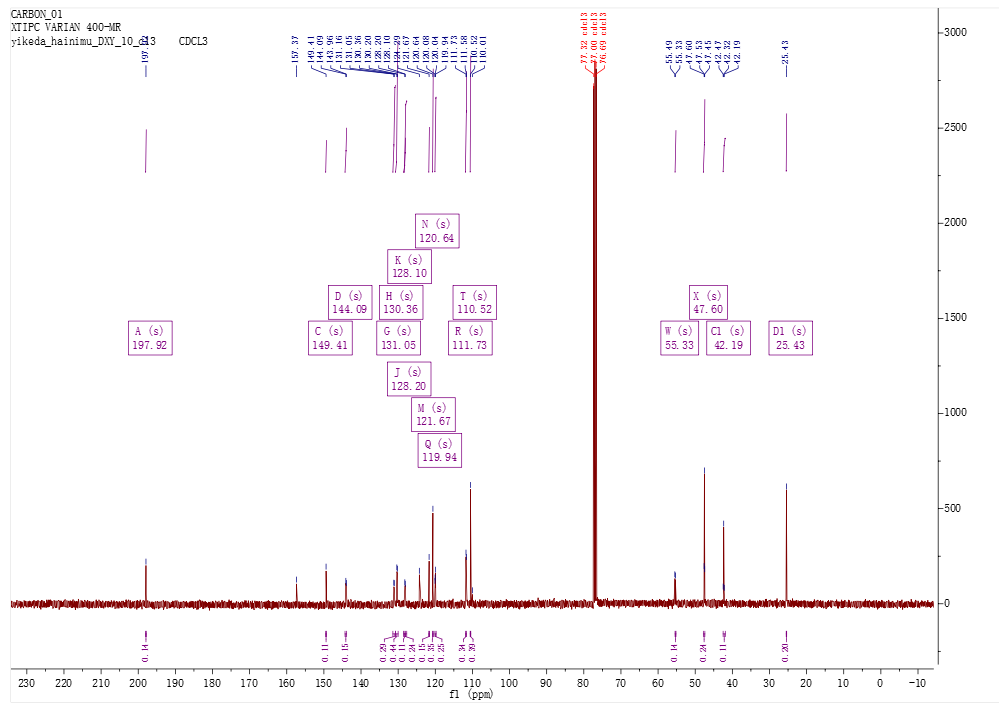
**

**Compound B5**


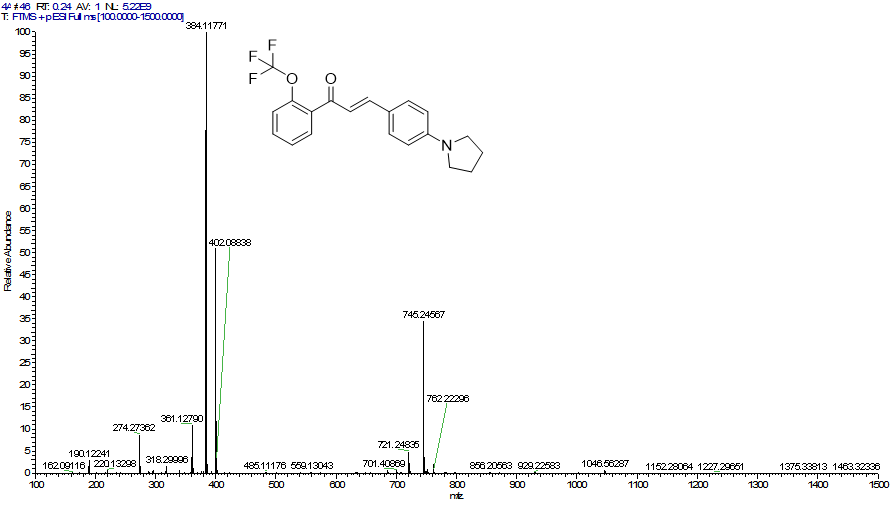


**
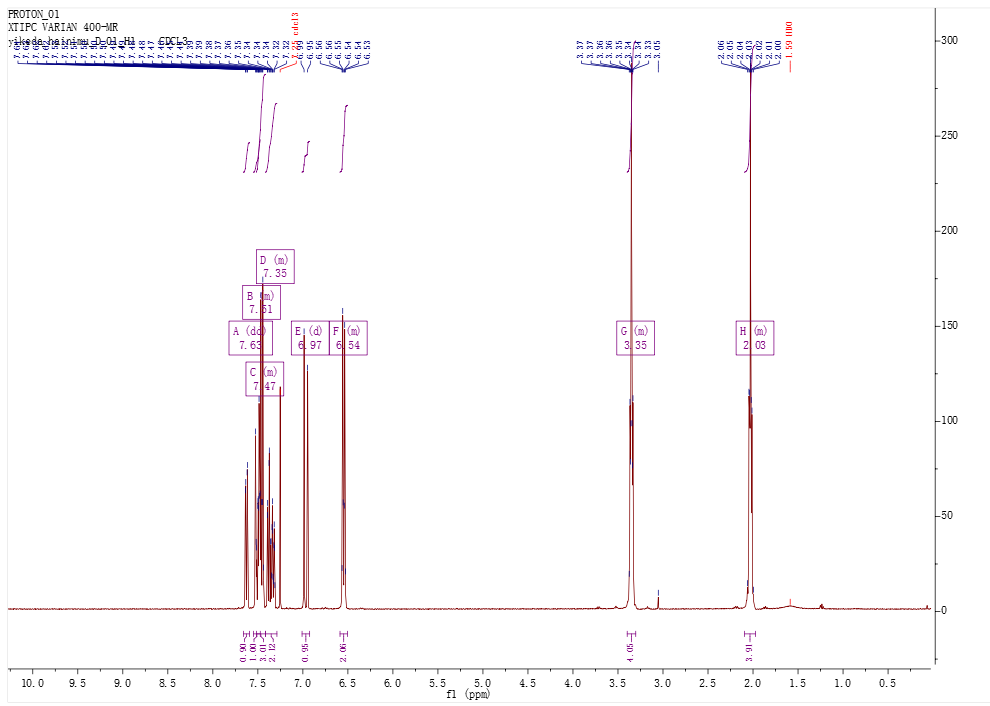
**

**
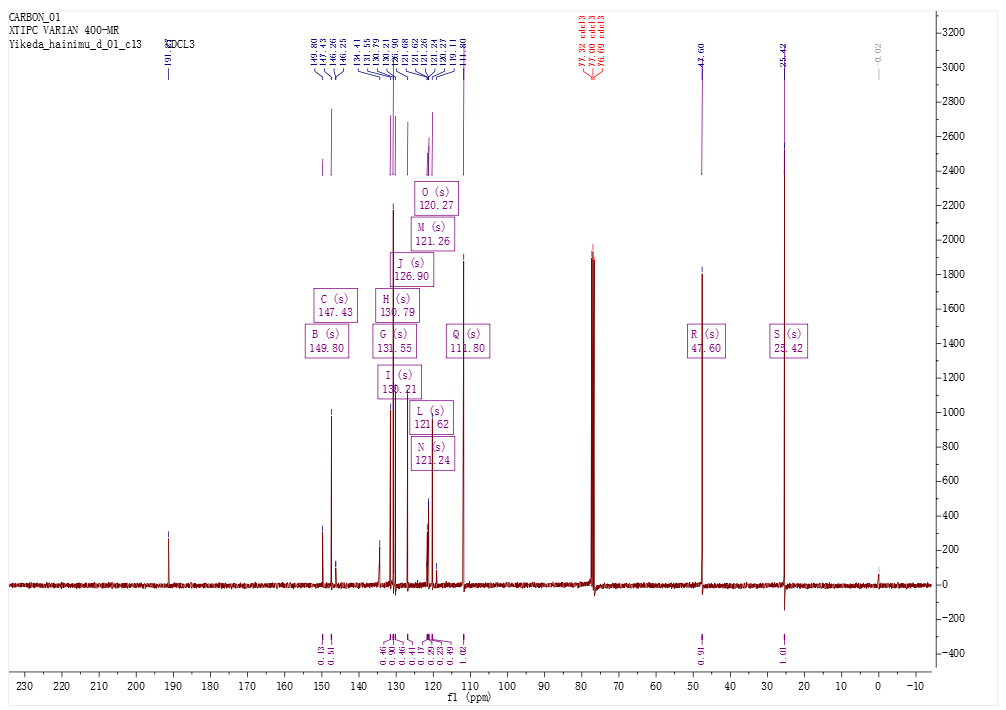
**

**Compound B6**


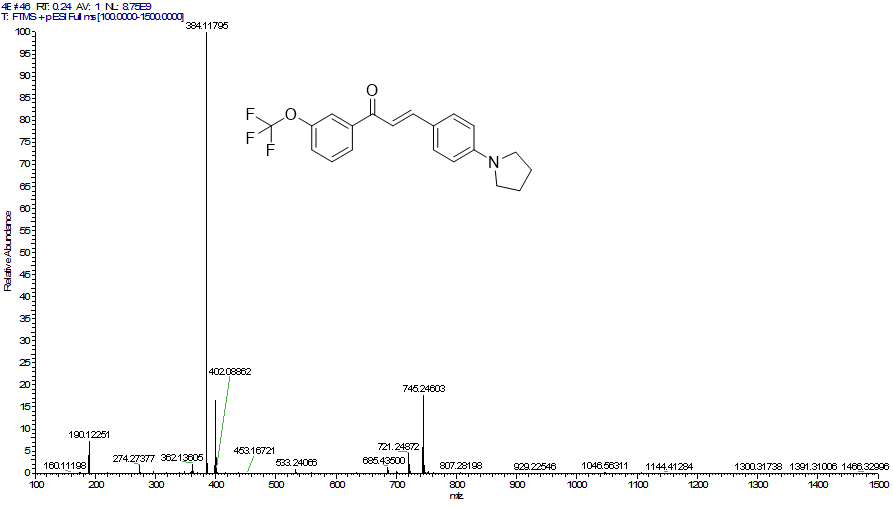


**
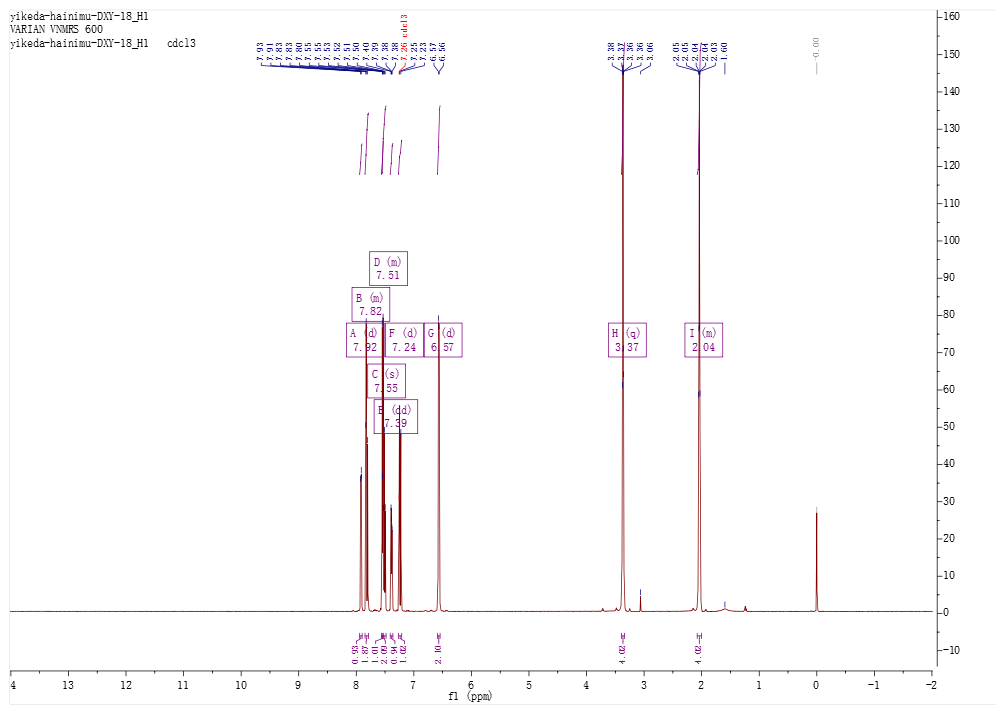
**

**
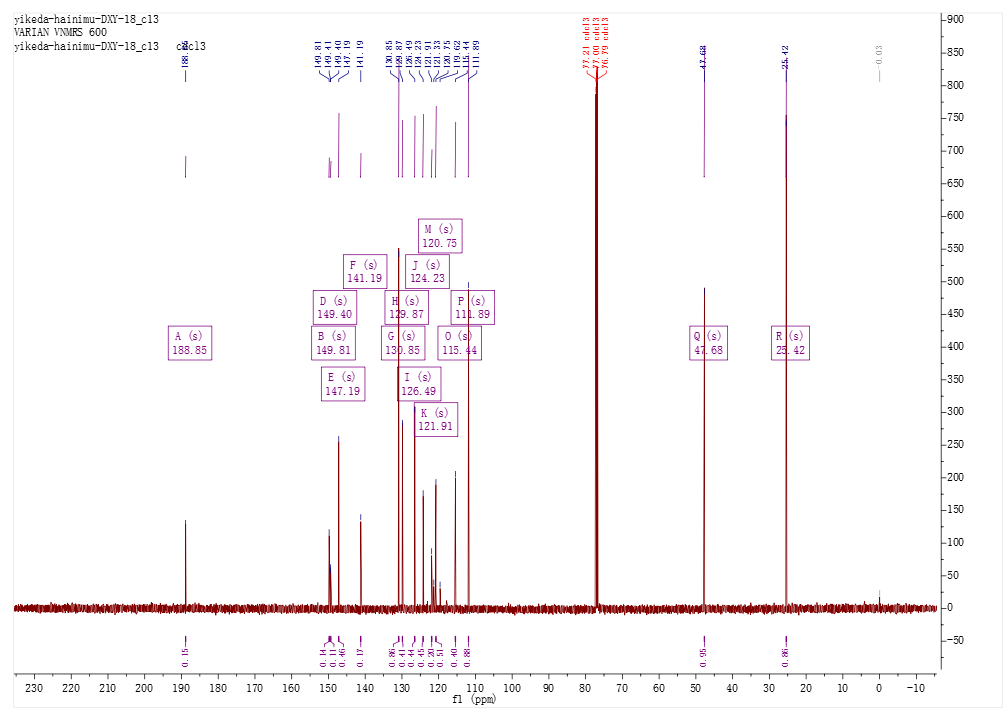
**

**Compound B7**


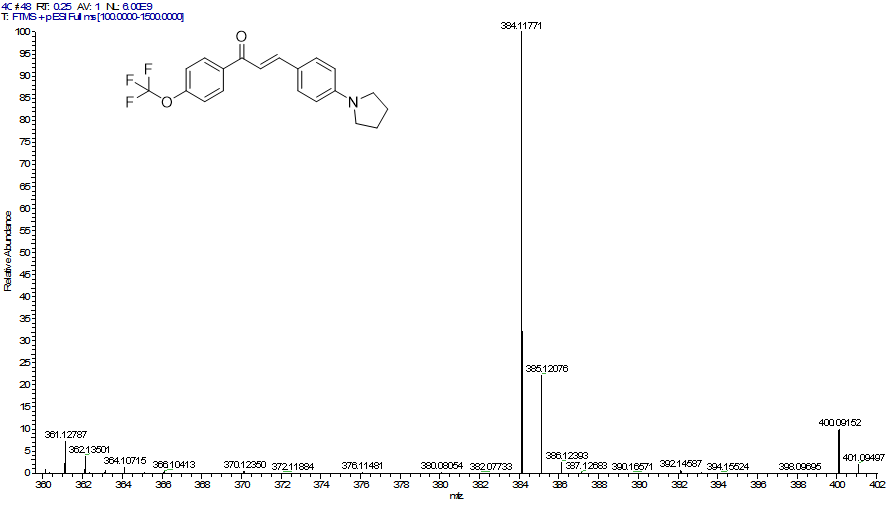


**
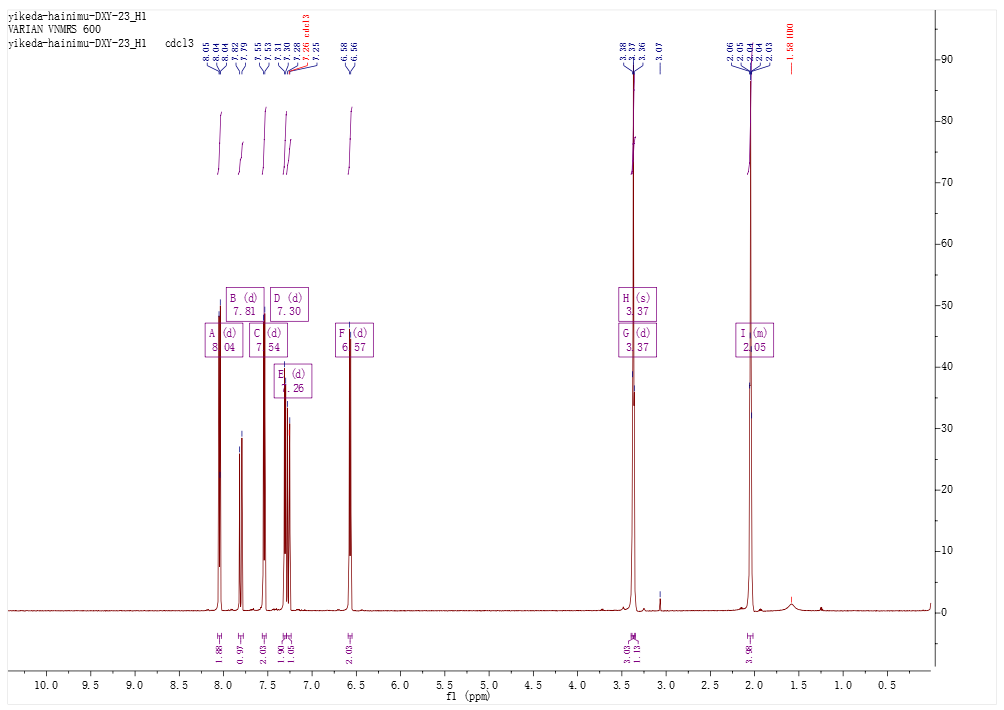
**

**
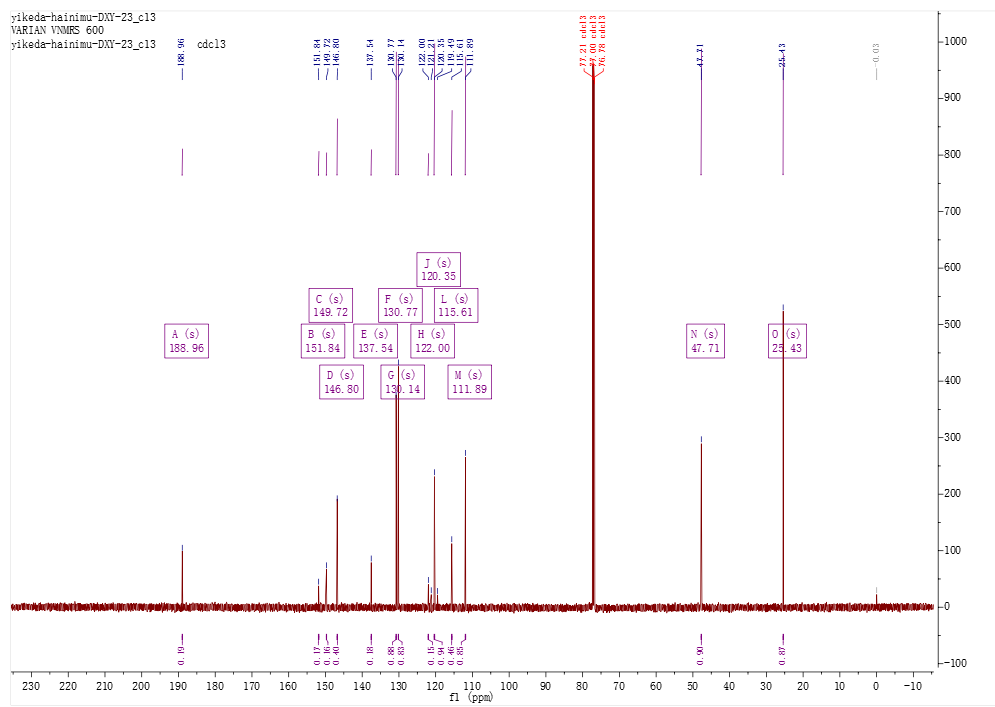
**

**Compound C1**


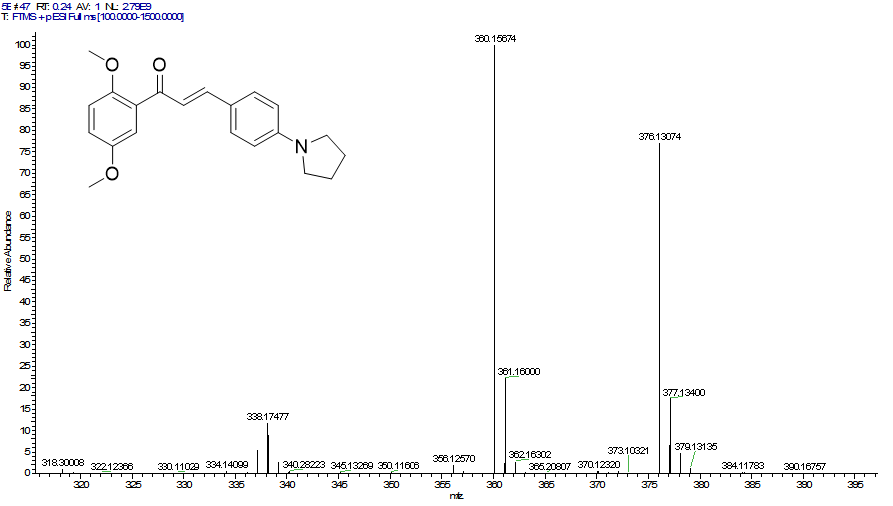


**
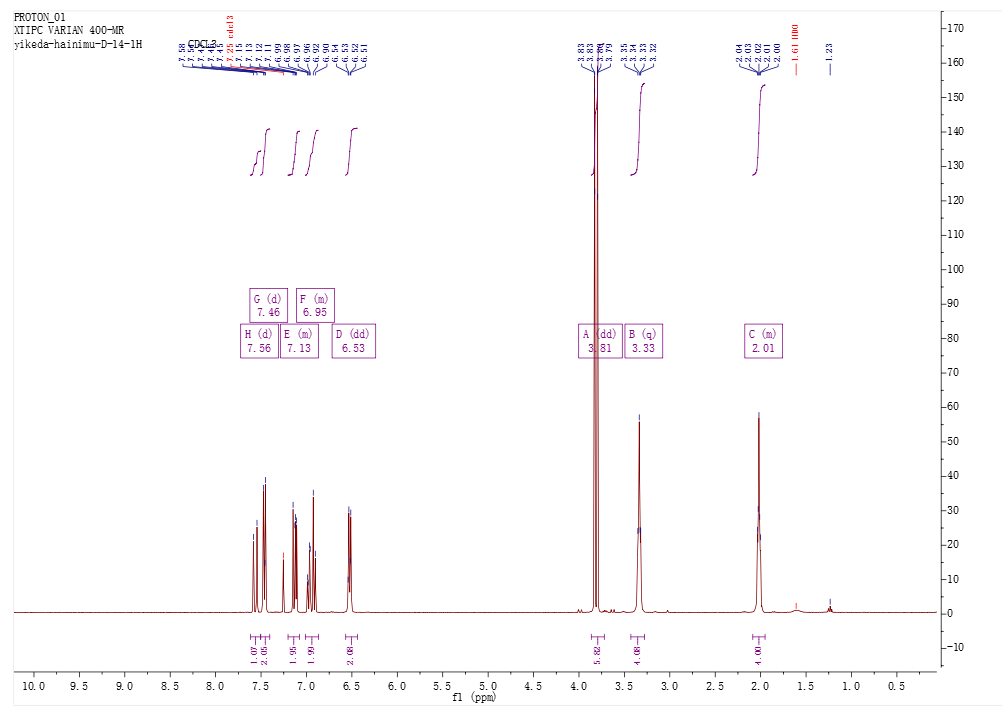
**

**
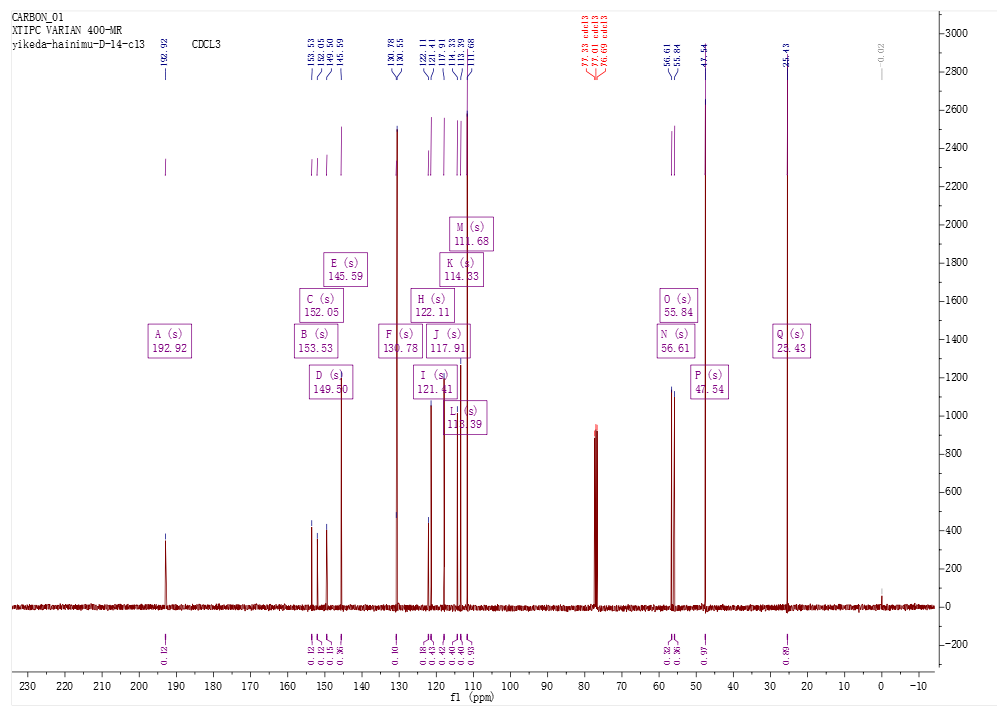
**

**Compound C2**


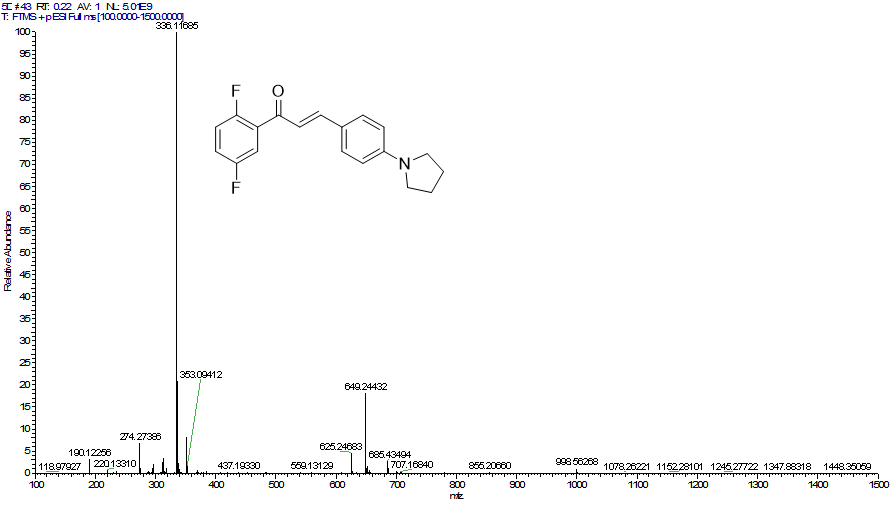


**
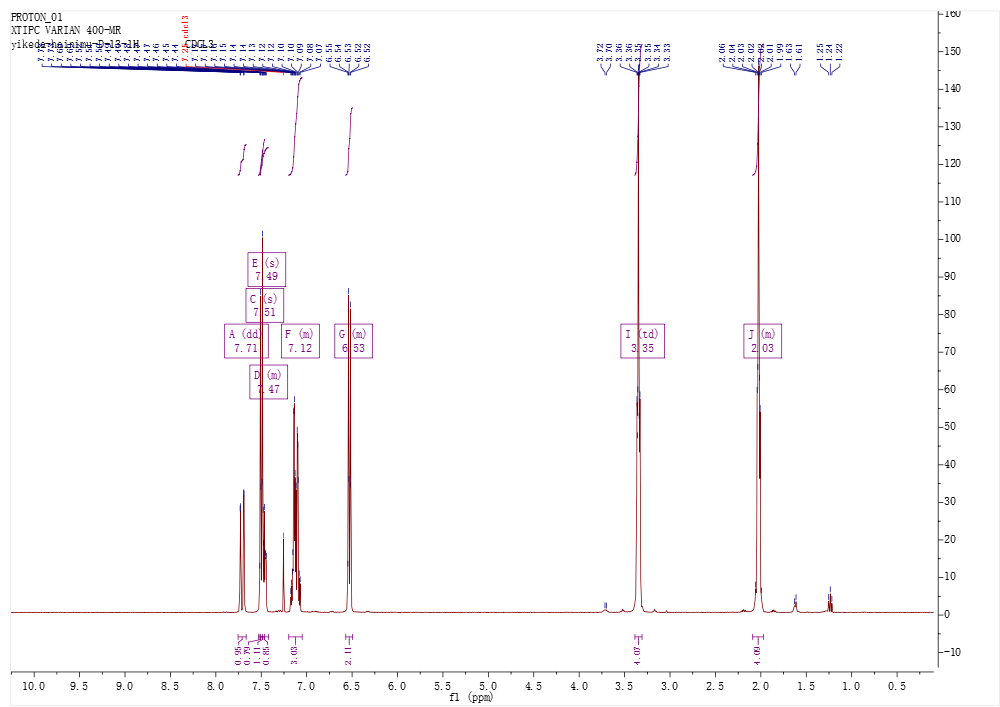
**

**
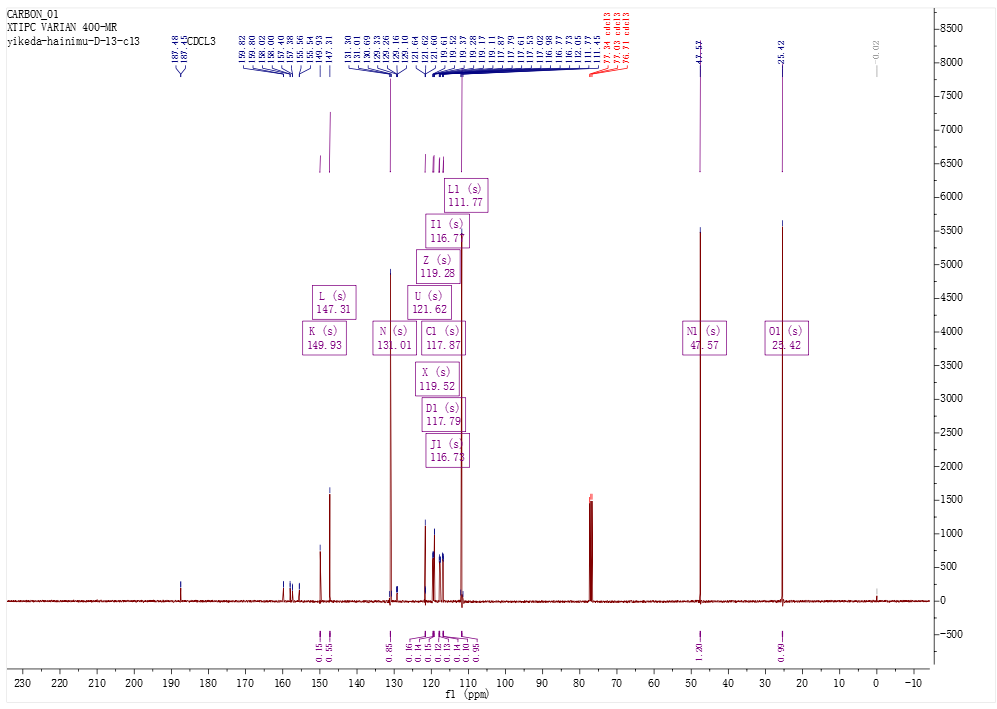
**

**Compound D1**


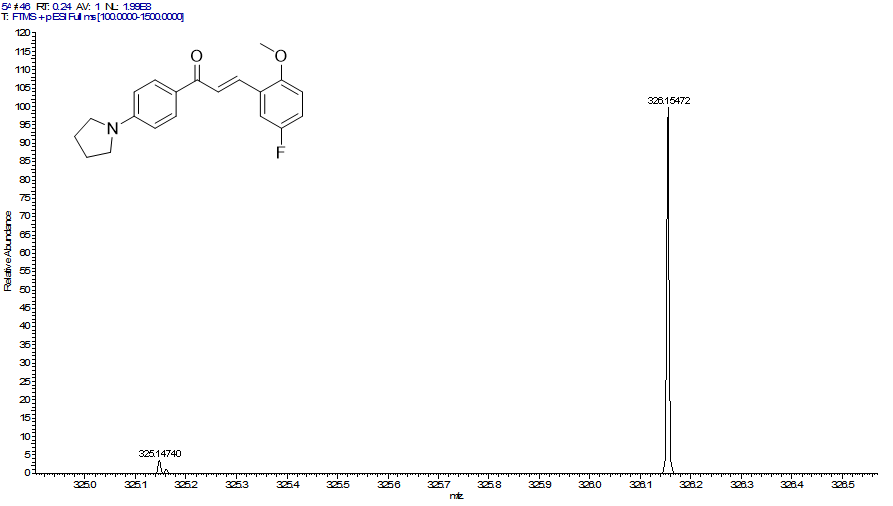


**
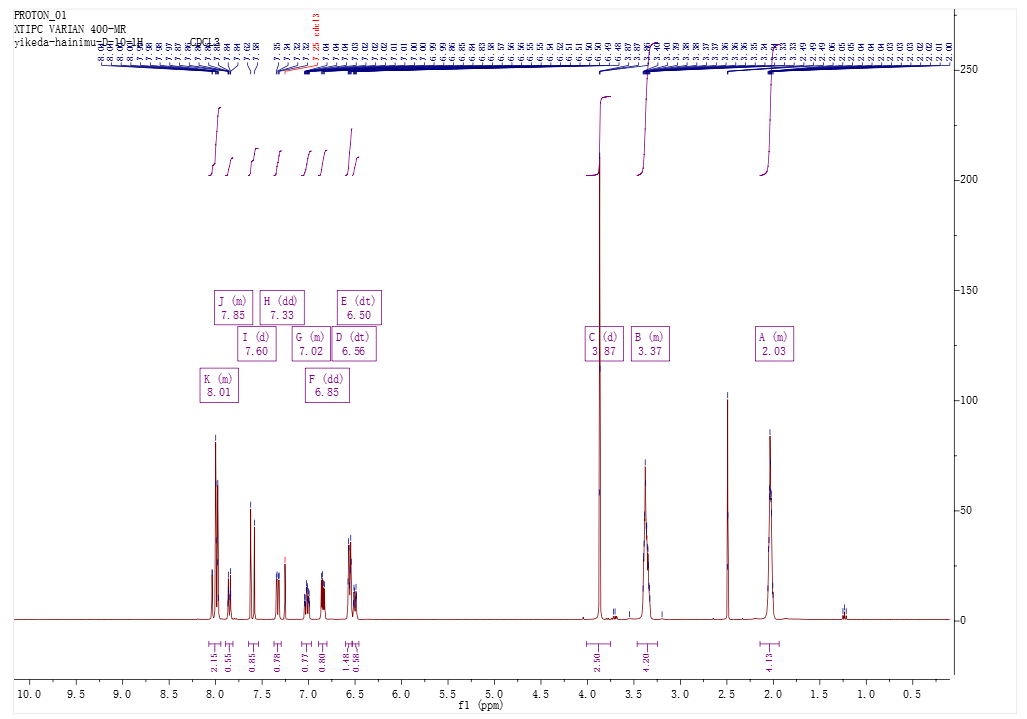
**

**
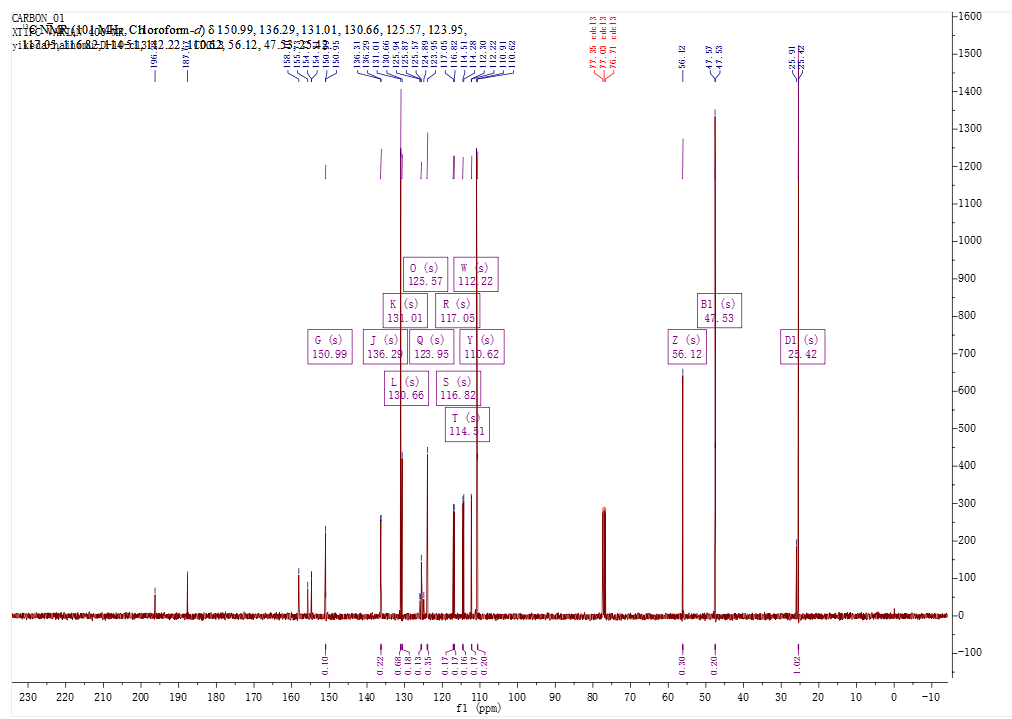
**

**Compound D2**


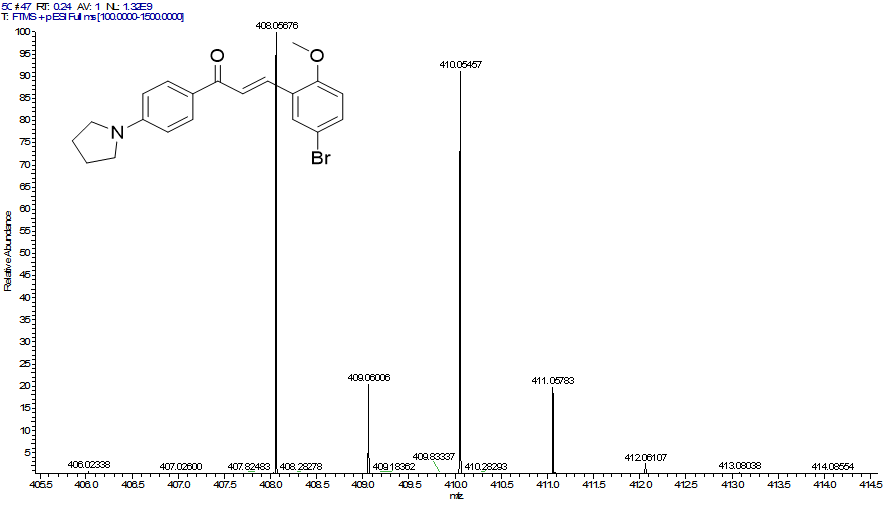


**
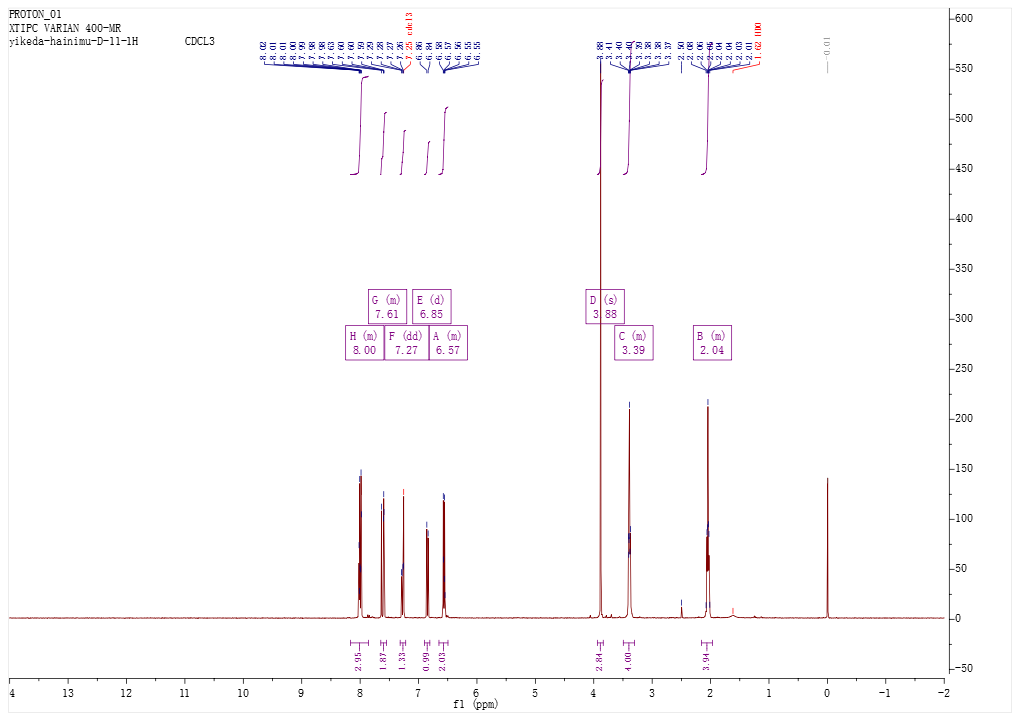
**

**
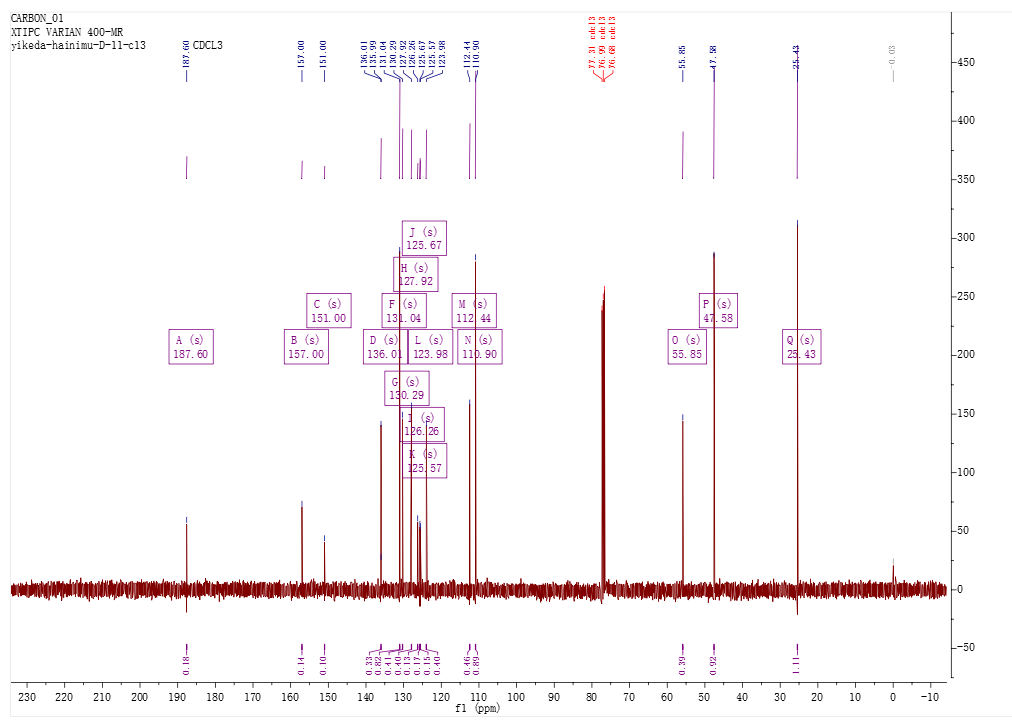
**

**Compound D3**


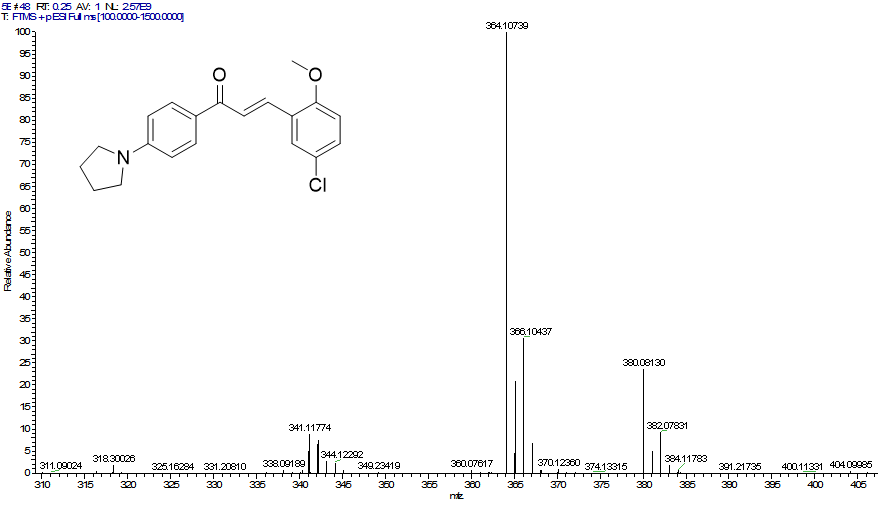


**
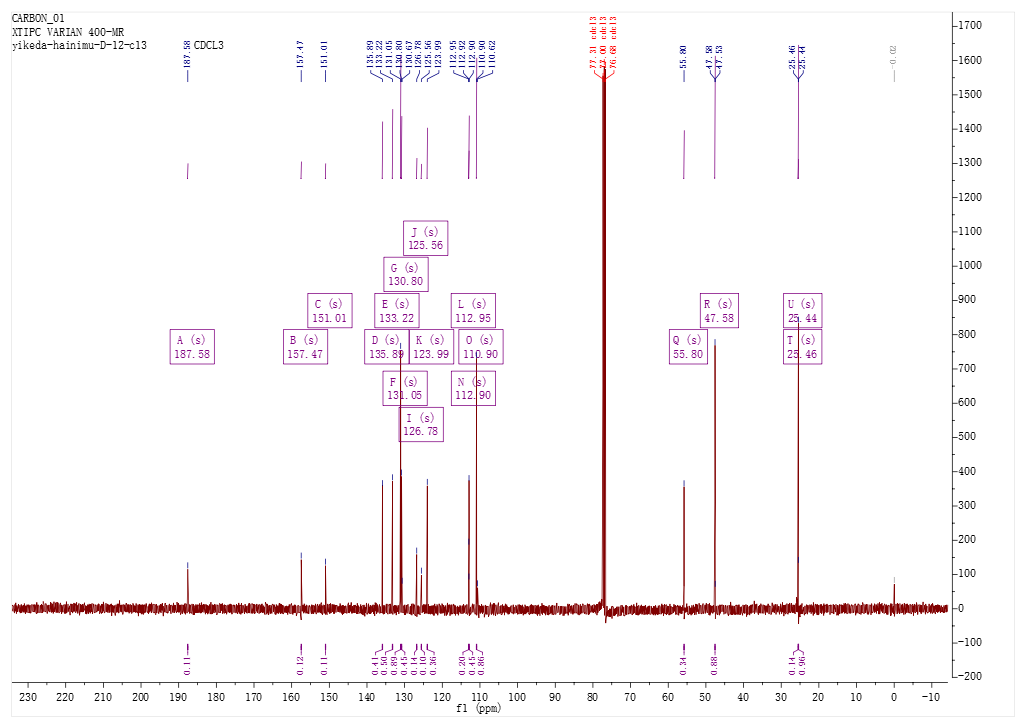
**

**
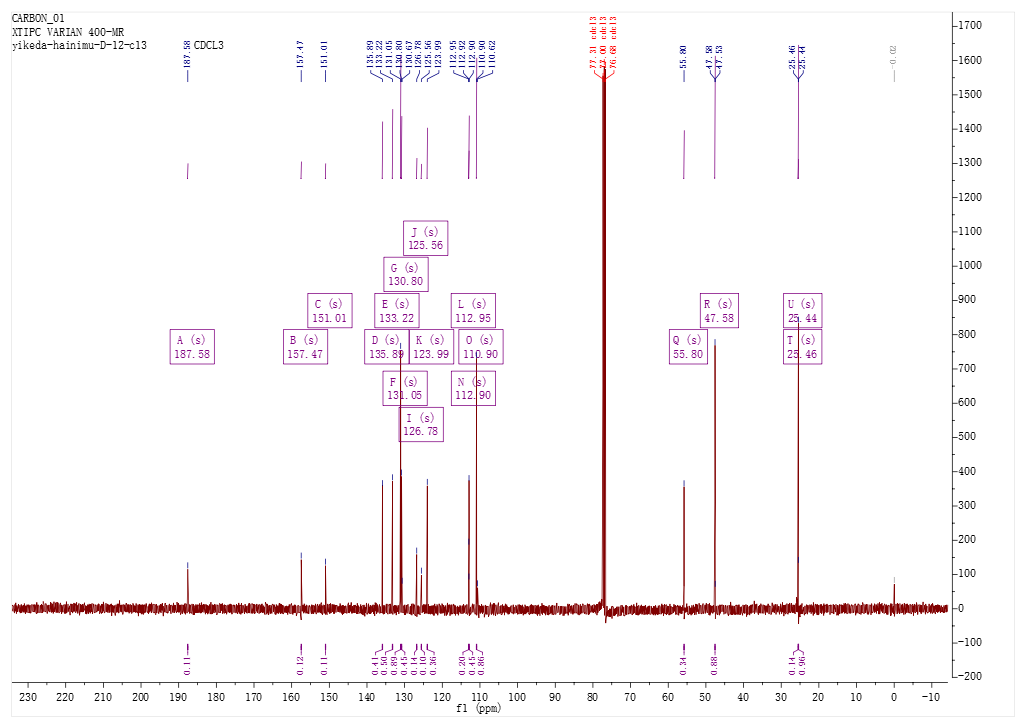
**

**Compound D4**


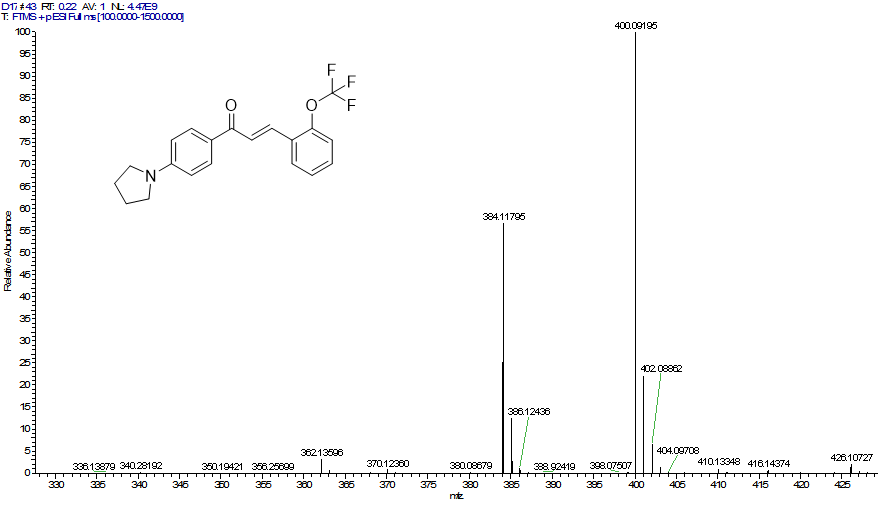


**
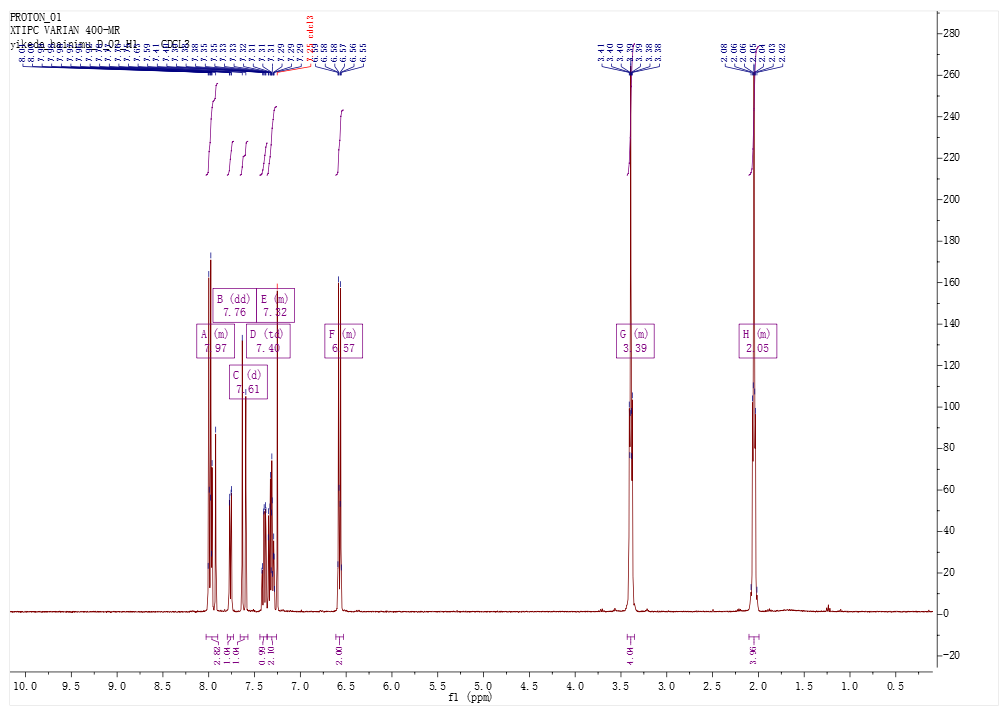
**

**
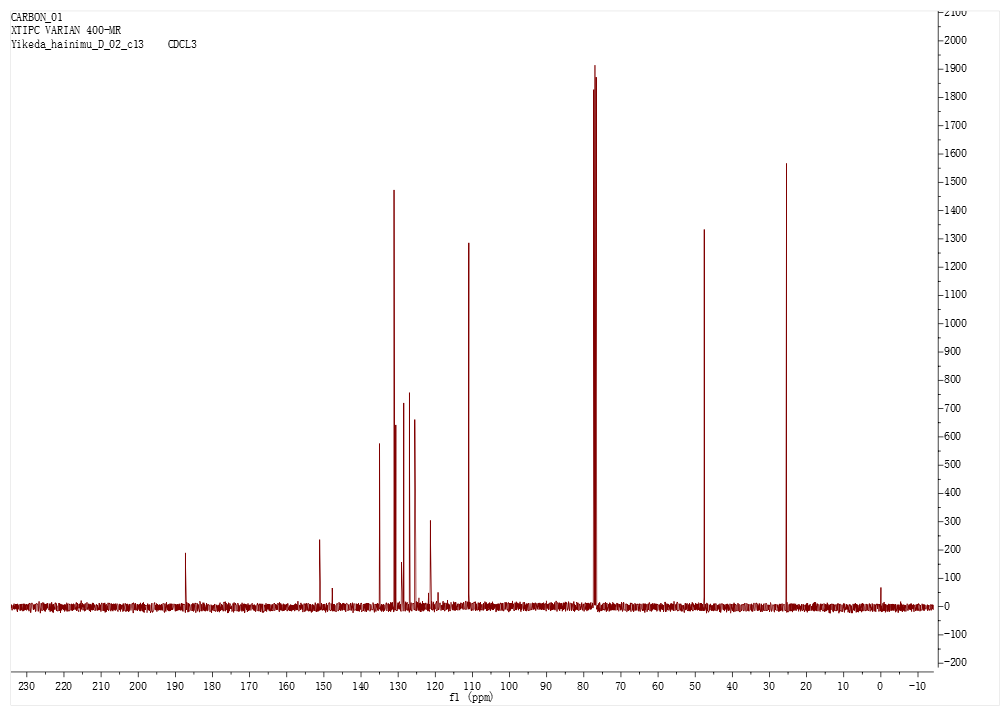
**

**Compound D5**


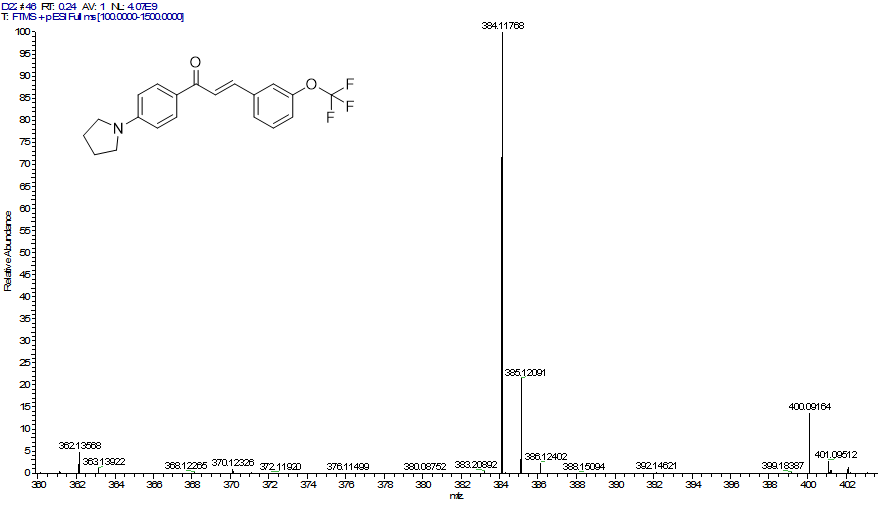


**
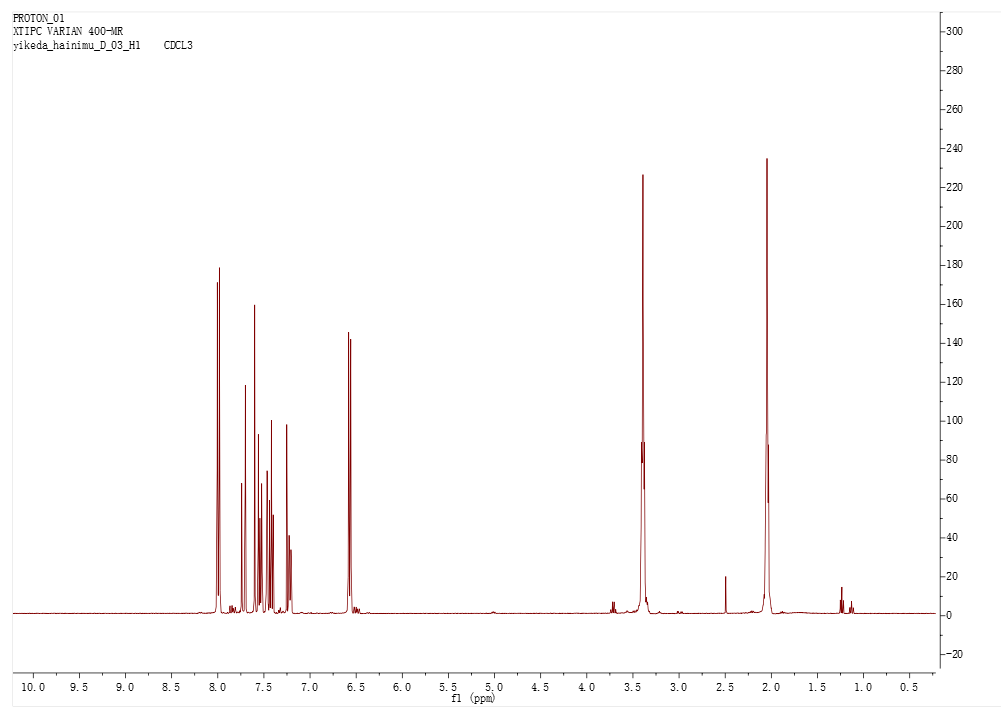
**

**
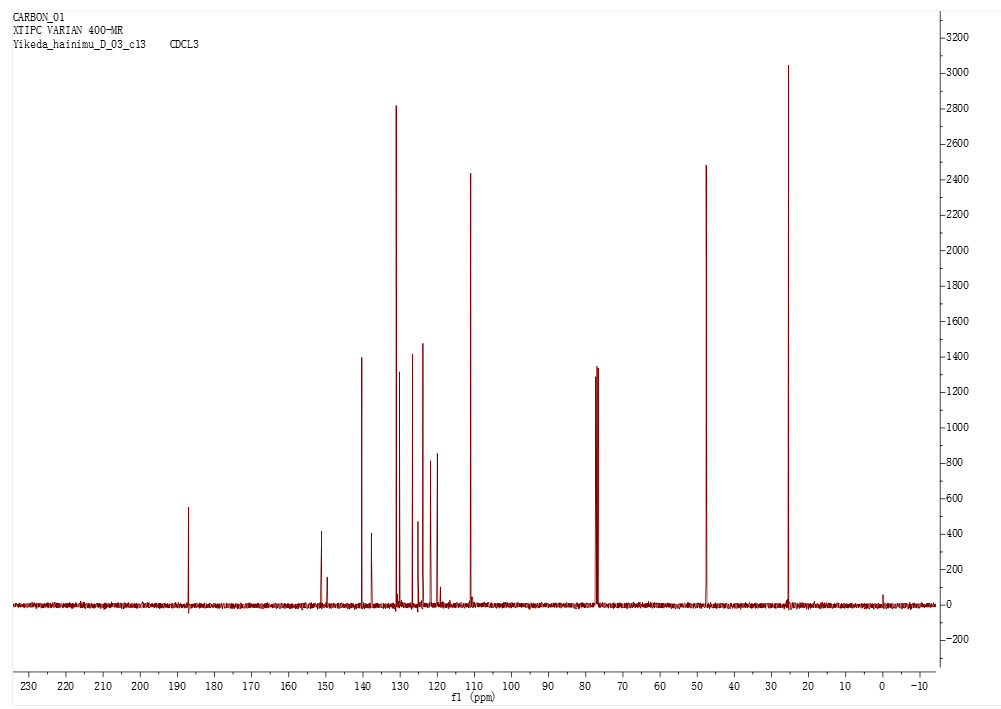
**

**Compound D6**


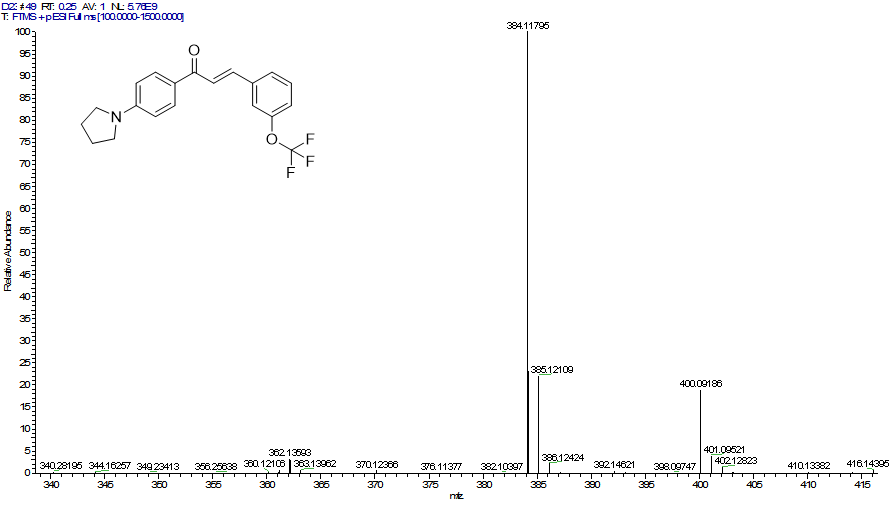


**
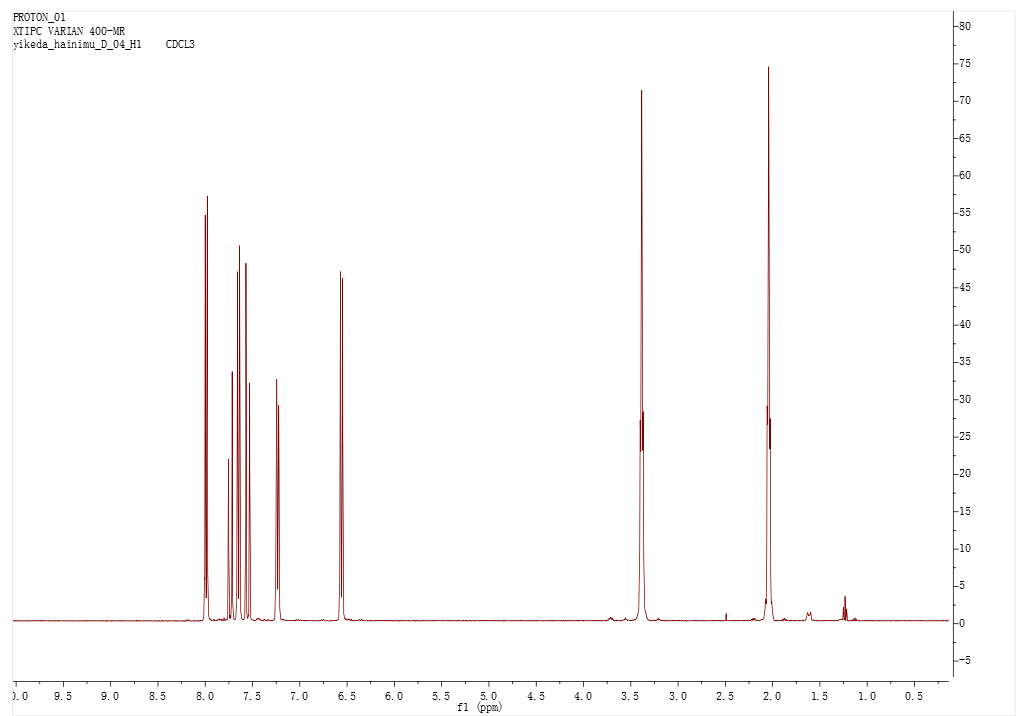
**

**
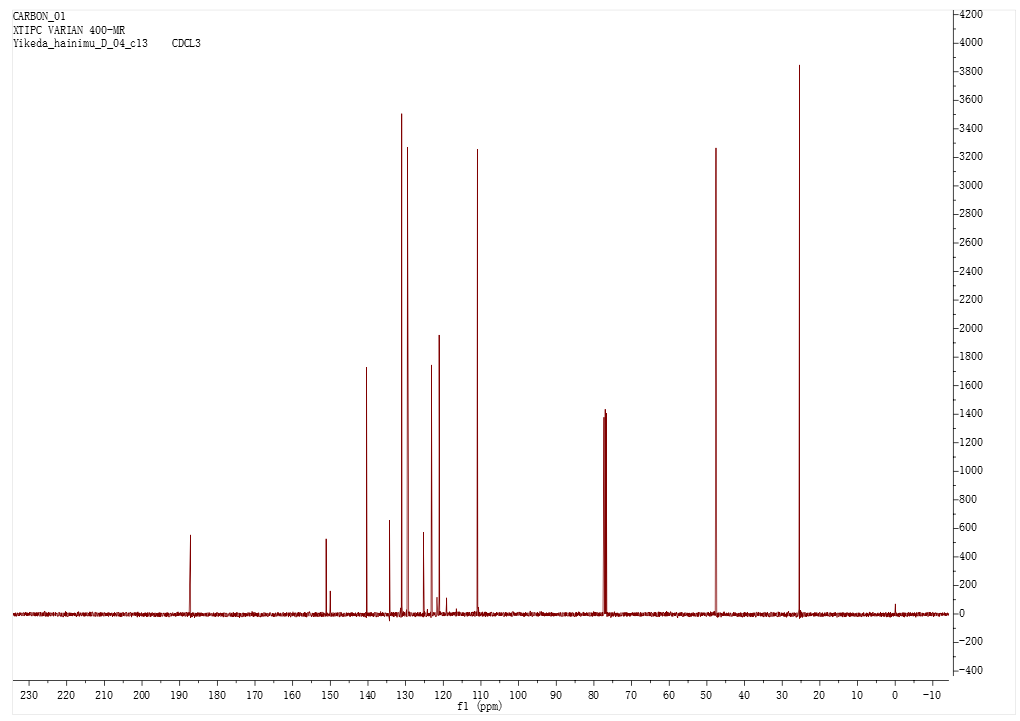
**
